# Small‐Molecule Activation of mRNA Translation by Click‐to‐Release Reaction in Cells

**DOI:** 10.1002/anie.202524223

**Published:** 2026-03-01

**Authors:** Tess Vosman, Friedrich Burba, Martin Sumser, Milan Vrabel, Hannes Mikula, Andrea Rentmeister

**Affiliations:** ^1^ Department of Chemistry Ludwig‐Maximilians‐Universität (LMU) München Munich Germany; ^2^ Institute of Organic Chemistry and Biochemistry of the CAS Prague 6 Czech Republic; ^3^ Institute of Applied Synthetic Chemistry Vienna Austria

**Keywords:** 5′ cap, click chemistry, mRNA, tetrazines, translation

## Abstract

mRNA is an emerging medical modality, however, approaches to control its activity lack behind other biologics. Bioorthogonal click‐to‐release reactions enable breaking chemical bonds at high reaction rates even in living cells to release a functionally active biomolecule (“uncaging”). We developed a 5′ cap modified with a *trans*‐cyclooctene (TCO‐cap) that reacts with hydroxyaryl‐tetrazines to efficiently release the native cap 0. This strategy is compatible with in vitro transcription and facilitates HPLC‐based purification of the resulting TCO‐capped mRNA, circumventing the need to digest uncapped mRNA produced in the process. Using eGFP‐ and luciferase‐mRNAs in mammalian cells, we show that TCO‐capped mRNAs are translationally muted and can be activated for translation by addition of cell‐permeable, non‐toxic sulfonamide‐modified hydroxyphenyl‐tetrazines. This work presents a new approach for small‐molecule‐induced translation in eukaryotes with potential to be applicable to any mRNA.

## Graphical abstract

A new method to control mRNA activity using bioorthogonal click‐to‐release reactions is presented. The 5′ cap is modified with a *trans*‐cyclooctene (TCO) that quickly reacts with hydroxyaryl‐tetrazines and efficiently releases the native cap 0. TCO‐capped mRNAs are initially translationally inactive but can be activated with non‐toxic, cell‐permeable tetrazines. This strategy offers a versatile way to regulate mRNA translation in eukaryotic cells.

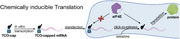

## 1 Introduction

**FIGURE 1:**
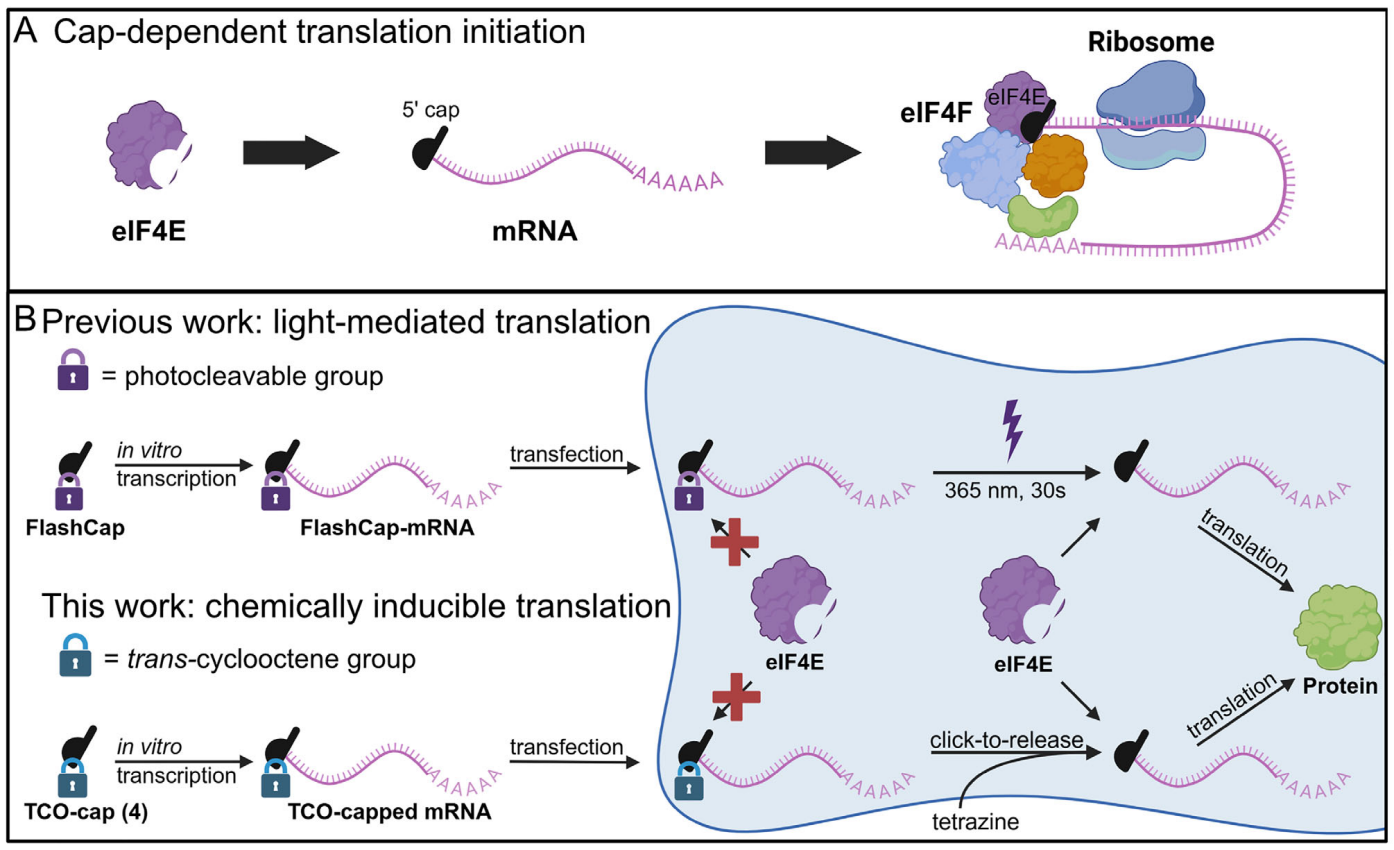
Concept for activation of mRNA translation with tetrazines in the context of previous work. (A) Cap‐dependent translation initiation starts with binding of the eukaryotic translation initiation factor 4E (eIF4E) to the 5′ cap of an mRNA. After binding of eIF4E, the eIF4F complex assembles and the closed‐loop formation is attained, allowing the ribosome to bind and translation to start. (B) Chemical modifications of the 5’ cap block mRNA translation. In previous work, FlashCaps were developed to activate translation by light. FlashCaps contain a photocleavable protecting group that can be removed by light (upper part). In this work, the TCO‐cap was developed to activate translation by small molecules. This 5’ cap analog is modified with a TCO moiety suitable for click‐to‐release chemistry. Upon reaction with suitable cell‐permeable, non‐toxic tetrazines, mRNA with a native 5’ cap 0 is released, enabling initiation of translation. Parts of this figure were created in Biorender (https://BioRender.com/h7kccw4 and https://BioRender.com/8aind0z).

mRNA has become a relevant medical modality that is produced in vitro in gram‐scale and applied to cells and in vivo [1, 2, 3]. The introduction of a 5′ cap structure and internal chemical modifications, like m^5^C and m^1^Ψ, have proven fundamental to increase the amount of protein produced and to reduce immunogenicity [4, 5]. Both endogenous and ectopic mRNAs are translated in the cytoplasm of cells. The canonical pathway is cap‐dependent and initiates by binding of the eukaryotic translation initiation factor 4E (eIF4E) to the 5′ cap, followed by the assembly of the multicomponent eIF4F complex, ribosome assembly, scanning and then translation (Figure 1A).

The field of bioorthogonal chemistry has advanced from connecting molecules via biocompatible click ligation to disconnecting bonds through efficient click‐to‐release chemistry [6, 7]. The tetrazine‐mediated cleavage of *trans*‐cyclooctene (TCO)‐modified compounds stands out due to its versatility, biocompatibility and ability to tune reaction rates and release efficiency by modification of the reactants. Most widely used is the so‐called release‐TCO (rTCO), which is characterized by connecting the TCO via an allylic hydroxy group in axial position to an amino group of the biomolecule of interest via a carbamate linkage [6]. TCO‐centered developments of the click‐to‐release reaction include additional methylation of the carbamate‐N to avoid formation of a dead‐end isomer [8] or the development of bicyclic TCOs, such as the dioxolane‐fused cleavable TCO (dcTCO) [9] or (*E*)‐bicyclo[6.1.0]non‐4‐ene (sTCO) [10, 11].

The substituents of the tetrazine also affect the reaction rate and release properties of the click‐to‐release reaction. Until recently, there was a trade‐off between click reaction rates and release efficiency, as substituents that enable efficient release show only low click reactivity [6]. Recently, the Mikula group developed tetrazines, which combine high click reactivity and release efficiency by introducing hydroxy groups onto phenyl‐ and pyridyl‐substituted tetrazine scaffolds [12]. An additional improvement was achieved by sulfonation, reported by the Vrabel and Mikula groups [13]. Although their click reactivity is lower compared to pyridyl‐substituted analogs, sulfonated and sulfonamide‐modified hydroxyaryl‐tetrazines exhibit faster release rates, enabling complete cleavage of the caged molecule within minutes [13].

In the context of cells, click‐to‐release chemistry now enables the rapid release of prodrugs on demand [7, 12, 13, 14]. The potential to uncage biomolecules has been realized and implemented to activate protein function in *E. coli* cells [15, 16]. It has also been exploited to achieve selective activation of prodrugs in diseased cells by functionalization of either the TCO‐caged moiety or tetrazine all the way to in vivo applications [17, 18, 19]. Its application in antibody‐drug conjugates is currently pursued in clinical studies [20, 21]. However, little work has been done for click‐to‐release chemistry in the context of RNA [22, 23] and we are not aware of its application to mRNA.

Compared to small molecules and even antibodies, mRNA is a relatively new modality. As a consequence, while the concept of activating small molecule prodrugs and antibody‐drug conjugates has been followed for decades, the idea of controlling mRNA activity is quite new. We have recently developed FlashCaps as a way to generate muted mRNA that can be activated for translation by light (Figure 1B). The concept was validated in vitro, in mammalian cells and in vivo [24, 25, 26, 27, 28]. Photocleavable protecting groups have also been attached to different positions of the 5′ cap and served to facilitate purification [29, 30] and identify cap‐binding partners [31].

However, while FlashCaps provide a great proof‐of‐concept for activation of translation by an external trigger, the application of light for therapeutic purposes is still limited. Problems include background signal from inadvertently uncaging by exposure to light during the experiment, limited tissue penetration of light preventing application in most parts of the intact body, and even reproducible dosing of light in cells and in vivo using different irradiation setups.

We therefore aimed to devise a strategy to control and activate translation of mRNA by reaction with a small molecule trigger. This would allow to control (*i*) the dosing of “active” mRNA without repeated administration and (*ii*) the expression kinetics, for example, of antigens and adjuvants, using small molecule drugs. Smart mRNA vaccines containing various layers for post‐transcriptional gene regulation were already proposed more than 10 years ago [32]. The recent remarkable improvements in the field of mRNA therapeutics now substantiate such ideas. In particular, the stability of mRNA—known as a key bottleneck—has very recently been markedly increased by circularization [33, 34, 35], self‐amplifying mRNA [36, 37], and incorporation of RNA stability enhancers [38] leading to reported mRNA stabilities over 2 weeks in vivo. The concept of controlling translation in a programmable way also has potential for the construction of artificial gene circuits in synthetic biology [39].

In this work, we developed a 5′ cap modified with a TCO at the *N* ^2^‐position of m^7^G to mute translation via blocked eIF4E‐binding (Figure 1B). We validated that this TCO‐cap was incorporated into mRNA by in vitro transcription and impeded translation of the resulting mRNA. Upon reaction with a suitable tetrazine, the native cap 0 was rapidly liberated. We then demonstrated that the state‐of‐the‐art hydroxyaryl‐tetrazines enable click‐to‐release reaction with mRNA and that this reaction triggered translation of the liberated cap 0‐mRNA in cells (Figure 1B).

## 2 Results and Discussion

### 2.1 TCO‐Cap Enables Rapid Release of Cap 0 by Click‐to‐Release Reaction With Hydroxyaryltetrazines

**FIGURE 2:**
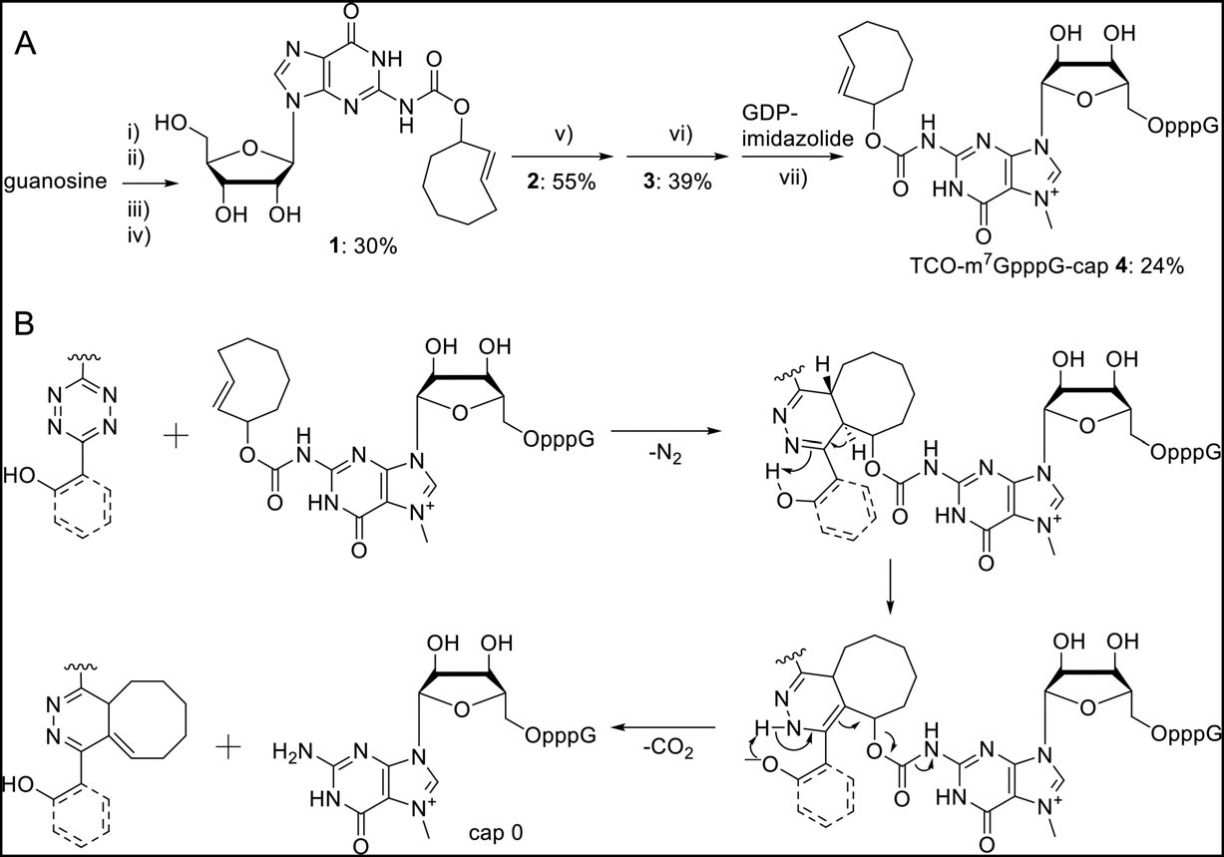
Synthesis of the TCO‐cap and click‐to‐release reaction to liberate the native cap 0. (A) Synthesis of the TCO‐cap (**4**): The four steps of chemical synthesis of mRNA 5′ caps are attachment of the cleavable moiety to guanosine, phosphorylation at the 5′ position, methylation at the *N*7 site and coupling with guanosine diphosphate (GDP). (i) Trimethylsilylchloride, 4‐(dimethylamino)pyridine, pyridine, dichloromethane, (ii) COCl_2_ (15wt% solution in toluene), (iii) Axial *trans*‐cyclooct‐2‐en‐1‐ol, (iv) Tetrahydrofuran/NH_4_OH 19:1, (v) POCl_3_, trimethylphosphate, (vi) Iodomethane, DMSO, (vii) ZnCl_2_, GDP‐imidazolide, DMF. (B) Reaction scheme for intended click‐to‐release reaction with *ortho*‐hydroxyaryl tetrazines used in this study. First, TCO and the tetrazine react in an inverse electron‐demand Diels–Alder cycloaddition accompanied by the loss of nitrogen. The click product undergoes isomerization mediated by the *ortho*‐hydroxy group of the former tetrazine. Finally, the elimination cascade leads to cleavage of the carbamate linkage to release the m^7^GpppG (i.e., cap 0) molecule. Both the click and the cleavage reaction are heavily dependent on the tetrazine used.

To develop a translationally muted mRNA that can be chemically induced by the click‐to‐release reaction between TCO and tetrazines, we synthesized a 5′ cap 0 (i.e., m^7^GpppG) modified with a cleavable TCO at the *N* ^2^‐position of m^7^G (Figure 2). Normally, the *N* ^2^‐amino group forms a hydrogen bond with residue E103 of eIF4E upon binding (Figure S2). By modifying this position with the bulky TCO group, we aimed to disrupt the interaction of eIF4E with the Watson‐Crick site of m^7^G and introduce steric clashes, thereby inhibiting binding of eIF4E to the TCO‐modified 5′ cap. To facilitate the release, we connected the axial isomer of release‐TCO at its allylic position to the *N* ^2^‐position of m^7^G via a carbamate linkage. Synthesis started with protection of guanosine at the hydroxy groups as trimethylsilyl ethers, followed by activation of the *N* ^2^‐position with phosgene to form the isocyanate, which was reacted with the axial isomer of *trans*‐cyclooct‐2‐en‐1‐ol, prepared and purified as previously described [40, 41] (Figures 2A and S1). After deprotection by reaction with NH_4_OH, the TCO‐modified guanosine (**1**) was obtained in 30% yield as an off‐white solid. Subsequent steps include phosphorylation at the 5′ end with POCl_3_ to TCO‐GMP (**2**), methylation at the *N* ^7^‐position to TCO‐m^7^GMP (**3**) and finally coupling to guanosine diphosphate (GDP) imidazolide to the desired TCO‐m^7^GpppG (**4**, TCO‐cap) (Figures 2A and S1), which was characterized by HPLC, HRMS and NMR (Suppl. Figures S28, S35, S65–S69).

Upon reaction with suitable tetrazines, TCO‐cap (**4**) undergoes a click‐to‐release reaction (Figure 2B) [10, 12, 13, 15]. First, the tetrazine reacts with the TCO moiety in an inverse electron‐demand Diels–Alder cycloaddition (IEDDA), accompanied by the loss of N_2_ (Figure 2B). Using a tetrazine with an *ortho*‐hydroxy group, this click product undergoes isomerization, followed by an elimination cascade, leading to cleavage of the carbamate and subsequent release of the m^7^GpppG (i.e., cap 0) molecule.

**FIGURE 3:**
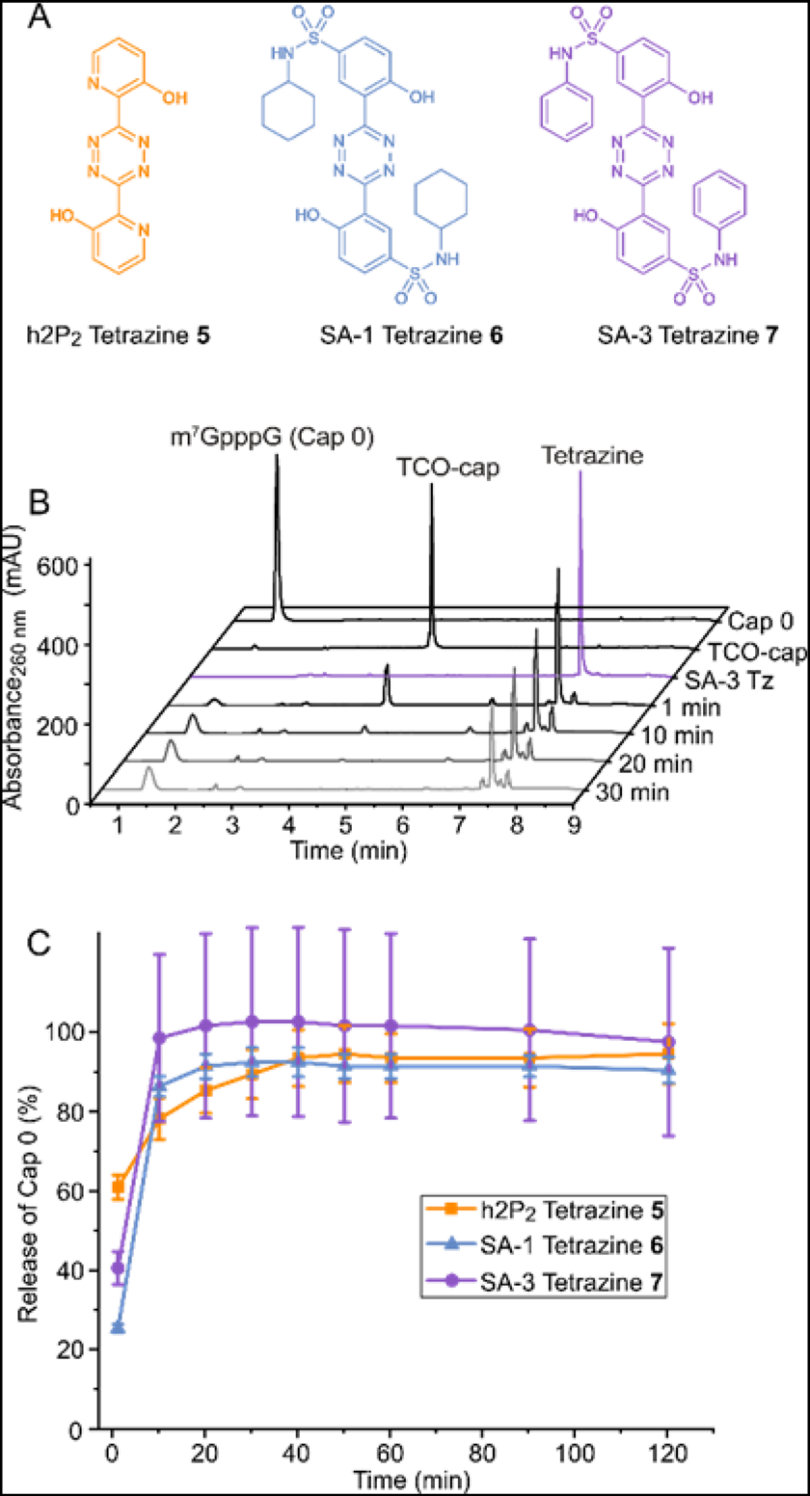
Click‐to‐release reaction of the TCO‐cap with indicated tetrazines in vitro. (A) Structures of key tetrazines used in this study. Electron‐withdrawing groups on the Tz accelerate the click reaction while donating groups slow it down. A common structural feature among **5** to **7** are *ortho*‐hydroxy groups which boost the rearrangement and cleavage to restore cap 0. (B) Representative HPLC traces of the click‐to‐release reaction in sodium phosphate buffer in vitro using 3.3 eq. of tetrazine SA‐3 over 30 min at 37°C. The TCO‐cap is rapidly consumed by the Tz while the transient click‐product is formed (peak at 6.2 min). This then degrades to form cap 0. (C) Kinetic study of the click‐to‐release reaction of the TCO‐cap and indicated tetrazines in MEM medium (mean values and standard error (SE) for *n* = 3 independent experiments are shown). Conditions: 60 µM TCO‐cap, 200 µM Tz in cell culture medium (MEM) containing 10% DMSO; trihydroxyphenyl coumarin as internal standard. Quantification of the released cap 0 by external calibration.

To assess the click‐to‐release reaction of the TCO‐cap, we tested three *ortho*‐hydroxyaryl‐substituted tetrazines, previously reported to achieve cell‐compatible accelerated click‐to‐release of rTCO‐caged compounds (Figure 3A) [12, 13]. The *ortho*‐hydroxy groups are crucial for the release mechanism as they promote the tautomerization required for the elimination cascade by protonation of the pyridazine nitrogen (Figure 2B). Tetrazine **5** (h2P_2_) [12] bears an activating pyridyl group, which leads to a higher click reactivity in the inverse electron‐demand Diels–Alder reaction. On the other hand, **6** (SA‐1) and **7** (SA‐3) benefit from increased pK_a_ of the phenolic group to accelerate the elimination step and thus drive the release reaction [13].

To test the release, we reacted TCO‐cap (**4**) (60 µM) with 3.3 eq. of the respective tetrazine (**5**–**7**) in sodium phosphate buffer or cell culture medium (MEM) and analyzed the reaction after different periods of time (Figures 3B and S4–S9). For SA‐3 (**7**) in MEM medium, we observed immediate depletion of TCO‐cap (**4**) accompanied by formation of two new peaks (*t*_R_ ∼1.25 and *t*_R_ ∼6.20 min), out of which one was the released cap 0 (*t*_R_ = 1.25 min) (Figure 3B), as identified by the reference compound and LC‐MS (Figure S11). The smaller peak at 6.20 min was assigned to the click product of the TCO‐cap with SA‐3 (Figure 3B). It is prominent at early timepoints (1 min, 10 min) but then reduced and not detectable when the release is complete (30 min).

The time‐dependent release was tested for tetrazines **5**–**7** both in sodium phosphate buffer and in cell culture medium (Figure 3C and S4–S9). All three tetrazines led to almost complete (90%–100%) release of cap 0 over time in MEM medium (Figure 3C), as confirmed by HPLC (Figure S4–S9) and LC‐MS analysis (Figure S11). However, the rate and efficiency of the release step differed slightly depending on the tetrazine and solution (phosphate buffer or MEM) used (Figure S10). Comparative evaluation in cell culture medium (MEM) showed that SA‐1 (**6**) and SA‐3 (**7**) reacted faster than **5**, leading to 97% of cap 0 after only 10 min and complete release at 20 min (Figure 3C). In direct comparison to tetrazines **6** and **7**, **5** exhibits higher click reactivity, as no traces of the TCO‐cap (**4**) are detected by HPLC even at the earliest timepoint (Figures S4,S5). However, the release and thus the liberation of cap 0 is slower for reactions with **5** (Figure 3C), compared to **6** or **7**. We observed 92% of cap 0 release after 40 min in MEM medium (Figure 3C). This trend is in line with previous reports about the kinetics and release of these tetrazines [12, 13]. We also tested the stability of TCO‐cap in cell lysate and found that it was not compromised compared to the native cap 0 (Figure S12 and Table S5). Finally, we tested the isomerization of TCO to CCO, recently reported in cell media [42], using TCO‐GMP (**2**) in MEM medium with and without FCS. The TCO‐GMP contained already 6%–7% CCO‐GMP (Table S6). Without FCS, we observed very little isomerization over 24 h (Figure S13), whereas in MEM medium with FCS we observed dephosphorylation and isomerization, with ∼20% total CCO‐G after 24 h (Figure S14 and Table S6).

### 2.2 TCO‐Cap is Compatible With In Vitro Transcription and Facilitates HPLC‐Based Purification of mRNAs

**FIGURE 4:**
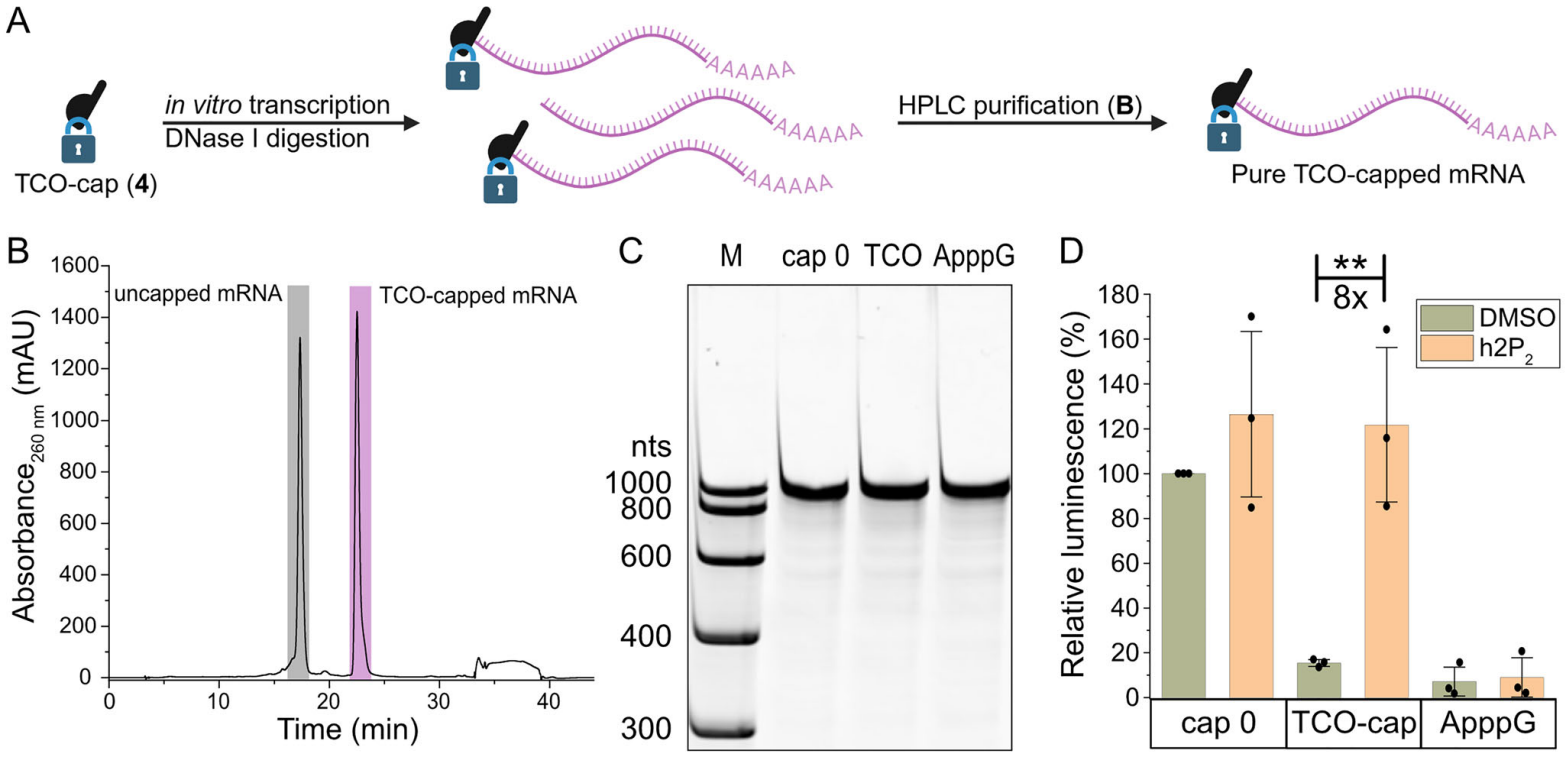
Production, Purification, and Characterization of TCO‐capped mRNA in vitro. (A) The TCO‐cap is compatible with in vitro transcription (IVT) and facilitates purification of TCO‐capped‐mRNAs. Unwanted uncapped RNA of the same length is normally difficult to separate. The hydrophobic TCO‐cap increases retention time on reversed‐phase HPLC and allows for purification. (B) Reversed‐phase preparative HPLC of IVT reaction for *Gaussia* luciferase (GLuc) mRNA (∼800 nt). Two base‐line‐separated peaks are obtained for the uncapped RNA (first peak) and the TCO‐capped mRNA (second peak). (C) Gel electrophoresis of differently capped GLuc‐mRNAs with indicated 5′ caps (cap 0‐, TCO‐ and ApppG) to show length and integrity (7.5%, denat. PAA gel). (D) Luminescence measurement of differently capped GLuc‐mRNA after in vitro translation (mean values and SE for *n* = 3 independent experiments are shown. ** = *P*<0.01, *p*‐value for TCO‐capped mRNA treated with DMSO versus h2P_2_ is 9.8x10^−3^, Student's *t*‐test). High luminescence indicates high protein output. Before in vitro translation, the mRNA was either treated with DMSO (green bars) or h2P_2_ (**5**, 7.5 equivalents; yellow bars) for 1 h at 37°C. Parts of this figure were created in Biorender (https://BioRender.com/fusvrhs).

Next, we wanted to know whether the TCO‐cap (**4**) is compatible with in vitro transcription (IVT) to produce TCO‐capped mRNA (Figure 4A). To this end, we performed IVT reactions of DNA templates coding for *Gaussia* luciferase (GLuc), *Renilla* luciferase (RLuc), or enhanced green fluorescent protein (eGFP) in vectors containing the T7 promotor and a poly(A) tail. The 5′ cap is added in addition to the NTPs and a mixture of capped and uncapped RNA is obtained as a result of transcriptional priming by the 5′ cap or GTP, respectively. Normally, we digest uncapped RNA enzymatically [43], because long capped and uncapped RNAs (i.e., ∼1000 nts) are not separated by reversed‐phase HPLC. However, recently the hydrophobic effect of a 5′ cap modification with a photocleavable group was reported to enable the efficient chromatographic separation of capped and uncapped RNA [29]. We therefore tested whether the TCO‐capped‐mRNA would have a similar effect. To our delight, the chromatogram showed very good separation, allowing us to obtain pure TCO‐capped mRNAs by reversed‐phase HPLC (Figure 4B). As controls, we produced the same mRNAs with different 5′ caps, that is, the cap 0 as positive control and ApppG as negative control. For these, the uncapped RNA was removed by enzymatic digestion as previously described [43], before HPLC purification. All mRNAs were obtained in good yields (for the TCO‐cap 1–2 µg/50 µL transcription) and purities (Figures 4C and S19).

Finally, we wanted to see whether the TCO‐capped mRNAs are translationally muted and can be activated by addition of tetrazines. As a proof‐of‐concept, we performed an in vitro translation assay with differently capped luciferase RNAs (Figures 4D). Indeed, the TCO‐capped‐mRNA was barely translated (15%) compared to the respective cap 0‐mRNA. The negative control with an ApppG‐cap shows that 7% are a result of cap‐independent translation for this construct. The addition of tetrazine **5** (h2P_2_) increased the translation (8‐fold), resulting in similar luminescence values as for the positive control, that is, the cap 0‐mRNA. Importantly, addition of tetrazine had no effect on the negative control (ApppG‐mRNA) (Figure 4D).

### 2.3 Translation of TCO‐Capped mRNA Can Be Activated by Hydroxyaryltetrazines in Cells

Encouraged by this positive result, we proceeded to test the click‐to‐release reaction in cells, aiming to achieve small‐molecule activation of mRNA translation. To ensure that the tetrazines are also suitable for cell studies, we performed cell viability tests. Incubation of HeLa cells, transfected with TCO‐capped GLuc‐mRNA, with tetrazines **5**–**7** (10 µM) only reduced the viability in an MTT assay for h2P_2_ (67%, Figure S15), indicating that SA‐1 (**6**) and SA‐3 (**7**) are not toxic at this concentration and therefore more suitable for cell experiments than h2P_2_ (**5**). Considering that SA‐1 (**6**) and SA‐3 (**7**) also showed better release kinetics in MEM medium (Figure 3C), we used these compounds in cell studies.

**FIGURE 5:**
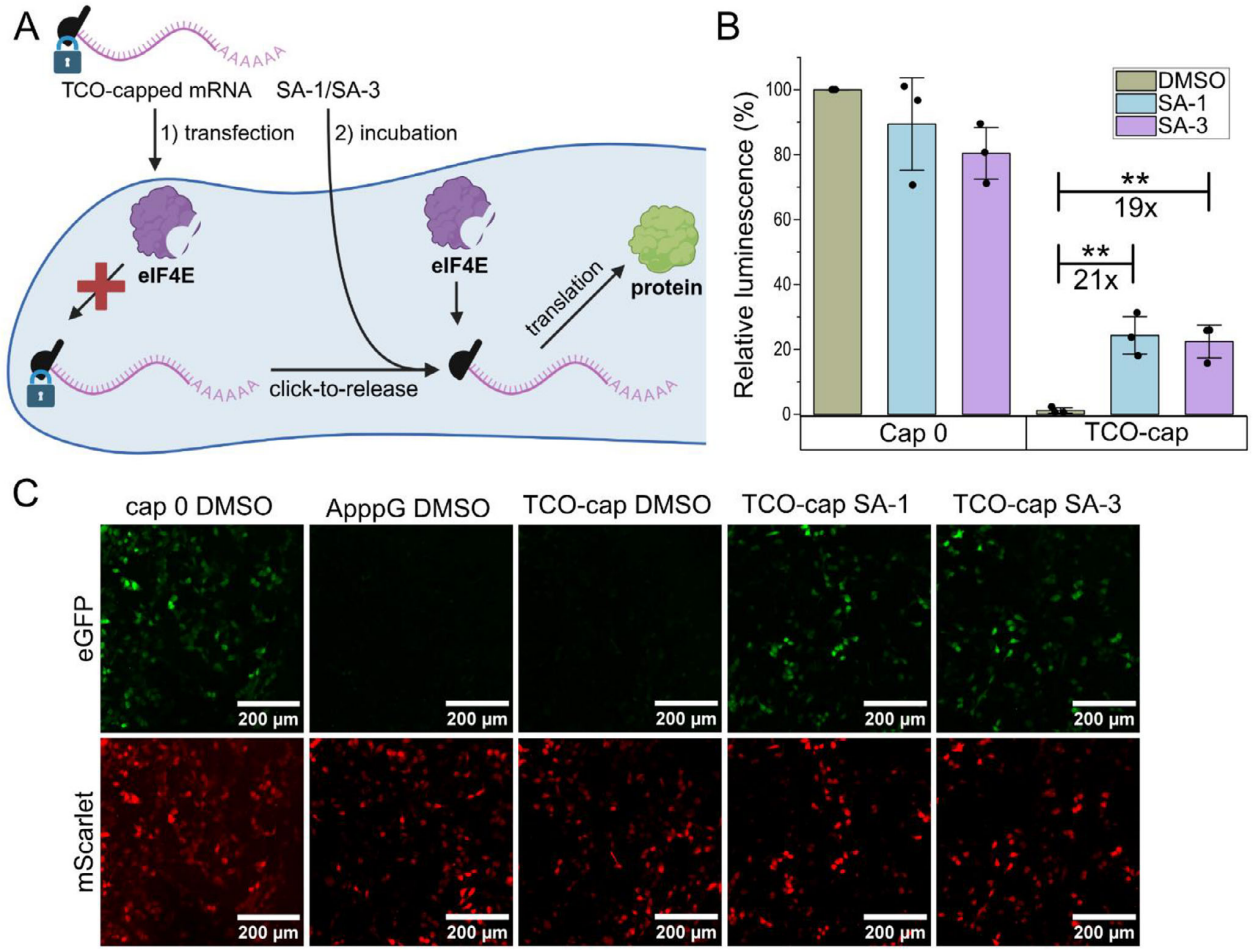
In‐Cell Activation of TCO‐capped mRNAs for Translation by Tetrazines. (A) Experimental scheme: The in‐cell activation of TCO‐capped mRNA was assessed after transfection of HeLa cells with capped mRNAs (GLuc or eGFP), followed by addition of cell‐permeable tetrazines to start an intracellular click‐to‐release reaction. The protein output was determined by luminescence measurement or microscopy. (B) Luminescence measurements for cells transfected with differently capped GLuc‐mRNA and addition of tetrazines SA‐1/SA‐3 or DMSO as control (mean values and SE for *n* = 3 independent experiments are shown. ** = *P*<0.01, *p*‐value for TCO‐capped mRNA treated with DMSO versus SA‐1 is 4.0x10^−3^, DMSO versus SA‐3 is 3.5x10^−3^, Student's *t*‐test). Cap‐independent translation (Luminescence observed using ApppG‐capped mRNA) has been deducted. (C) Microscopy images of cells that were co‐transfected with differently capped mRNA (eGFP) and cap0‐mScarlet‐mRNA as transfection control and then treated with tetrazines SA‐1/SA‐3 or DMSO as control. Shown is one representative set of *n* = 3 experiments. Other replicates are shown in Figures S22–S25. Parts of this figure were created in Biorender (https://BioRender.com/njapdqp).

We transfected HeLa cells with HPLC‐purified differently capped GLuc‐mRNAs (Figure 5A). At 4 hours after transfection, the cells were treated with 10 µM tetrazine (**6** or **7**) in MEM medium, containing 0.1% DMSO for 1 h, then put back in normal medium. The control was treated with 0.1% DMSO solution without tetrazine. The translational output was determined the next day by luminescence measurements from the supernatant, as *Gaussia* luciferase is secreted. The relative luminescence values for the positive control (cap 0‐GLuc‐mRNA) without tetrazine was set to 100% (Figure 5B). To account for cap‐independent translation, the values for ApppG‐mRNA (i.e., cap‐independent translation) are deducted before normalization. For cap 0‐GLuc‐mRNA, addition of the tetrazines SA‐1 or SA‐3 slightly reduced the translational output (ca. 90%) (Figure 5B), most likely as a result of stress and/or low toxicity of the tetrazines. Compared to the cap 0‐mRNA, the TCO‐capped mRNA led to very low relative luminescence (1%, Figure 5B), indicating that the installation of the TCO at the *N* ^2^‐position of m^7^G of the 5′ cap efficiently blocked cap‐dependent mRNA translation in cells. After treatment with tetrazines, cells with TCO‐capped GLuc‐mRNA showed higher luminescence (Figure 5B), indicating that the click‐to‐release reaction works in cells and releases functional mRNA that becomes translated. For SA‐1 (**6**) and SA‐3 (**7**), the increase was 21‐fold and 19‐fold, respectively. Compared to cap 0‐mRNA, the relative cap‐dependent translation reached 22%–24% compared to the positive control (cap 0).

It is worth noting that the activated TCO‐capped mRNA reached a lower translational output compared to previously developed light‐activated FlashCap‐mRNAs (54%–72%) [27]. Possible explanations could be isomerization of the TCO group [42] (Figure S14 and Table S6) or limited access to the tetrazine. Indeed, tetrazines can be reduced in the cellular environment [44, 45] and cause non‐specific protein labeling [46], necessitating ongoing efforts to improved intracellular performance of bioorthogonal systems. On the other hand, the TCO‐capped mRNA is less leaky, that is, better translationally muted, compared to the unactivated FlashCap mRNA. For biological applications, this can be very important when unwanted formation of even a small amount of a protein needs to be avoided, because it is toxic or causes a strong background effect.

The experiment was repeated multiple times with slight variations of the conditions (different transfection times, tetrazine concentrations, DMSO concentration) in an attempt to optimize the activation. However, we observed that these variations did not have a large effect on the activation of the TCO‐capped‐mRNA (Figure S17). Although a higher dose of tetrazine seemed to have a slight negative effect on the translation of cap 0‐mRNA, potentially due to increased toxicity, the activation of TCO‐capped‐mRNA remained similar. Taken together, our results show a robust turn‐on of translation under various conditions.

To independently validate that translation of TCO‐capped mRNA can be activated by addition of tetrazines in cells, we also performed transfection with mRNAs coding for fluorescent reporter proteins followed by fluorescence microscopy for analysis (Figure 5C). To this end, we produced differently capped eGFP‐mRNAs as well as cap 0‐mScarlet‐mRNAs. It was previously reported that m^5^C and m^1^Ψ increase the amount of protein produced. [5, 47] We therefore prepared eGFP‐mRNAs with these modifications and found that they were compatible with the TCO‐cap for in vitro transcription and purification (Figure S18). HeLa cells were co‐transfected with these differently capped eGFP‐mRNAs and cap 0‐mScarlet‐mRNAs as internal control, followed by treatment with tetrazines SA‐1 or SA‐3 as described above. After 24 h, cells were fixed and analyzed by fluorescence microscopy (Figure 5C). As expected, cap 0‐eGFP‐mRNAs (positive control) showed bright green fluorescence whereas ApppG‐capped eGFP‐mRNAs gave no green fluorescence. The TCO‐capped eGFP‐mRNA did not give green fluorescence when treated only with DMSO. When the cells transfected with TCO‐capped eGFP‐mRNA were incubated with SA‐1 or SA‐3, the green fluorescence was very well detectable (Figure 5C). The mScarlet fluorescence was at a similar level in all samples and confirmed that only transfected cells (red) could be turned on for eGFP‐formation. These data indicate that tetrazine‐mediated click‐to‐release chemistry is occurring in cells on TCO‐capped mRNA and that this reaction can be used to activate translation. Furthermore, the microscopy data confirms the results obtained with the luciferase‐mRNAs. Microscopy images with all controls (including cap 0‐ and ApppG‐eGFP‐mRNA treated with tetrazines) are provided in the supplementary information (Figures S20–S25).

## 3 Conclusion

In summary, we developed a chemical biology approach to produce translationally muted mRNAs that can be activated by a click‐to‐release reaction. We validated the concept on the molecular level in vitro and on the level of translation in vitro and in mammalian cells. To the best of our knowledge this presents the first example of small‐molecule activation of ectopic mRNAs in cells. Our approach relies on the TCO‐cap developed in this study. We show that this TCO‐cap is compatible with in vitro transcription, including modified NTPs, and facilitates purification of long mRNAs via its hydrophobic effect. We are convinced that our results and methods will benefit the growing group of researchers studying fundamental aspects of mRNA and, in the long run, contribute to the development of click‐activatable mRNA‐based therapeutics with prospects in controlling their dosing and kinetics.

## Conflicts of Interest

The authors declare no conflicts of interest.

## Supporting information




**Supporting File 1**: The authors have cited additional references within the Supporting Information. [1–4]

## Data Availability

The data that support the findings of this study are available in the supplementary material of this article.
