# Supplementary material for: Small‐Molecule Activation of mRNA Translation by Click‐to‐Release Reaction in Cells
Source: Angew Chem Int Ed Engl. 2026 Mar 1;65(16):e24223. doi: 10.1002/anie.202524223 (PMC13080406; doi:10.1002/anie.202524223)
Supplement: Supplementary file 1 — Supporting File 1: The authors have cited additional references within the Supporting Information. [1–4] [file ANIE-65-e24223-s001.pdf]

Supplementary information

Small-molecule activation of mRNA translation by click-to-release reaction in cells

Table of contents

1. Materials 3
1.1 Chemicals 3
1.2 LC-solvents 4
1.3 Supplies for IVT and luminescence assay 4
1.4 Software 5
1.5 Buffers 5
2. Methods 6
3. Procedures 7
3.1 Click-to-release studies 7
3.2 Preparation of DNA templates for in vitro transcription (IVT) 8
3.3 In vitro transcription (IVT) 8
3.4 In vitro click-to-release of RNA analyzed by in vitro translation 9
3.5 Mammalian cell culture 10
3.6 In-cell luminescence assay of *Gaussia* luciferase (GLuc) 10
3.7 Microscopy 10
3.8 Evaluation of cell toxicity of the tetrazines using the MTT-assay 11
3.9 mRNA sequences used in this study 12
4. Synthesis procedures 15
4.1 Synthesis of guanosine 5′-diphosphate-imidazolide (GDP-Im) 15
4.2 Synthesis of *(Z)*-cyclooct-2-en-1-ol 16
4.3 Synthesis of *trans*-cyclooct-2-en-1-ol (TCO) 17
4.4 Synthesis of N^2-(2-*trans*-cyclooctyl)oxycarbonylguanosine (TCO-guanosine, **1**) 19
4.5 Synthesis of N^2-(2-*cis*-cyclooctyl)oxycarbonylguanosine (CCO-Guanosine) 20
4.6 Synthesis of N^2-(2-*trans*-cyclooctyl)oxycarbonylguanosine-5′-monophosphate (TCO-GMP, **2**) 22
4.7 Synthesis of N^2-(2-*cis*-cyclooctyl)oxycarbonylguanosine-5′-monophosphate (CCO-GMP) 23
4.8 Synthesis of N^2-(2-*trans*-cyclooctyl)oxycarbonyl-7-methylguanosine-5′-monophosphate (TCO-m^{7}GMP, **3**) 24
4.9 Synthesis of P1-(N^2-(2-*trans*-cyclooctyl)oxycarbonyl-7-methylguanosine-5′-yl)-P3-guanosin-5′-yl triphosphate (TCO-cap, **4**) 25
5. Supplementary figures 27
5.1 Binding of eIF4E to m^{7}GpppG 27
5.2 Irradiation apparatus 27
5.3 Click-to-release studies 28
5.4 Click-to-release on LC-MS 32

5.5 Cell lysate stability of the TCO-cap 33
5.6 Evaluation of TCO isomerization in cell culture medium 34
5.6.1 Isomerization in unsupplemented MEM 34
5.6.2 Isomerization in supplemented MEM 35
5.7 Evaluation of cell toxicity of the tetrazines using a MTT-assay 37
5.8 In cell click-to-release of *Renilla* luciferase RNA 38
5.9 In cell click-to-release of *Gaussia* luciferase mRNA using different experimental conditions 39
5.10 HPLC Purification of different mRNAs 40
5.11 Uncropped gel image 41
5.12 Microscopy images 42
5.13 HPLC analysis 54
5.14 HRMS spectra 56
5.15 NMR spectra 58
6. References 75

1. Materials

1.1 Chemicals

All utilised chemicals were purchased from Sigma Aldrich Chemie GmbH (Taufkirchen, GER), VWR International GmbH (Langenfeld, GER), TCI (Eschborn, GER), Carl Roth (Karlsruhe, GER), Acros Organics (Geel, BEL), AppliChem GmbH (Darmstadt, GER), Alfa Aesar (Ward Hill, MA, USA), Thermo Fisher Scientific (Waltham, MA, USA), Fisher Chemical (Hampton, NH, USA) or Fluorochem (Hadfield, UK) if not otherwise noted. Some compounds were also synthesized in this research group.

$AgNO_3$-coated silica gel was purchased from Merck (Darmstadt, GER) cat. no.: 21319-U.

All compounds were used immediately after purchase without further purification.

Solvents were evaporated at 40°C if not otherwise noted. If the compounds contained carbamate linkages, the bath temperature was reduced to 38°C to address the temperature sensitivity of these compounds.

1.2 LC-solvents

Solvents for HPLC and LC-MS were purchased from Fisher Chemical (Hampton, NH, USA), Biosolve Chimie SARL (Dieuze, FRA), VWR international GmbH (Langenfeld, GER) and Sigma Aldrich Chemie GmbH (Taufkirchen, GER).

1.3 Supplies for IVT and luminescence assay

G(5′)ppp(5′)A and m^{7}G(5′)ppp(5′)G cap analogues and all NTPs were purchased from Jena Bioscience (Jena, GER)). T7 RNA polymerase, RiboLock RNA inhibitor, pyrophosphatase and DNase I were purchased from Thermo Scientific. RNA Clean & Concentrator Kit was purchased from Zymo Research. 5′-Polyphosphatase was purchased from Biozym Scientific GmbH (Hessisch Oldendorf, GER), article no.: 136120. XRN1 exonuclease was bought from New England Biolabs, cat. no.: M0338L. For luminescence experiments with *Gaussia* or *Renilla* Luciferase the Gaussia-Juice Luciferase Assay kit from pjk was used.Devices

Table S1: Used devices.

Device	Supplier
1260 Infinity II LC system	Agilent Technologies, Inc., Santa Clara, CA, USA
Agilent LC/MSD iQ system	Agilent Technologies, Inc., Santa Clara, CA, USA
Agilent Ultivo triple quadrupole system	Agilent Technologies, Inc., Santa Clara, CA, USA
ELGA PURELAB® flex	ELGA LabWater, Celle, GER
Spark	TECAN Group AG, Männedorf, CHE
NanoQuantPlate™	TECAN Group AG, Männedorf, CHE
PrepChrom C-700 V5 HPLC system	Büchi Labortechnik AG, Flawil, CHE
puriFlash XS 520 Plus	Interchim S. A., Montlucon, FRA
Rayonet© RPR-200 photochemical reactor	The Southern New England Ultraviolet Co., Branford, CT, USA
Rayonet© RPR-2537A UV lamp	The Southern New England Ultraviolet Co., Branford, CT, USA
Series 1200 HPLC system	Agilent Technologies, Inc., Santa Clara, CA, USA
Ismatec MS-4 peristaltic pump	Ismatec Laborartoriumstechnik GmbH, Wertheim, GER

1.4 Software

Table S2: Used software.

Software	Supplier
ChemDraw Professional 21.0.0	PerkinElmer, Inc., Waltham, MA, USA
i-control^{TM}	TECAN Group AG, Männedorf, CHE
MestReNova x64 14.3.1	Mestrelab Research, Santiago de Compostela, ESP
Origin 2022b	OriginLab Corporation
ImageJ	NIH, Open Source

1.5 Buffers

Table S3: Used buffers.

Buffer	content
Ammoniumacetate buffer (50 mM, pH 6.0)	7.71 g NH_4OAc<br>1.9 L ddH_2O<br>adjust to pH 6 with acetic acid<br>fill up to 2 L total volume with ddH_2O<br>sterile filtration
K-Phosphate buffer (100 mM, pH 6.5)	27.2 g KH_2PO_4<br>17.4 g K_2HPO_4<br>3 L ddH_2O<br>sterile filtration
Lysis Buffer	Tris HCl 10 mM<br>NaCl 150 mM<br>EDTA 0.5 mM<br>NP40 0.1%<br>adjust to pH 7.5 with HCl
PBS 10x	80 g NaCl<br>2 g KCl<br>26.8 g $Na_2HPO_4 \cdot 7\ H_2O$<br>2.4 g KH_2PO_4<br>adjust to pH 7.4, fill to 1L with ddH_2O, autoclave
TBE 5x	108 g Tris<br>55 g boric acid<br>7.49 g EDTA<br>fill to 2L with ddH_2O and check pH 8.3
TEAB buffer (2 M)	555 mL triethylamine<br>1450 mL ddH_2O<br>bubbled with CO_2 overnight in ice-bath<br>adjusted to 2 L total volume with ddH_2O
TEAA buffer (2 M, pH 7.0)	229 mL acetic acid<br>1L ddH_2O<br>558 mL triethylamine was added dropwise<br>pH adjusted to 7 with acetic acid, filled up to 2L with ddH_2O

2. Methods

Analytical HPLC-measurements were performed on Agilent 1260 Infinity II systems with a Nucleodur® C18 Pyramid reversed-phase column from Macherey-Nagel (5 µm, 125 x 4 mm) or an InfinityLab Poroshell 120 EC-C18 3.0 x 50 mm, 2.7 µm column from Agilent Technologies and a diode array detector (DAD, 190 – 640 nm). For the elution a linear gradient of K-phosphate buffer (100 mM, pH 6.5) to 50% K-phosphate buffer/acetonitrile 1:1 or a linear gradient of ammoniumacetate buffer (50 mM, pH 6.0) to 50% acetonitrile was applied at a flow rate of 1 mL/min.

For preparative normal phase HPLC separations puriFlash® XS 520 Plus systems were used which were equipped with a multi-wavelength absorbance detector. Several columns were used including 40 g, 15 µm spherical silica gel (Interchim, PF-15SIHP-F0040) and 120 g, 50 µm spherical silica gel (Interchim, PF-50SIHP-F0120).

For preparative reversed phase HPLC separations a PrepChrom C-700 V5 HPLC system with a multi-wavelength absorbance detector was used. For the separations an aq-C18 column (Interchim, 150 g, pre-equilibrated with 0.1 M TEAB buffer) and an eluent of 0.1 M TEAB buffer/ACN with a gradient of 0 – 50 % ACN over 15 min at a flow rate of 65 mL/min was applied.

Liquid-chromatography-single-quadrupole-mass-spectrometry (LC-sQ MS) measurements were carried out on an Agilent LC/MSD iQ system with an ESI ion source. It contains a 1260 Infinity II LC unit with an Agilent Poroshell 120 EC-C18 column (2.7 µm, 3 x 50 mm) and an Agilent 1260 MWD detector. The gradient used on this device is shown in the following table, the flow rate was set to 0.8 mL/min.

Table S4: Gradient used on the LC-sQ MS system.

time /min	mobile phase
0:00	100% NH_4OAc buffer (50 mM, pH 6)
1:00	100% NH_4OAc buffer
6:00	50% NH_4OAc buffer, 50% acetonitrile
6:30	100% acetonitrile
7:30	100% acetonitrile
8:00	100% NH_4OAc
9:00	100% NH_4OAc

Liquid-chromatography-triple-quadrupole-mass-spectrometry (LC-QqQ MS) measurements were performed using an Agilent 1260 Infinity II LC system with an Agilent Poroshell 120 EC-C18 column (2.7 µm, 3 x 50 mm) and a dual-wavelength absorbance detector G7114A connected to an Agilent Ultivo mass spectrometer with JetStream ion source. For the separation a gradient of NH_4OAc (20 mM, pH 6) and acetonitrile with 0 – 50% of acetonitrile at a flow rate of 0.8 mL/min was chosen.

High resolution mass spectrometry measurements were either carried out on a LTQ Orbitrap XL from Thermo Fisher Scientific with electrospray ionisation or on a Orbitrap Velos Pro from Thermo Fisher Scientific also using electrospray ionisation. Also, an Exploris 120 Electrospray Obitrap from Thermo Fisher Scientific and an ISQ 7000 GC-MS from Thermo Fisher Scientific with EI ionisation were used.

NMR measurements including ^1H, ^{13}C and ^{31}P were carried out on Agilent DD2 600 (599, 151 and 243 MHz respectively), Agilent DD2 500 (500, 126, 202 MHz respectively) and Bruker NEO 400 (400, 101, 162 MHz respectively) devices. The compounds were dissolved in deuterated solvents such as DMSO-d_6, $CDCl_3$ and D_2O depending on the solubility of the compounds. 2D-NMR measurements were performed as well including ^1H-COSY, ^1H-^{13}C-HSQC and ^1H-^{13}C-HMBC experiments. The peaks in the 1D-data were assigned according to correlations in the 2D-spectra. All chemical shifts are reported in parts per million (ppm) and coupling constants in Hertz (Hz).

3. Procedures

3.1 Click-to-release studies

The results of the click-to-release reactions of the TCO-cap and tetrazines **5** to **7** are shown in Figures S3-S9. Tetrazines **5** to **7** were tested in the following reaction design and the amounts of click and release products were monitored after certain timepoints on HPLC using an external calibration. The samples contained 0.06 mM TCO-cap, 0.4 mM trihydroxyphenylcoumarine (THPC) as internal standard and 0.2 mM tetrazine (tetrazine **6**, **5** or **7**). The samples were filled to 25 µL reaction volume with sodium phosphate buffer (0.2 M, pH 7.2) or MEM medium and the general injection volume on HPLC was 2 µL. All ingredients were placed in a reaction tube and mixed by vortexing before incubating the reactions at 37°C.

3.2 Preparation of DNA templates for in vitro transcription (IVT)

Linearized plasmids were used as DNA template for run-off transcription. To prepare the DNA templates, purified plasmid were cut after the poly-A-tail. The restriction enzyme used depended on the vector that the plasmids were in. For eGFP and mScarlet DNA template, 5 µg of purified plasmid was incubated with 12 µL rCutsmartbuffer (1x, New England Biolabs), 3 µL PaqCI (NEB) and 3 µL activator (NEB) in 120 µL ddH_2O for 1h at 37°C followed by heat denaturation of the enzyme for 10 minutes at 65°C. For *Renilla* luciferase and *Gaussia* luciferase DNA templates, 5 µg of purified plasmid was incubated with 10 µL FD buffer (1x, Thermofisher) and 5 µL FastDigest PacI (Thermofisher) for 15 minutes at 37°C, followed by denaturation of the enzyme for 10 minutes at 65°C. Afterwards the ends of all linearized plasmids were dephosphorylated by adding 5 µL FastDigest (Thermofisher) and incubating for 15 minutes at 37°C followed by denaturation for 5 minutes at 75°C. The DNA templates were purifies using the NucleoSpin Gel and PCR Clean-up kit (Macherey–Nagel) according to the kits instructions. The purity of the DNA templates was evaluated on a 1% agarose gel.

3.3 In vitro transcription (IVT)

RNA was prepared via in vitro transcription using T7 RNA polymerase in a 50 µL reaction mixture that contains DNA template (400 ng), transcription buffer (1x, Thermo Scientific), GTP (0.25 mM), A/C/UTP (0.5 mM) or A/m^5C/m^1Ψ (0.5 mM), cap analogue (1 mM), Ribolock RNase inhibitor (60 U, Thermo Scientific), pyrophosphatase (0.2 U, Thermo Scientific) and T7-polymerase (100 U, Thermo Scientific). The IVT mixture was incubated for 2 hours at 37°C before DNase I (4 µL, 4 U, Thermo Scientific) was added. After further incubation of 60 min at 37 °C, the samples were purified over purification columns (RNA Clean & Concentrator Kit, Zymo Research) following the kits instructions. The mRNA was eluted in ddH_2O (36 µL) and the concentration was determined using the Tecan Spark platereader. In the next step, the uncapped mRNA was digested.

For cap 0- and ApppG-capped mRNA, a maximum of 5 µg mRNA was treated with 5′-Polyphosphatase (0.5 µL, 10 U, Biozym Scientific) in 1x reaction buffer (20 µL) for 1 hour at 37°C. $MgCl_2$ (2.5 µL, 5.3 mM final conc.) and XRN-1 (1 µL, 1 U) were added per 5 µg of RNA, before further incubation for 1 hour at 37°C. The reaction mixtures

were purified on purification columns (RNA Clean & Concentrator Kit, Zymo Research) following the kits instructions. The concentration of the RNA was determined using the Tecan Spark platereader before final purification of all mRNAs (cap 0- TCO- and ApppG-capped mRNAs) on a Series 1200 HPLC (Agilent Technologies) equipped with a YMC-Triart Bio C4 (YMC) column to obtain pure capped mRNA (Buffer A: 0.5 M TEAA (pH 7.0), 5% ACN B: 100% ACN; linear gradient of 4-10% from buffer B in Buffer A over 28 min at 1mL/min flow rate and 50 °C column temperature). The pure fractions were precipitated using 0.3M NaOAc and 0.12 µg/µL glycogen (Thermofisher), and 1.3V (650 µL) cold isopropanol. The samples were incubated at -20°C for at least one night, and afterwards centrifuged at 21300 rcf, 4°C for at least 1h. The supernatant was removed and the pellet was washed with cold 70% EtOH. Afterwards the samples were centrifuged at 21300 rcf, 4°C for at least 30 minutes. The supernatant was removed and the pellets were dried by incubating the Eppendorf tubes with open lid for 10 minutes at 37°C. The pellets were resolubilized in 20 µL ddH_2O and the concentrations was determined the Tecan Spark platereader. The purity was evaluated on a 7.5 % denaturing polyacrylamide minigel (Rluc/Gluc) or a denaturing 1% agarose gel (eGFP/mScarlet).

3.4 In vitro click-to-release of RNA analyzed by in vitro translation

150-650 ng *Renilla* luciferase RNA was incubated together with either $h2P_2$ (7.5 equivalents) or the same volume DMSO in 20 µL Na-phosphate buffer (0.1 M, pH 7.2) for 1 h at 37°C. Afterwards, the RNA was purified over purification columns (RNA Clean & Concentrator Kit, Zymo Research) following the kits instructions. The in vitro translation mix was prepared by adding together per reaction 40 ng of RNA, High Salt mix (1x, Invitrogen), L-Methionine (0.05 mM), Ribolock RNase inhibitor (5.7 U, Thermo Scientific) and 8.5 µL retic lysate (Invitrogen) in a 13.5 µL reaction and incubated for 1.5 h at 30°C. Afterwards the reaction mixture was transferred onto a 96-well plate (triplicates, 2 µL per well). In a Tecan Spark platereader, 50 µL *Gaussia* luciferase reaction mixture (prepared according to the manufacturer's instructions, pjk) was added per well and the luminescence was measured.

3.5 Mammalian cell culture

HeLa cells (Merck) were cultured in HeLa medium: MEM Earle′s media (Sigma-Aldrich) supplemented with L-glutaMAX (2 mM, Gibco), MEM non-essential amino acids (1x, Sigma-Aldrich) and fetal calf serum (10%, PAN) under 5% CO_2 and 37°C.

3.6 In-cell luminescence assay of *Gaussia* luciferase (GLuc)

HeLa cells were seeded either one (30.000 cells/well) or two (15.000 cells/well) days prior to transfection in a 96-well plate. Directly before the experiment the medium was exchanged to fresh HeLa medium (100 µL). For each well, 0.15 µL Lipofectamine messenger max (Thermofisher) was added to 6.1 µL Opti-MEM (Gibco), vortexed for three seconds and incubated at RT for 10 minutes. Afterwards 100 ng of RNA in 6.25 µL OptiMEM was added and the samples were incubated at RT for 5 minutes, before addition of 12.5 µL to each well. The cells were incubated for 4 h at 37 °C before the medium was exchanged to either HeLa medium containing 0.1% DMSO or HeLa medium containing 10 µM tetrazine. After 1 h of incubation at 37 °C, the medium for all cells was exchanged to Hela medium and the cells were allowed to grow for another 19 h. 24 hours post transfection the cell supernatant was transferred, 100x diluted with PBS, and pipetted onto a 96-well plate (triplicates, 5 µL per well). In a Tecan Spark platereader, 50 µL *Gaussia* luciferase reaction mixture (prepared according to the manufacturer’s instructions, pjk) was added per well and the luminescence was measured.

3.7 Microscopy

HeLa cells were seeded two days prior to transfection in a 96-well plate with a glass bottom suitable for microscopy (15.000 cells/well, Greiner Bio-One). Directly before the experiment the medium was exchanged to fresh HeLa medium (100 µL) . For each well, 0.15 µL Lipofectamine messenger max (Thermofisher) was added to 6.1 µL Opti-MEM (Gibco), vortexed for three seconds and incubated at RT for 10 minutes. Afterwards 100 ng total mRNA (80 ng differently capped eGFP-mRNA and 20 ng cap 0-mScarlet-mRNA) in 6.25 µL OptiMEM was added and the samples were incubated at RT for 5 minutes, before addition of 12.5 µL to each well. The cells were incubated for 4 h at 37 °C before the medium was exchanged to either HeLa medium containing

0.1% DMSO or HeLa medium containing 10 μM tetrazine. After 1 h of incubation at 37 °C, the medium for all cells was exchanged to Hela medium and the cells were allowed to grow for another 19 h. 24 hours post transfection the cells were washed twice with PBS, and fixed by incubation with 50 μL paraformaldehyde (4% in PBS) for 10 minutes at RT. The cells were washed twice with PBS and stained with 50 μL 4′,6-diamidino-2-phenylindole (DAPI, 10 μg/mL in PBS). The cells were washed twice with PBS and were stored in PBS in the dark until imaging. The cells on the 96-well plate were imaged using a Leica SP8 inverted confocal laser scanning microscope equipped with a 10x (air) and a 40x (oil immersion) objective. Images were taken for transmitted light, DAPI, GFP and mScarlet channels and analysed with ImageJ.

3.8 Evaluation of cell toxicity of the tetrazines using the MTT-assay

HeLa cells were seeded two days prior to transfection in a 96-well plate (15.000 cells/well). Directly before the experiment the medium was exchanged to fresh HeLa medium. For each well, 0.15 μL Lipofectamine messenger max (Thermofisher) was added to 6.1 μL Opti-MEM (Gibco), vortexed for three seconds and incubated at RT for 10 minutes. Afterwards 100 ng of TCO-capped Gluc mRNA in 6.25 μL OptiMEM was added and the samples were incubated at RT for 5 minutes, before addition of 12.5 μL to each well. The cells were incubated for 4 h at 37 °C before the medium was exchanged to either HeLa medium, HeLa medium containing 0.1% DMSO or HeLa medium containing 10 μM tetrazine. After 1 h of incubation at 37°C, the medium for all cells was exchanged to HeLa medium and the cells were allowed to grow for another 19h. Afterwards 12.5 μL 3-(4,5-Dimethyl-2-thiazolyl)-2,5-diphenyl-2H-tetrazolium bromide (MTT, 12 mM in PBS) was added to each well. After 4h of incubation at 37°C, the supernatant was removed and 125 μL HCl in isopropanol (0.04 M) was added to each well and incubated for 1 h at RT. For each well, 100 μL of supernatant was transferred to a fresh well and the absorption was measured on the Tecan Spark plate reader at 550 nm.

3.9 mRNA sequences used in this study

RNA	Sequence
**eGFP mRNA**	5'-<br>GGGAGAGATATCACGCGTTCTAGAGCTAGCGCTACCGGACTCAGAT<br>CTCGAGCTCAAGCTTCGAATTCTGCAGTCGACGGTACCGCGGGCC<br>CGGGATCCACCGGTCGCCACCATGGTGAGCAAGGGCGAGGAGCT<br>GTTCACCGGGGTGGTGCCCATCCTGGTCGAGCTGGACGGCGACGT<br>AAACGGCCACAAGTTCAGCGTGTCCGGCGAGGGCGAGGGCGATG<br>CCACCTACGGCAAGCTGACCCTGAAGTTCATCTGCACCACCGGCAA<br>GCTGCCCGTGCCCTGGCCCACCCTCGTGACCACCCTGACCTACGG<br>CGTGCAGTGCTTCAGCCGCTACCCCGACCACATGAAGCAGCACGA<br>CTTCTTCAAGTCCGCCATGCCCGAAGGCTACGTCCAGGAGCGCAC<br>CATCTTCTTCAAGGACGACGGCAACTACAAGACCCGCGCCGAGGT<br>GAAGTTCGAGGGCGACACCCTGGTGAACCGCATCGAGCTGAAGGG<br>CATCGACTTCAAGGAGGACGGCAACATCCTGGGGCACAAGCTGGA<br>GTACAACTACAACAGCCACAACGTCTATATCATGGCCGACAAGCAGA<br>AGAACGGCATCAAGGTGAACTTCAAGATCCGCCACAACATCGAGGA<br>CGGCAGCGTGCAGCTCGCCGACCACTACCAGCAGAACACCCCCAT<br>CGGCGACGGCCCCGTGCTGCTGCCCGACAACCACTACCTGAGCAC<br>CCAGTCCGCCCTGAGCAAAGACCCCAACGAGAAGCGCGATCACAT<br>GGTCCTGCTGGAGTTCGTGACCGCCGCCGGGATCACTCTCGGCAT<br>GGACGAGCTGTACAAGTAAAGCGGCCGCAGCTCGCTTTCTTGCTGT<br>CCAATTTCTATTAAAGGTTCCTTTGTTCCCTAAGTCCAACTACTAAAC<br>TGGGGGATATTATGAAGGGCCTTGAGCATTTGGATTCTGCCTAATAA<br>AAAACATTTATTTTCATTGCGTTTAGCTCGCTTTCTTGCTGTCCAATT<br>TCTATTAAAGGTTCCTTTGTTCCCTAAGTCCAACTACTAAACTGGGG<br>GATATTATGAAGGGCCTTGAGCATTTGGATTCTGCCTAATAAAAAACA<br>TTTATTTTCATTGAAAAAAAAAAAAAAAAAAAAAAAAAAAAAAAAAAAAAAAAA<br>AAAAAAAAAAAAAAAAAAAAAAAAAAAAAAAAAAAAAAAAAAAAAAAAAAAAAA<br>AAAAAAAAAAAAAAAAAAAAAAAAAAAAAAAAAAAAAAAAAAAAAAAAAA-3'
**mScarlet mRNA**	5'-<br>GGGAGAGAUAUCACGCGUUCUAGAGCUAGCGCUACCGGACUCAG<br>AUCUCGAGCUCAAGCUUCGAAUUCUGCAGUCGACGGUACCGCGG<br>GCCCGGGAUCCACCUCGCCACCAUGAGUAAAGGAGAAGCUGUGA<br>UUAAAGAGUUCAUGCGCUUCAAAGUUCACAUGGAGGGUUCUAUG<br>AACGGUCACGAGUUCGAGAUCGAAGGCGAAGGCGAGGGCCGUCC<br>GUAUGAAGGCACCCAGACCGCCAAACUGAAAGUGACUAAAGGCG<br>GCCCGCUGCCUUUUUCCUGGGACAUCCUGAGCCCGCAAUUUAUG<br>UACGGUUCUAGGGCGUUCAUCAAACACCCAGCGGAUAUCCCGGA<br>CUAUUAUAAGCAGUCUUUUCCGGAAGGUUUCAAGUGGGAACGCG<br>UAAUGAAUUUUGAAGAUGGUGGUGCCGUGACCGUCACUCAGGAC<br>ACCUCCCUGGAGGAUGGCACCCUGAUCUAUAAAGUUAAACUGCG<br>UGGUACUAAUUUUCCACCUGAUGGCCCGGUGAUGCAGAAAAAGA<br>CGAUGGGUUGGGAGGCGUCUACCGAACGCUUGUAUCCGGAAGA<br>UGGUGUGCUGAAAGGCGACAUUAAAAUGGCCCUGCGCCUGAAAG<br>AUGGUGGCCGCUAUCUGGCUGACUUCAAAACCACGUACAAAGCC<br>AAGAAACCUGUGCAGAUGCCUGGCGCGUACAAUGUGGACCGCAA<br>ACUGGACAUCACCUCUCAUAAUGAAGAUUAUACGGUGGUAGAGC

	AAUAUGAGCGCUCCGAGGGUCGUCAUUCUACCGGUGGCAUGGAU GAACUAUACAAAUAAAGCGGCCGCAGCUCGCUUUCUUGCUGUCC AAUUUCUAUUAAAGGUUCCUUUGUUCCCUAAGUCCAACUACUAAA CUGGGGGAUAUUAUGAAGGGCCUUGAGCAUUUGGAUUCUGCCUA AUAAAAAACAUUUAUUUUCAUUGCGUUUAGCUCGCUUUCUUGCU GUCCAAUUUCUAUUAAAGGUUCCUUUGUUCCCUAAGUCCAACUA CUAAACUGGGGGAUAUUAUGAAGGGCCUUGAGCAUUUGGAUUCU GCCUAAUAAAAAACAUUUAUUUUCAUUGAAAAAAAAAAAAAAAAAA AAAAAAAAAAAAAAAAAAAAAAAAAAAAAAAAAAAAAAAAAAAAAAAA AAAAAAAAAAAAAAAAAAAAAAAAAAAAAAAAAAAAAAAAAAAAAAAA AAAAAAAAAAAAAA-3'
**GLuc mRNA**	5'- GGGAAAUAAGAGAGAAAAGAAGAGUAAGAAGAAAUAUAAGAGAAU UCGCCACCAUGGGAGUCAAAGUUCUGUUUGCCCUGAUCUGCAUC GCUGUGGCCGAGGCCAAGCCCACCGAGAACAACGAAGACUUCAA CAUCGUGGCCGUGGCCAGCAACUUCGCGACCACGGAUCUCGAUG CUGACCGCGGGAAGUUGCCCGGCAAGAAGCUGCCGCUGGAGGU GCUCAAAGAGAUGGAAGCCAAUGCCCGGAAAGCUGGCUGCACCA GGGGCUGUCUGAUCUGCCUGUCCCACAUCAAGUGCACGCCCAAG AUGAAGAAGUUCAUCCCAGGACGCUGCCACACCUACGAAGGCGA CAAAGAGUCCGCACAGGGCGGCAUAGGCGAGGCGAUCGUCGACA UUCCUGAGAUUCCUGGGUUCAAGGACUUGGAGCCCAUGGAGCAG UUCAUCGCACAGGUCGAUCUGUGUGUGGACUGCACAACUGGCUG CCUCAAAGGGCUUGCCAACGUGCAGUGUUCUGACCUGCUCAAGA AGUGGCUGCCGCAACGCUGUGCGACCUUUGCCAGCAAGAUCCAG GGCCAGGUGGACAAGAUCAAGGGGGCCGGUGGUGACUAAGGAU CCGCUGCCUUCUGCGGGGCUUGCCUUCUGGCCAUGCCCUUCUU CUCUCCCUUGCACCUGUACCUCUUGGUCUUUGAAUAAAGCCUGA GUAGGAAGAAUAUUAAAAAAAAAAAAAAAAAAAAAAAAAAAAAAAA AAAAAAAAAAAA-3'
**RLuc mRNA**	5'- GGGAAAUAAGAGAGAAAAGAAGAGUAAGAAGAAAUAUAAGAGAAU UCGCCACCAUGACUUCGAAAGUUUAUGAUCCAGAACAAAGGAAAC GGAUGAUAACUGGUCCGCAGUGGUGGGCCAGAUGUAAACAAAUG AAUGUUCUUGAUUCAUUUAUUAAUUAUUAUGAUUCAGAAAAACAU GCAGAAAAUGCUGUUAUUUUUUUACAUGGUAACGCGGCCUCUUC UUAUUUAUGGCGACAUGUUGUGCCACAUAUUGAGCCAGUAGCGC GGUGUAUUAUACCAGACCUUAUUGGUAUGGGCAAAUCAGGCAAA UCUGGUAAUGGUUCUUAUAGGUUACUUGAUCAUUACAAAUAUCU UACUGCAUGGUUUGAACUUCUUAAUUUACCAAAGAAGAUCAUUUU UGUCGGCCAUGAUUGGGGUGCUUGUUUGGCAUUUCAUUAUAGC UAUGAGCAUCAAGAUAAGAUCAAAGCAAUAGUUCACGCUGAAAGU GUAGUAGAUGUGAUUGAAUCAUGGGAUGAAUGGCCUGAUAUUGA AGAAGAUAUUGCGUUGAUCAAAUCUGAAGAAGGAGAAAAAAUGGU UUUGGAGAAUAACUUCUUCGUGGAAACCAUGUUGCCAUCAAAAAU CAUGAGAAAGUUAGAACCAGAAGAAUUUGCAGCAUAUCUUGAACC AUUCAAAGAGAAAGGUGAAGUUCGUCGUCCAACAUUAUCAUGGC CUCGUGAAAUCCCGUUAGUAAAAGGUGGUAAACCUGACGUUGUA CAAAUUGUUAGGAAUUAUAAUGCUUAUCUACGUGCAAGUGAUGA

	UUUACCAAAAAUGUUUAUUGAAUCGGACCCAGGAUUCUUUUCCAA<br>UGCUAUUGUUGAAGGUGCCAAGAAGUUUCCUAAUACUGAAUUUG<br>UCAAAGUAAAAGGUCUUCAUUUUUCGCAAGAAGAUGCACCUGAU<br>GAAAUGGGAAAAUAUAUCAAAUCGUUCGUUGAGCGAGUUCUCAAA<br>AAUGAACAAUAAGGAUCCGCUGCCUUCUGCGGGGCUUGCCUUCU<br>GGCCAUGCCCUUCUUCUCUCCCUUGCACCUGUACCUCUUGGUCU<br>UUGAAUAAAGCCUGAGUAGGAAGAAUAUUAAAAAAAAAAAAAAAAA<br>AAAAAAAAAAAAAAAAAAAAAAAAAAAAA-3'

4. Synthesis procedures

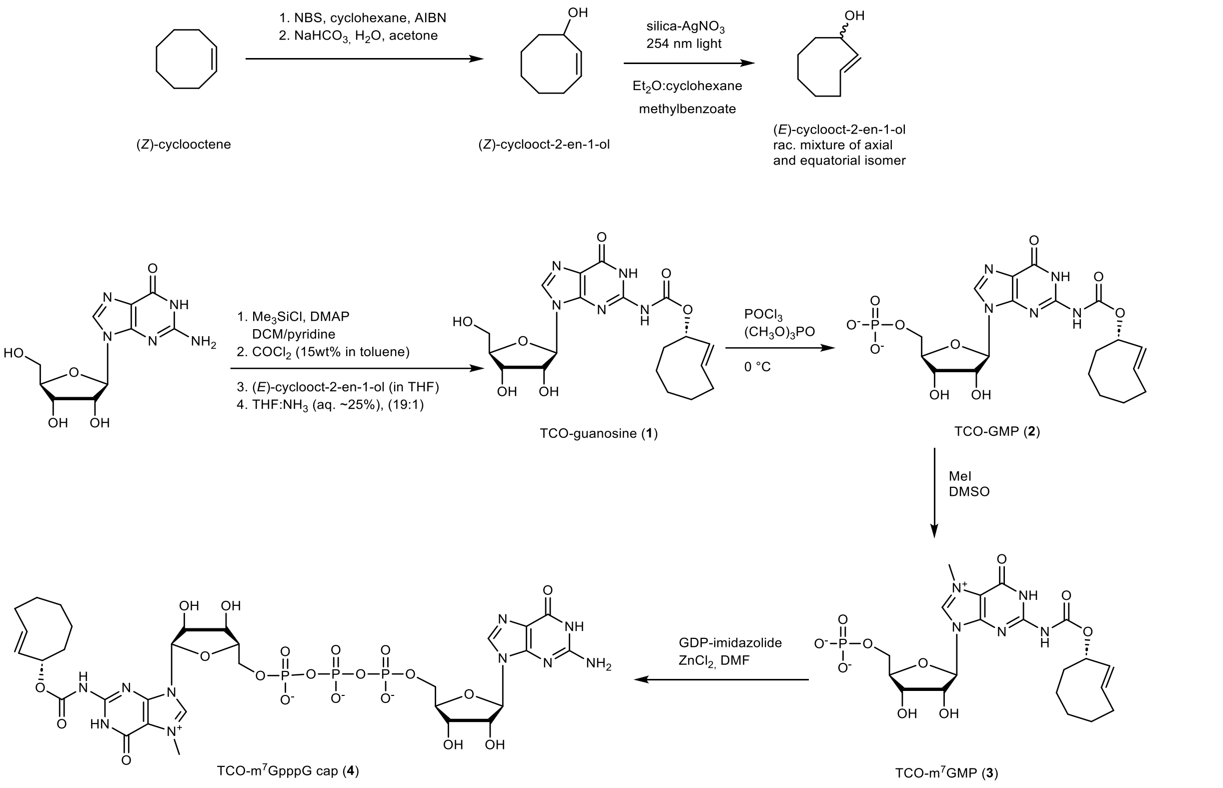

Figure S1: Synthesis of the TCO-cap (**4**).

4.1 Synthesis of guanosine 5′-diphosphate-imidazolide (GDP-Im)

GDP-Imidazolide was synthesised from commercially available guanosine 5′-diphosphate (Biosynth-Carbosynth, product number NG08963, cas: 43139-22-6) according to a procedure by Jemielity *et al.*[1] Briefly, the guanosine 5′-diphosphate Na^+ salt (1.001 g, 1.34 mmol, 1 eq.) was changed into the triethylammonium (TEA) salt on Dowex 50WX8 resin (16.4 g) and then mixed with imidazole (459 mg, 6.74 mmol, 5 eq.) and 2,2′- dithiodipyridine (592 mg, 2.69 mmol, 2 eq.) in anhydrous DMF (13.5 mL) and TEA (187 µL). Triphenylphosphine (705 mg, 2.69 mmol, 2 eq.) was added, and the mixture was stirred overnight at room temperature. The mixture was poured in anhydrous sodium perchlorate (1.08 g) dissolved in dry acetone (54 mL). After cooling at 4 °C for 2 hours, the precipitate was filtered, washed with cold dry acetone (6 x 13.5 mL), and lyophilised (yield: 965.3 mg, quantitative).

HRMS (ESI-): m/z expected: 245.5183 for $[M-2H]^{2-}$, found: 245.5184.

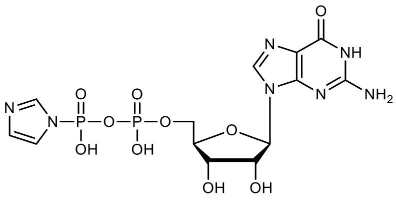

Exact Mass: 493,05121

4.2 Synthesis of *(Z)*-cyclooct-2-en-1-ol

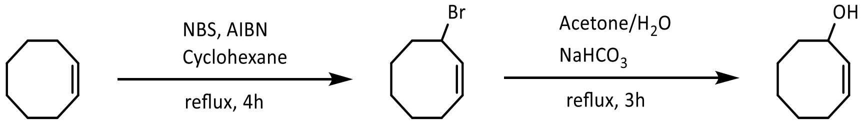

(*Z*)-cyclooct-2-en-1-ol was synthesised following the procedure from de Geus *et al.*[2] *N*-bromsuccinimide (30 g, 168.6 mmol, 1.00 eq.) and azobisisobutyronitrile (19.7 mg, 0.12 mmol, 0.07 mol%) were dissolved in cyclohexane (120 mL) under argon atmosphere. *Z*-cyclooctene (30 mL, 231 mmol, 1.37 eq.) was added and the reaction mixture was refluxed under argon for 4 hours at 90 °C. After that, the mixture was allowed to reach room temperature and the precipitate was removed by filtration after cooling to 0°C. The reaction mixture was concentrated *in vacuo* to obtain 3-bromocyclooctene as the crude reaction product. Without further purification, 3-bromocyclooctene was dissolved in acetone (180 mL) and water (90 mL) and $NaHCO_3$ (20 g, 238.5 mmol, 2.00 eq.) was added. The mixture was stirred under reflux for 3h at 75 °C. After cooling to room temperature, excess of $NaHCO_3$ was removed by filtration and the filtrate was extracted with diethylether (3 x 150 mL). The organic part was dried over $MgSO_4$, filtered and concentrated under reduced pressure to give (*Z*)-cyclooct-2-en-1-ol as a yellow oil (17.04 g, 135.2 mmol, 80%).

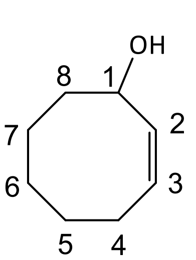

^1H NMR (500 MHz, DMSO-d_6) δ = 5.46 (m, 2H, H-2, H-3), 4.60 (s, 1H, OH), 4.40 (m, 1H, H-1), 2.13 (m, 1H, H-4a), 2.01 (m, 1H, H-4b), 1.77 (m, 1H, H-8a), 1.59 (m, 1H; H-5a), 1.46 (m, 2H, H-6), 1.44 (m, 2H, H-7), 1.30 (m, 2H, H-5b, H-8b) ppm.

^{13}C NMR (126 MHz, DMSO-d_6) δ = 137.46 (C-2), 126.72 (C-3), 67.91 (C-1), 39.26 (C-8), 29.20 (C-5), 26.10 (C-4), 26.03 (C-6), 23.79 (C-7) ppm.

HRMS (EI): m/z calculated for $C_8H_{14}O$: 126.10 $[M]^+$; found: 126.12.

4.3 Synthesis of *trans*-cyclooct-2-en-1-ol (TCO)

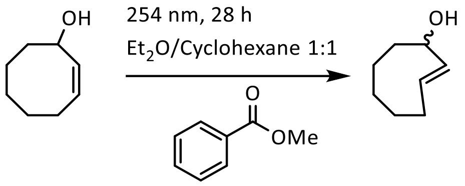

The synthesis was carried out according to Darko *et al.*[3] (*Z*)-cyclooct-2-en-1-ol (606, mg, 4.8 mmol, 1 eq.) was dissolved in diethylether/cyclohexane 1:1 (200 mL) and methylbenzoate (1.34 g, 9.60,0 mmol, 2 eq.) was added. The solution was irradiated for 28 h at 254 nm while being continuously passed through a column cartridge loaded with $AgNO_3$-coated silica (10 wt% $AgNO_3$, 8.2 g, 1.0 eq.) using an Ismatec MS-4 peristaltic pump and corresponding Ismatec Solva PVC pump tubes (2.79 mm ID). After the isomerization reaction remaining (*Z*)-cyclooct-2-en-1-ol was eluted from the cartridge with dichloromethane (DCM) and the cartridge was dried with air. Then, the silica was stirred in NH_4OH/DCM (1:1, 100 mL) for 10 min and filtered off. The aqueous part was extracted with DCM (50 mL) and the combined organic parts were washed with water (2 x 25 mL) and then dried over $MgSO_4$, filtered and concentrated. The crude product was obtained as a yellow oil. The crude product was purified using a puriFlash system with a PF15-SIHP F0040 column (gradient: 0 – 60% diethylether in cyclohexane). The axial isomer eluted first followed by the equatorial isomer. Fractions containing pure isomers were combined and concentrated to yield both isomers as yellow oils (axial isomer: 149 mg, 1.2 mmol, 25%; equatorial isomer: 120 mg, 1.0 mmol, 20%).

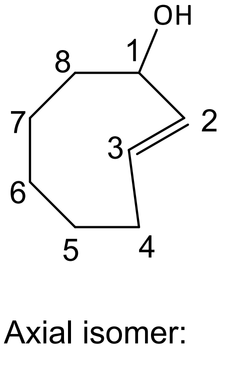

Axial isomer:

^{1}H NMR (599 MHz, DMSO-d_6) δ = 5.82 (dddd, J = 16.2, 11.3, 3.8, 1.1 Hz, 1H, H-3), 5.52 (dd, J = 16.3, 2.4 Hz, 1H, H-2), 4.56 (s, 1H, OH), 4.39 (dq, J = 3.8, 2.0 Hz, 1H, H-1), 2.36 (m, 1H, H-4a), 1.92 (qd, J = 11.4, 4.4 Hz, 1H, H-4b), 1.87 (m, 1H, H-5a), 1.78 (m, 1H, H-8a), 1.75 (m, 1H, H-6a), 1.54 (dddd, J = 14.1, 12.5, 3.1, 1.6 Hz, 1H, H-8b), 1.45 (dddt, J = 15.1, 11.5, 5.6, 1.9 Hz, 1H, H-7a), 1.39 (dtdd, J = 13.8, 12.3, 4.5, 1.4 Hz, 1H, H-5b), 1.06 (dddt, J = 14.5, 12.6, 6.1, 1.6 Hz, 1H, H-7b), 0.74 (dddt, J = 14.3, 12.3, 6.0, 1.6 Hz, 1H, H-6b) ppm.

^{13}C NMR (151 MHz, DMSO-d_6) δ = 136.47 (C-2), 129.09 (C-3), 69.57 (C-1), 43.18 (C-8), 35.62 (C-5), 35.25 (C-4), 28.75 (C-6), 22.82 (C-7) ppm.

HRMS (EI): m/z calculated for $C_8H_{14}O$: 126.10 $[M]^+$; found: 126.13.

Equatorial isomer:

^{1}H NMR (599 MHz, DMSO-d_6) δ = 5.49 (m, 1H, H-3), 5.45 (m, 1H, H-2), 4.80 (d, J = 3.8 Hz, 1H, OH), 4.03 (tdd, J = 9.4, 5.6, 3.8 Hz, 1H, H-1), 2.31 (m, 1H, H-4a), 1.97 (m, 1H, H-8a), 1.87 (m, 2H, H-3b, H-5a), 1.76 (dddt, J = 14.8, 11.1, 5.7, 1.6 Hz, 1H, H-6a), 1.65 (dddt, J = 14.9, 11.3, 5.8, 1.7 Hz, 1H, H-7a), 1.33 (m, 2H, H-4b, H-8b), 0.81 (m, 1H, H-6b), 0.70 (dddd, J = 14.9, 12.9, 4.6, 3.0 Hz, 1H, H-7b) ppm.

^{13}C NMR (151 MHz, DMSO-d_6) δ = 137.39 (C-2), 129.35 (C-3), 75.43 (C-1), 44.54 (C-8), 35.27 (C-5), 34.82 (C-4), 28.74 (C-6), 27.10 (C-7) ppm.

HRMS (EI): m/z calculated for $C_8H_{14}O$: 126.10 $[M]^+$; found: 126.15.

4.4 Synthesis of N^2-(2-*trans*-cyclooctyl)oxycarbonylguanosine (TCO-guanosine, **1**)

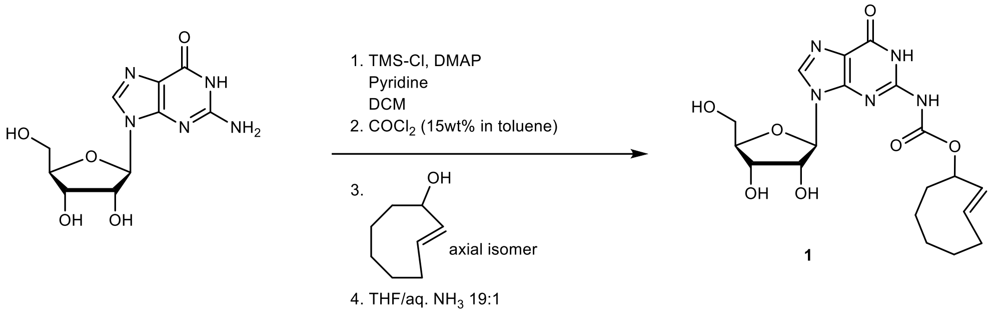

Guanosine (582 mg, 2.05 mmol, 1.0 eq.) and DMAP (27 mg, 0.23 mmol, 0.1 eq.) were suspended in dry DCM (41 mL) under argon atmosphere. The mixture was cooled to 0 °C and dry pyridine (6.8 mL) was added. Trimethylsilylchloride (1.7 mL, 12.94 mmol, 6.3 eq.) was added dropwise and the reaction was stirred for 2 hours. Next, phosgene (2.1 mL, 15 wt% solution in toluene, 2.9 mmol, 1.4 eq.) was added dropwise at 0 °C giving a yellow precipitate. The reaction was stirred for 30 min. Lastly, axial *trans*-cyclooct-2-en-1-ol (985 mg, 7.8 mmol, 3.8 eq.) dissolved in dry THF (36 mL) was added and the reaction was stirred overnight while warming up to room temperature. Then, chloroform (68 mL) and water (68 mL) were added and the organic layer was washed with water (3 x 68 mL) and concentrated under reduced pressure. The obtained brown oily solid was dissolved in THF (34 mL), aqueous ammonia (1.7 mL, 25% solution) was added and the solution was stirred vigorously overnight. The obtained precipitate was centrifuged (3220 rcf, 4 °C, 10 min), the supernatant was removed and the precipitate was purified on a puriFlash system (PF50-SIHP F0120 column; solvent gradient: 100% DCM for 3.5 min, change to 2% MeOH/DCM over 3.5 min, change to 30% MeOH/DCM over 10.5 min, change to 100% MeOH over 2 min). Fractions containing the product were combined and concentrated. The obtained product was washed with pentane to remove traces of solvents and dissolved in dioxane for lyophilisation. After lyophilisation compound (**1**) was obtained as an off-white solid (270 mg, 0.62 mmol, 30%).

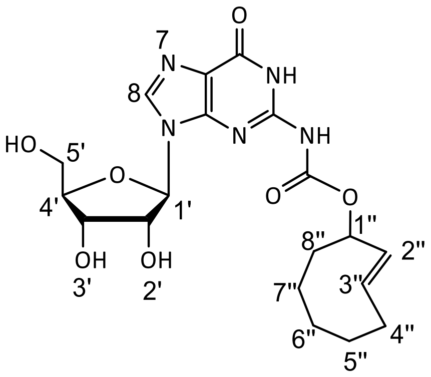

^{1}H NMR (599 MHz, DMSO-d_6) δ = 8.22 (d, J = 1.9 Hz, 1H, H-8), 5.87 (m, 1H, H-3″), 5.78 (dd, J = 5.8, 2.1 Hz, 1H, H-1′), 5.62 (dd, J = 16.4, 2.3 Hz, 1H, H-2″), 5.44 (d, J = 5.8 Hz, 1H, 2′OH), 5.39 (s, 1H, H-1″), 5.14 (d, J = 4.7 Hz, 1H, 3′OH), 4.99 (m, 1H, 5′OH), 4.47 (dq, J = 10.7, 5.5 Hz, 1H, H-2′), 4.14 (d, J = 4.6 Hz, 1H, H-3′), 3.90 (q, J = 4.2 Hz, 1H, H-4′), 3.64 (dt, J = 11.8, 4.9 Hz, 1H, H-5′a), 3.54 (m, 1H, H-5′b), 2.43 (m, 1H, H-4″a), 2.03 (m, 1H, H-8″a), 1.99 (m, 1H, H-4″b), 1.94 (m, 1H, H-5″a), 1.82 (m, 1H, H-6″a), 1.76 (m, 1H, H-8″b), 1.62 (t, J = 12.3 Hz, 1H, H-7″a), 1.44 (m, 1H, H-5″b), 1.08 (m, 1H, H-7″b), 0.84 (m, 1H, H-6″b) ppm.

^{13}C NMR (151 MHz, DMSO-d_6) δ = 155.51, 149.60, 138.15 (C-8), 132.28 (C-3″), 131.02 (C-2″), 120.26 (C-5), 87.19 (C-1′), 85.85 (C-4′), 75.68 (C-1″), 74.30 (C-2′), 70.75 (C-3′), 61.74 (C-5′), 40.14 (C-8″), 36.05 (C-5″), 35.74 (C-4″), 28.72 (C-6″), 24.02 (C-7″) ppm.

HRMS (ESI+): m/z calculated for $C_{19}H_{25}N_5O_7Na$: 458.16462 $[M+Na]^+$; found: 458.16463.

4.5 Synthesis of *N*^{2}-(2-*cis*-cyclooctyl)oxycarbonylguanosine (CCO-Guanosine)

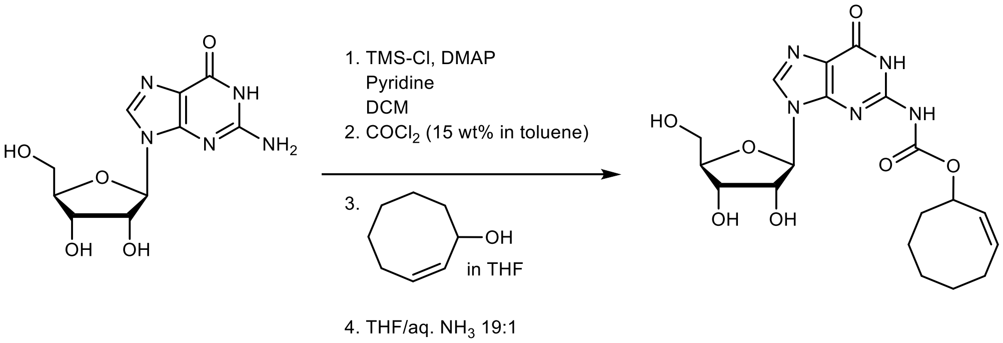

Guanosine (850 mg, 3 mmol, 1.0 eq.) and DMAP (40 mg, 0.35 mmol, 0.1 eq.) were suspended in dry DCM (60 mL) under argon atmosphere. The mixture was cooled to 0 °C and dry pyridine (10 mL) was added. Trimethylsilylchloride (2.5 mL, 18.9 mmol,

6.3 eq.) was added dropwise and the reaction was stirred for 2 hours. Next, phosgene (3 mL, 15 wt% solution in toluene, 4.2 mmol, 1.4 eq.) was added dropwise at 0 °C giving a yellow precipitate. The reaction was stirred for 30 min. Lastly, *cis*-cyclooct-2-en-1-ol (1.44 g, 11.3 mmol, 3.8 eq.) dissolved in dry THF (52 mL) was added and the reaction was stirred overnight while warming up to room temperature. Then, chloroform (100 mL) and water (100 mL) were added and the organic layer was washed with water (3 x 100 mL) and concentrated under reduced pressure. The obtained brown oily solid was dissolved in THF (50 mL), aqueous ammonia (2.5 mL, 25% solution) was added and the solution was stirred vigorously overnight. The obtained precipitate was centrifuged (3220 rcf, 4 °C, 10 min), the supernatant was removed and the precipitate was purified on a puriFlash system (PF50-SIHP F0120 column; 0-20% MeOH in DCM). Fractions containing the product were combined and concentrated. After lyophilisation the product was obtained as an off-white solid (90 mg, 0.21 mmol, 7%).

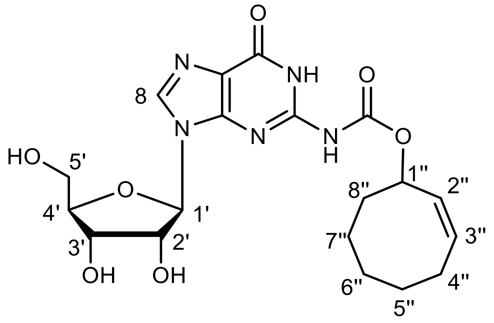

^{1}H NMR (500 MHz, DMSO) δ = 11.42 (m, 2H, 2N-H), 8.21 (s, 1H, H-8), 5.76 (d, J = 5.9 Hz, 1H, H-1′), 5.69 (dddd, J = 10.8, 9.1, 7.2, 1.5 Hz, 1H, H-3”), 5.63 (m, 1H, H-1”), 5.55 (ddd, J = 10.8, 6.9, 1.1 Hz, 1H, H-2”), 5.43 (d, J = 5.9 Hz, 1H OH 2’), 5.14 (d, J = 4.7 Hz, 1H, OH 3’), 4.97 (t, J = 5.5 Hz, 1H, OH 5’), 4.47 (qd, J = 5.8, 1.8 Hz, 1H, H-2’), 4.13 (m, 1H, H-3’), 3.89 (q, J = 4.1 Hz, 1H, H-4’), 3.66 – 3.5 (m, 2H, 2H-5′), 3.14 (m, 1H, CCO-CH_2), 2.20 (m, 1H, CCO-CH_2), 2.12 (m, 1H, CCO-CH_2), 1.98 (m, 1H, CCO-CH_2), 1.66 (m, 1H, CCO-CH_2), 1.55 (m, 4H, CCO-CH_2), 1.37 (m, 1H, CCO-CH_2).

4.6 Synthesis of N^2-(2-*trans*-cyclooctyl)oxycarbonylguanosine-5′-monophosphate (TCO-GMP, **2**)

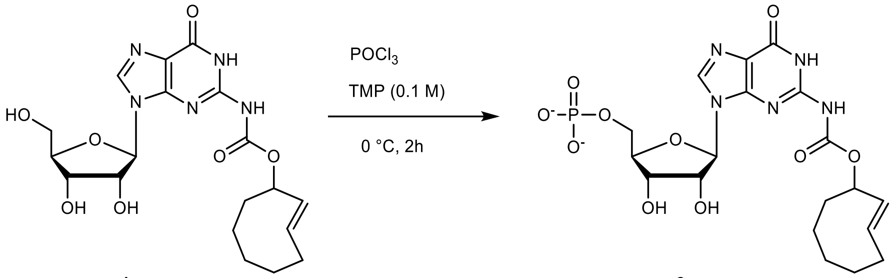

1 **2**

The Guanosine derivative **1** (251 mg, 0.576 mmol, 1.0 eq.) was suspended in dry trimethylphosphate (TMP, 5.8 mL) under argon. The solution was cooled to 0 °C and freshly distilled $POCl_3$ (141 mg, 85 µL, 921 µmol, 1.6 eq.) was added dropwise before stirring for two hours. Then, the reaction was carefully quenched with 1 M triethylammonium bicarbonate (TEAB) solution until no more bubbles were evolving. The resulting solution was washed with methyl *tert*-butyl ether (MTBE, 10 x 60 mL) and ethylacetate (1 x 60 mL), the aqueous part was dried by argon bubbling and concentrated *in vacuo*. The residue was purified by reversed-phase flash chromatography (aq-C18 column 150 grams, eluent: TEAB 0.1 M/acetonitrile, gradient: 0 – 50% of acetonitrile over 15 min). Fractions containing the product were freeze-dried separately and analysed by HPLC to monitor the purity of the fractions. The product was obtained as triethylammonium salt as off-white solid (228 mg, 0.44 mmol, 55%).

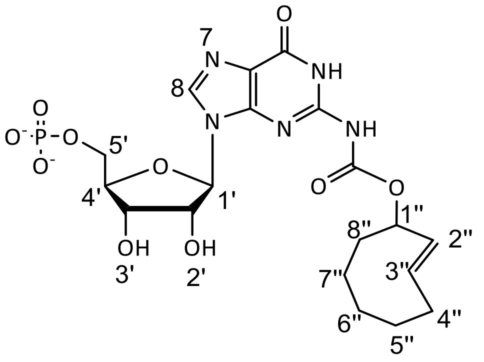

^{1}H NMR (599 MHz, D_2O) δ = 8.36 (d, J = 1.0 Hz, 1H, H-8), 6.05 (d, J = 5.9 Hz, 1H, H-1′), 5.92 (ddd, J = 15.7, 11.4, 3.8 Hz, 1H, H-3″), 5.68 (dd, J = 16.7, 2.3 Hz, 1H, H-2″), 5.40 (s, 1H, H-1″), 4.81 (m, 1H, H-2′), 4.53 (dt, J = 5.4, 2.7 Hz, 1H, H-3′), 4.35 (m, 1H, H-4′), 4.06 (m, 2H, H-5′), 2.44 (m, 1H, H-4″a), 2.12 (m, 1H, H-8″a), 2.04 (qd, J = 11.4, 5.0 Hz, 1H, H-4″b), 1.96 (m, 1H, H-5″a), 1.84 m, 2H, H-6″a, H-8″b), 1.69 (m, 1H, H-

7″a), 1.52 (qd, J = 12.9, 4.9 Hz, 1H, H-5″b), 1.08 (m, 1H, H-7″b), 0.84 (m, 1H, H-6″b) ppm.

^{13}C NMR (151 MHz, D_2O) δ = 157.65, 154.67, 150.05, 147.88, 139.47 (C-8), 133.21 (C-3″), 130.01 (C-2″), 119.30 (C-5), 87.18 (C-1′), 84.59 (C-4′), 77.17 (C-1″), 74.24 (C-2′), 70.77 (C-3′), 63.90 (C-5′), 39.72 (C-8″), 35.51 (C-4″, C-5″), 28.33 (C-6″), 23.58 (C-7″) ppm.

^{31}P NMR {^{1}H, ^{13}C} (202 MHz, D_2O) δ = 0.01 ppm.

HRMS (ESI-): m/z calculated for $C_{19}H_{25}N_5O_{10}P$: 514.13445 [M + H]$^-$; found: 514.13387.

4.7 Synthesis of *N*^{2}-(2-*cis*-cyclooctyl)oxycarbonylguanosine-5′-monophosphate (CCO-GMP)

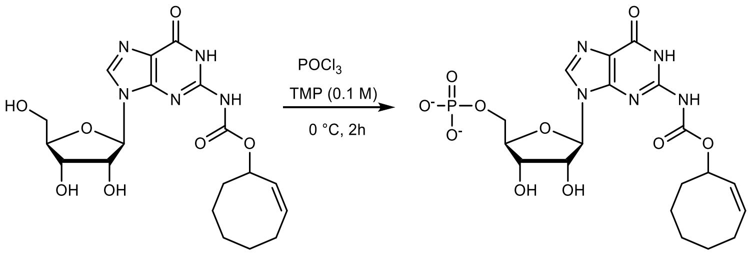

In a Schlenk tube guanosine (45 mg, 0.103 mmol, 1 Eq.) was suspended in dry trimethylphosphate (TMP, 2 mL) under argon. The solution was cooled to 0 °C and $POCl_3$ (42 mg, 25 µL, 275 µmol, 2.7 Eq.) was added dropwise before stirring for 3 hours. The reaction was carefully quenched with triethylammonium bicarbonate solution (50 mM) until no more bubbles were evolving. The resulting solution was tested with pH paper (~7 – 8) and washed with methyltertbutylether (MTBE, 10 x 20 mL) and ethylacetate (1 x 20 mL) for removal of the TMP. The aqueous part was dried by Argon bubbling and concentrated *in vacuo*. The residue was used without further purification (yield not determined).

MS (ESI-): m/z calculated: 514.1 [M-H]$^-$; found: 514.3.

4.8 Synthesis of N^2-(2-*trans*-cyclooctyl)oxycarbonyl-7-methylguanosine-5′-monophosphate (TCO-m⁷GMP, **3**)

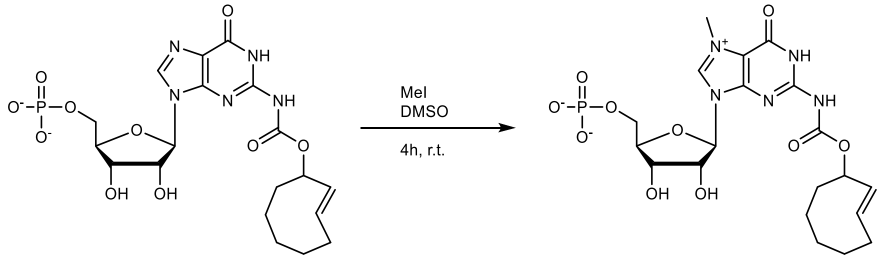

The triethylammonium salt of axial TCO-GMP **2** (153 mg, 0.298 mmol, 1 eq.) was dissolved in dry DMSO (2.1 mL) under argon. Iodomethane (363 µL, 0.83 g, 5.8 mmol, 19.5 eq.) was added dropwise and the reaction was stirred for 4 hours at room temperature. Then, ice-cold TEAB (1.2 mL, 1 M) was added and after 5 minutes stirring, the reaction mixture was purified by reversed-phase chromatography (C18-aq 150 g, Eluent: TEAB (0.1 M)/acetonitrile, gradient: 0 – 50% acetonitrile over 15 min). Fractions containing the product were analysed by HPLC and freeze-dried separately to yield the product as an off-white fluffy lyophilizate (52 mg, 98 µmol, 39%).

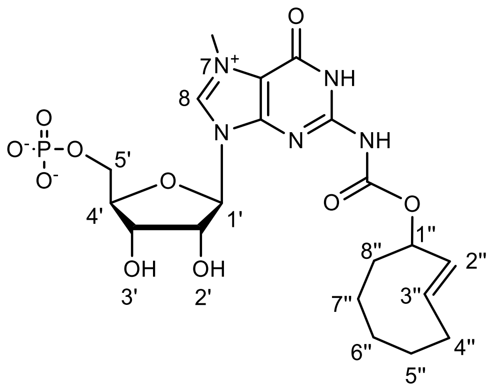

^{1}H NMR (500 MHz, D_2O) δ = 6.21 (dd, J = 3.7, 2.0 Hz, 1H, H-1′), 5.90 (ddd, J = 15.8, 12.1, 5.9 Hz, 1H, H-3″), 5.70 (m, 1H, H-2″), 5.38 (s, 1H, H-1″), 4.68 (dt, J = 4.8, 3.4 Hz, 1H, H-2′), 4.51 (td, J = 5.0, 2.9 Hz, 1H, H-3′), 4.43 (dt, J = 5.3, 2.1 Hz, 1H, H-4′), 4.22 (m, 1H, H-5′a), 4.19 (s, 3H, N^7-Me), 4.08 (tdd, J = 11.9, 4.8, 2.3 Hz, 1H, H-5′b), 2.45 (m, 1H, H-4″a), 2.12 (m, 1H, H-8″a), , 2.06 (m, 1H, H-4″b), 1.96 (m, 1H, H-5″a), 1.86 (m, 1H, H-6″a), 1.83 (m, 1H, H-8″b), 1.68 (m, 1H, H-7″a), 1.51 (m, 1H, H-5″b), 1.06 (m, 1H, H-7″b), 0.85 (m, 1H, H-6″b) ppm.

^{13}C NMR (126 MHz, D_2O) δ = 157.90, 152.25, 136.84 (C-3″), 134.26 (C-2″), 115.76 (C-5), 93.81 (C-1′), 88.80 (C-4′), 80.37 (C-1″), 79.39 (C-2′), 73.55 (C-3′), 66.86 (C-5′), 43.64 (C-8″), 39.91 (N^7-CH_3), 39.42 (C-5″), 39.37 (C-4″), 32.23 (C-6″), 27.48 (C-7″) ppm.

^{31}P NMR {^1H, ^{13}C} (202 MHz, D_2O) δ = 6.13 ppm.

HRMS (ESI-): m/z calculated for $C_{20}H_{27}N_5O_{10}P$: 528.15010 $[M]^-$; found: 528.14965.

4.9 Synthesis of P1-(N^2-(2-*trans*-cyclooctyl)oxycarbonyl-7-methylguanosine-5′-yl)-P3-guanosin-5′-yl triphosphate (TCO-cap, **4**)

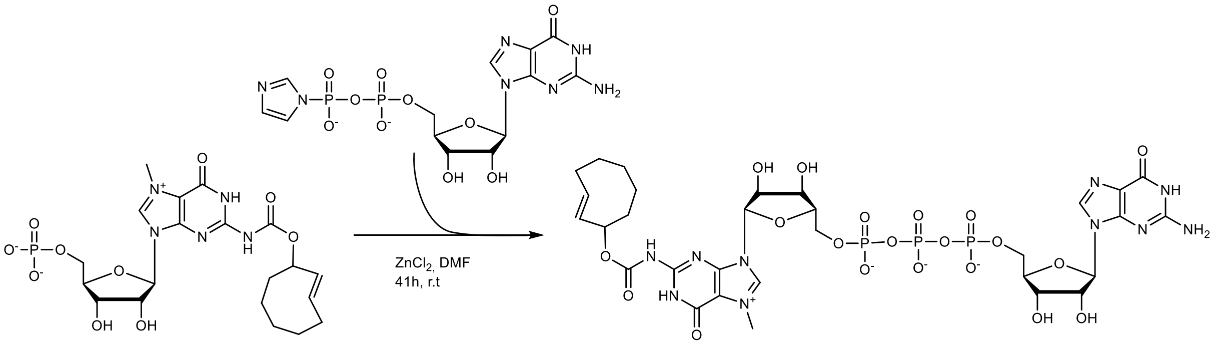

A solution of the triethylammonium salt of TCO-m^{7}GMP **3** (52 mg, 98 µmol, 1 eq.) in dry DMF (2.4 mL) was added to dried $ZnCl_2$ (577 mg, 4.23 mmol, 43 eq.). After addition of GDP-imidazolide (159 mg, 0.295 mmol, 3 eq.), the reaction mixture was stirred for 41 hours at room temperature. Thereafter, ddH_2O (1.2 mL) and EDTA-solution (0.5 M, 1.8 mL) were added and the reaction mixture was stirred for 10 min before purification by reversed-phase chromatography (C18-aq 150 g column, eluent: TEAB (0.1 M)/acetonitrile, gradient 0- 50%). The product containing fractions were analysed by HPLC and frieze-dried to obtain the product as off-white solid in form of its triethylammonium salt (22.9 mg, 24 µmol, 24%).

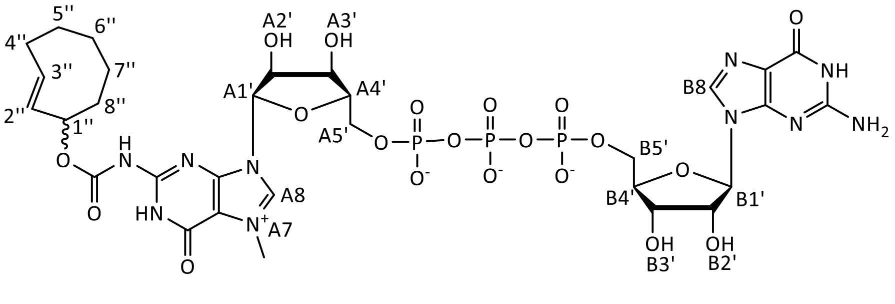

^{1}H NMR (599 MHz, D_2O) δ = 8.03 (s, 1H, H-B8), 6.07 (t, J = 3.7 Hz, 1H, H-A1′), 5.91 (m, 1H, H-3″), 5.81 (d, J = 6.2 Hz, 1H, H-B1′), 5.69 (ddd, J = 16.6, 4.3, 2.3 Hz, 1H, H-2″), 5.35 (s, H-1″), 4.69 (ddd, J = 6.3, 5.2, 1.2 Hz, 1H, H-B2′), 4.58 (ddd, J = 5.0, 3.5, 1.8 Hz, 1H, H-A2′), 4.49 (m, 1H, H-B3′), 4.46 (m, 1H, A-3′), 4.40 (m, 1H, H-A4′), 4.37 (m, 1H, H-A5′a), 4.34 (m, 1H, H-B4′), 4.28 (m, 1H, H-A5′b), 4.26 (m, 2H, H-B5′), (4.13 d, J = 1.0 Hz, 3H, NA7-CH_3), 2.45 (m, 1H, H-4″a), 2.11 (m, 1H, H-8″a), 2.06 (m, 1H, H-4″b), 1.96 (m, 1H, H-5″a), 1.85 (m, 1H, H-6″a), 1.82 (m, 1H, H-8″b), 1.67 (m, 1H, H-7″a), 1.50 (m, 1H, H-5″b), 1.06 (m, 1H, H-7″b), 0.85 (m, 1H, H-6″b) ppm.

^{13}C NMR (151 MHz, D_2O) δ = 158.64, 155.61, 153.92, 151.56, 148.39, 137.38 (C-B8), 132.92 (C-3″), 130.64 (C-2″), 116.05 (C-B5), 111.76 (C-A5), 89.76 (C-A1′), 86.69 (C-B1′), 84.09 (C-A4′), 83.83 (C-B4′), 76.06 (C-1″), 75.32 (C-A2′), 73.77 (C-B2′), 70.52 (C-B3′), 69.33 (C-A3′), 65.50 (C-B5′), 64.45 (C-A5′), 39.81 (C-8″), 36.02 (C-A^{7}N-CH_3), 35.54 (C-5″), 35.50 (C-4″), 28.39 (C-6″), 23.64 (C-7″) ppm.

^{31}P NMR {^{1}H, ^{13}C} (243 MHz, D_2O) δ = -11.69 (m, 2P), -23.29 (td, J = 19.6, 1.9 Hz, 1P) ppm.

HRMS (ESI-): m/z calculated for $C_{30}H_{40}N_{10}O_{20}P_3$: 953.16387 $[M+H]^-$; found: 953.16348.

5. Supplementary Figures

5.1 Binding of eIF4E to m⁷GpppG

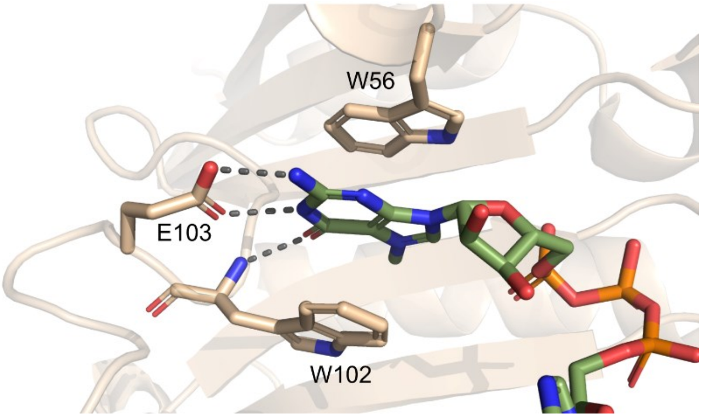

Figure S2: eIF4E interacts with m^7GpppG via the Watson-Crick site of m^7G and π- π stacking to the m^7G (PDB ID: 1IPB)[4]. The TCO modification at the N^2-position of m^7GpppG of the TCO-Cap interrupts the interaction with the Watson-Crick site, thereby inhibiting binding of eIF4E.

5.2 Irradiation apparatus

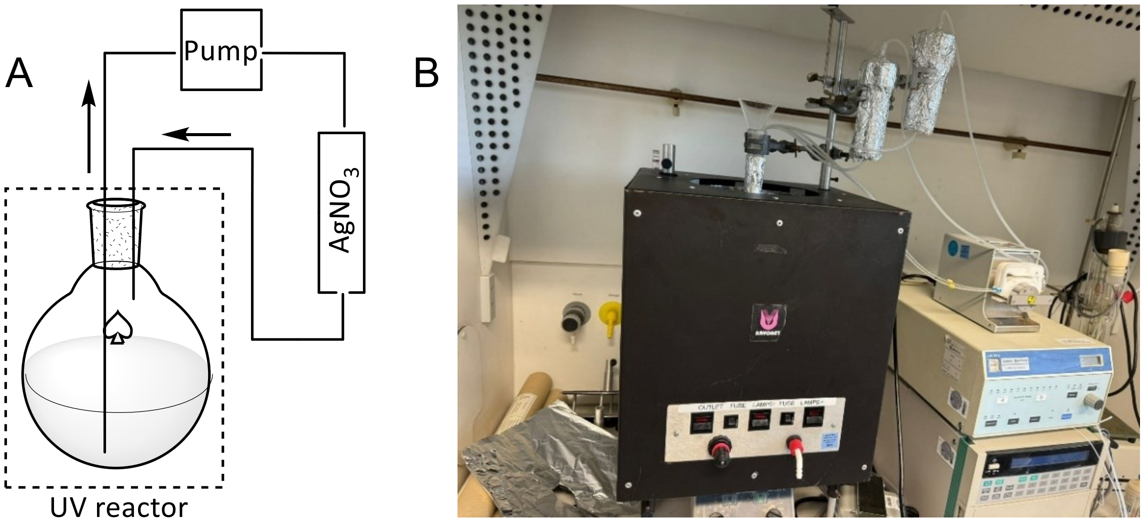

Figure S3: Irradiation Apparatus for the Photo-Isomerization of *cis*-Cyclooct-2-en-1-ol to obtain *trans*-Cyclooct-2-en-1-ol (TCO); A: Schematic drawing, B: Photo of the irradiation setup.

5.3 Click-to-release studies

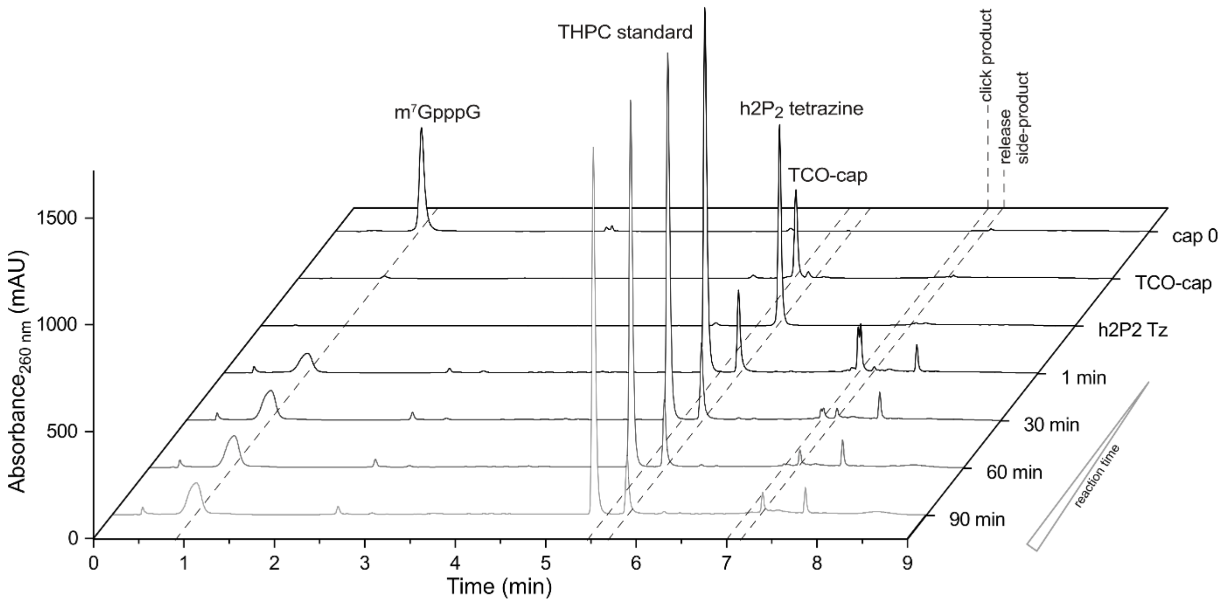

Figure S4: Representative HPLC traces of the click-to-release reaction at different time points between the TCO-cap **4** and tetrazine **5** ($h2P_2$) in sodium phosphate buffer in vitro using 3.3 eq. of tetrazine. Analysis with 0 – 25% acetonitrile in K-phosphate buffer as gradient.

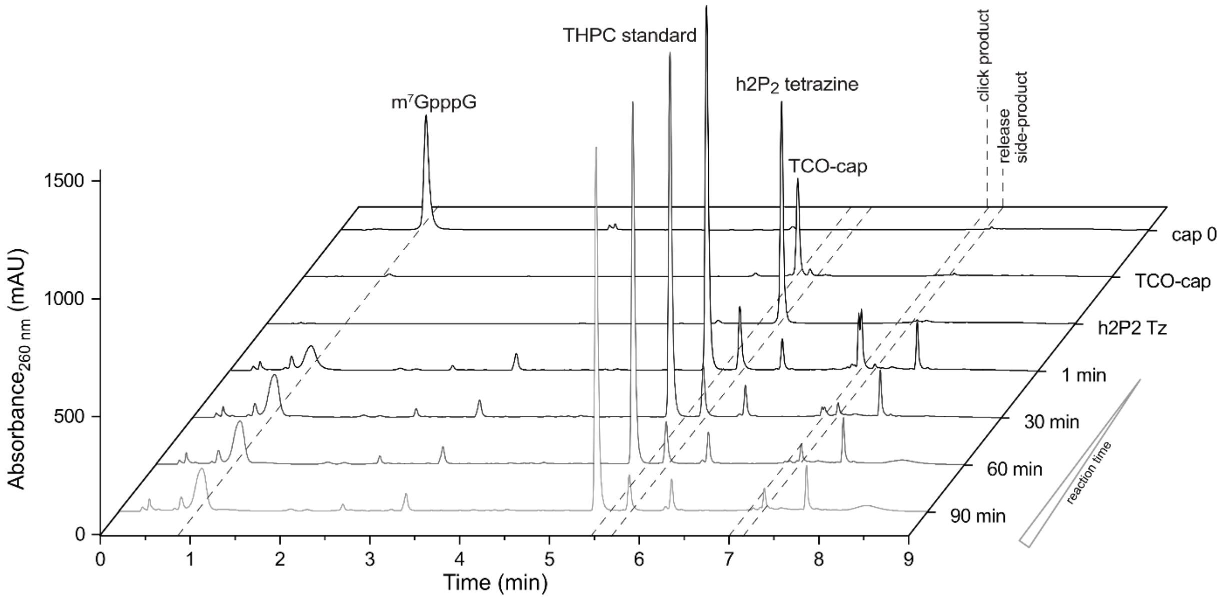

Figure S5: Representative HPLC traces of the click-to-release reaction at different time points between the TCO-cap **4** and tetrazine **5** ($h2P_2$) in cell culture medium (MEM) in vitro using 3.3 eq. of tetrazine. Analysis with 0 – 25% acetonitrile in K-phosphate buffer as gradient.

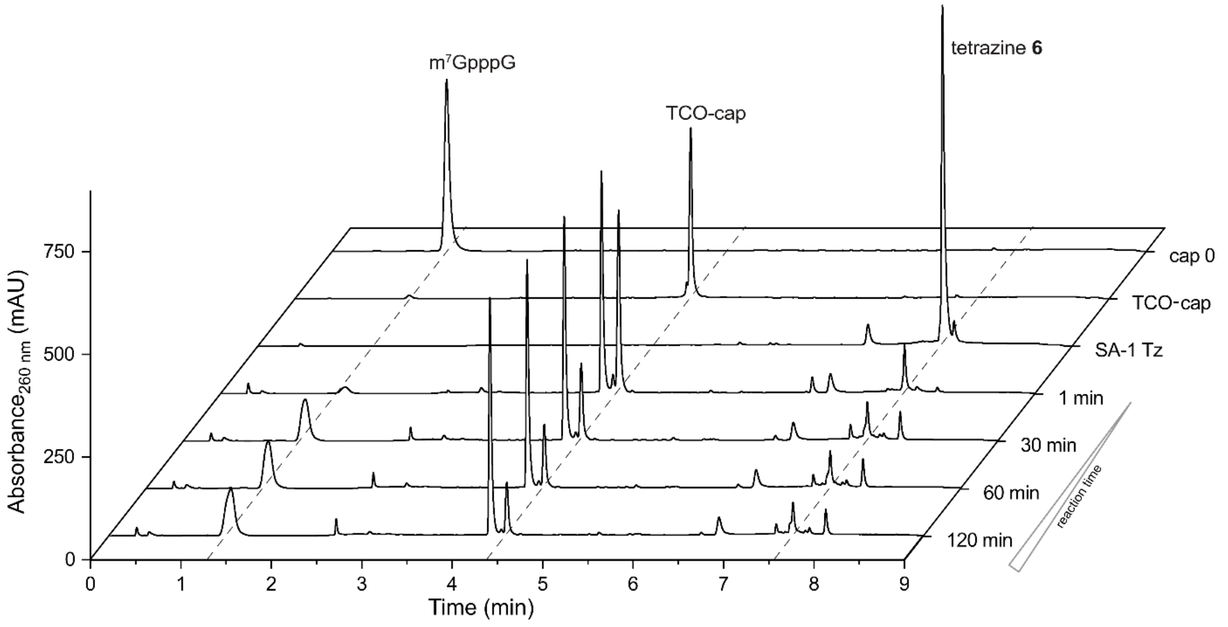

Figure S6: Representative HPLC traces of the click-to-release reaction at different time points between the TCO-cap **4** and tetrazine **6** (SA-1) in sodium phosphate buffer in vitro using 3.3 eq. of tetrazine. Analysis with 0 – 50% acetonitrile in NH_4OAc buffer as gradient.

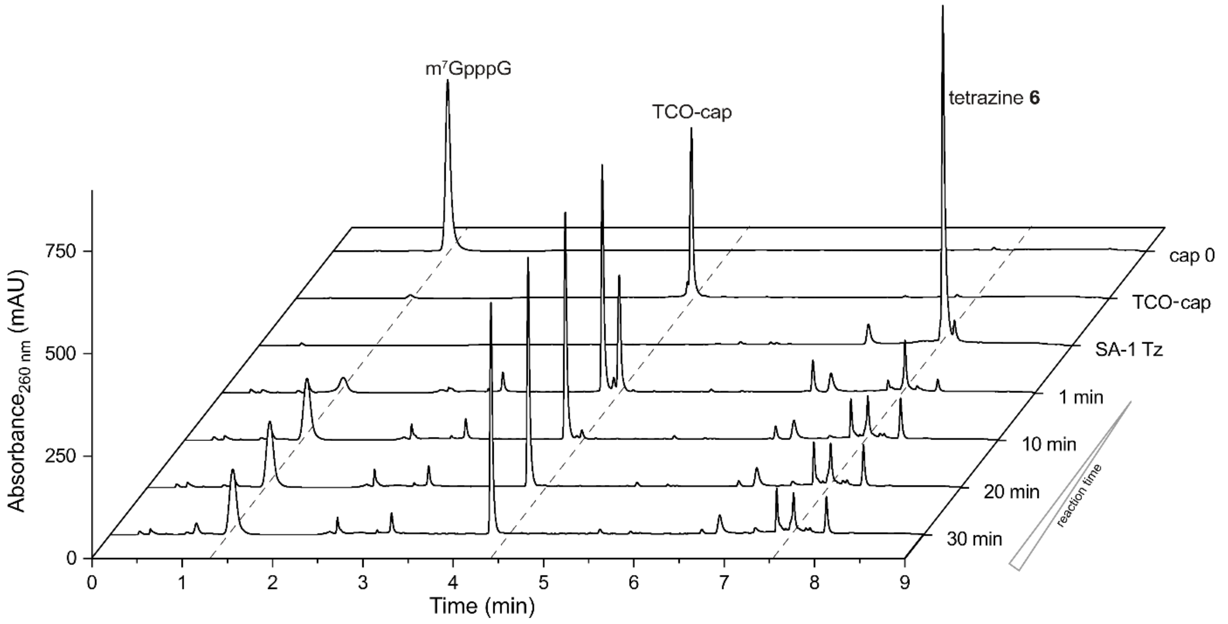

Figure S7: Representative HPLC traces of the click-to-release reaction at different time points between the TCO-cap **4** and tetrazine **6** (SA-1) in cell culture medium (MEM) in vitro using 3.3 eq. of tetrazine. Analysis with 0 – 50% acetonitrile in NH_4OAc buffer as gradient.

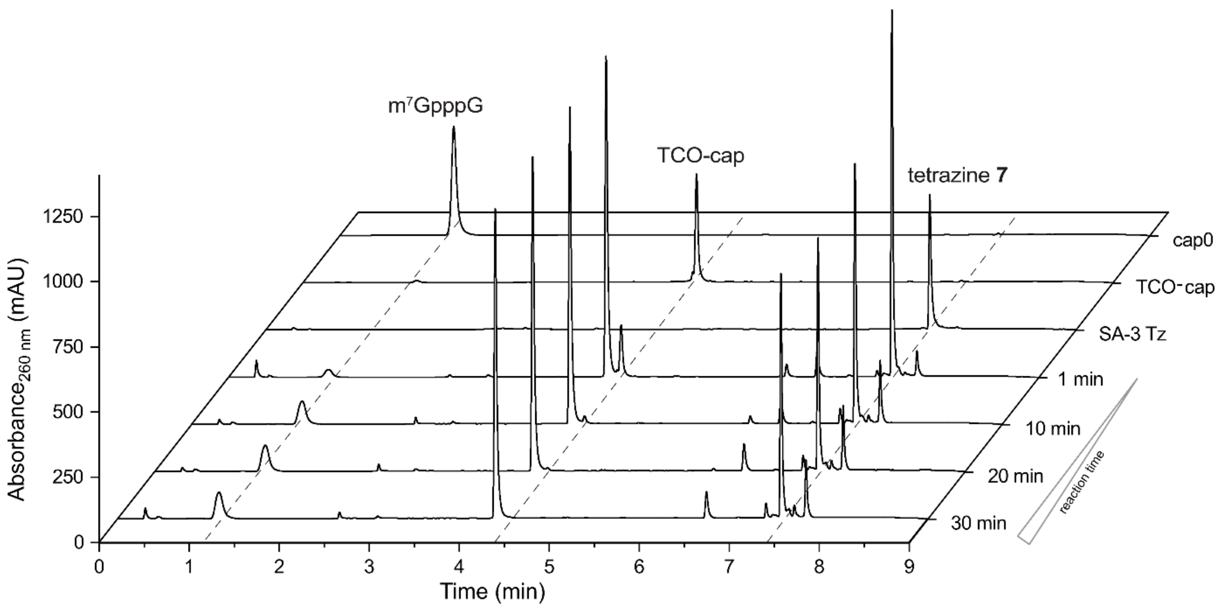

Figure S8: Representative HPLC traces of the click-to-release reaction at different time points between the TCO-cap **4** and tetrazine **7** (SA-3) in sodium phosphate buffer in vitro using 3.3 eq. of tetrazine. Analysis with 0 – 50% acetonitrile in NH_4OAc buffer as gradient.

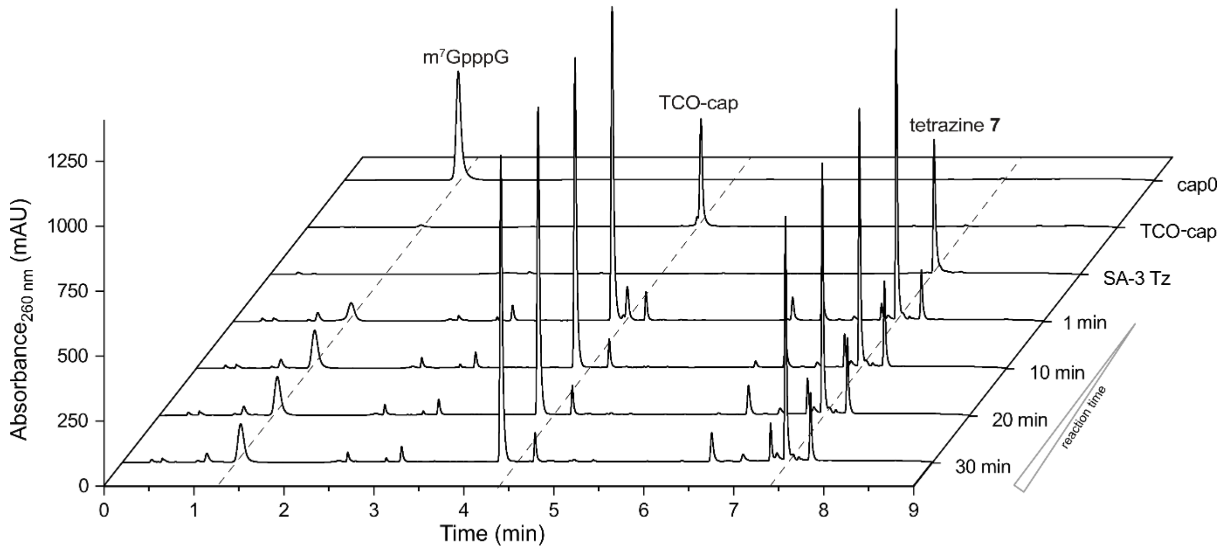

Figure S9: Representative HPLC traces of the click-to-release reaction at different time points between the TCO-cap **4** and tetrazine **7** (SA-3) in cell culture medium (MEM) in vitro using 3.3 eq. of tetrazine. Analysis with 0–50% acetonitrile in NH_4OAc buffer as gradient.

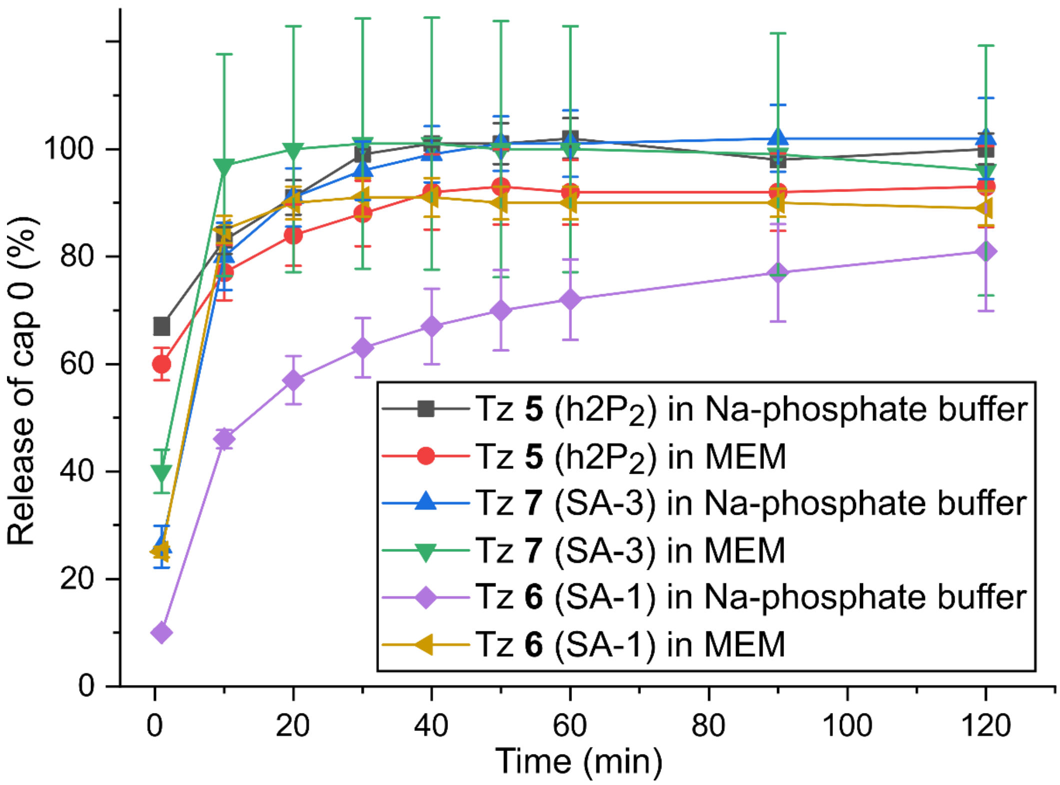

Figure S10: Comparative evaluation of the kinetic click-to-release studies of the reaction between the TCO-cap **4** and tetrazines **5** to **7** in sodium phosphate buffer and MEM medium (mean values and SE for n=3 independent experiments are shown).

5.4 Click-to-release on LC-MS

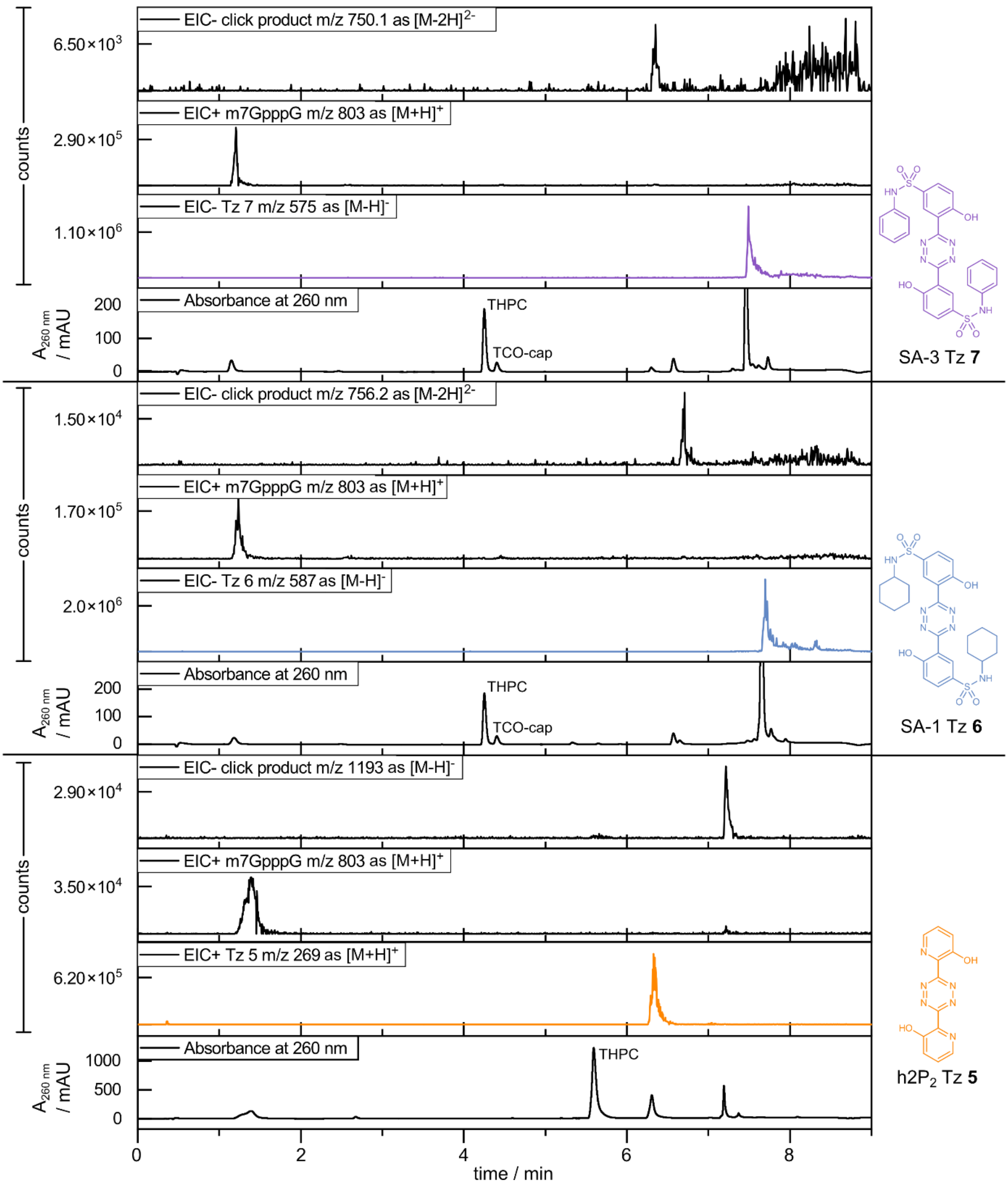

Figure S11: LC-MS of the click-to-release reaction between the TCO-cap **4** (60 µM) and 3.3 eq. h2P$_2$ tetrazine **5**, 10 eq. SA-1 tetrazine **6** or 3.3 eq. SA-3 tetrazine **7** in TEAA buffer (50 mM, pH 7) in presence of 0.4 mM trihydroxyphenylcoumarine (THPC) as internal standard after 10 min at 37 °C; For tetrazine **5** a gradient of 0 – 25% acetonitrile in NH_4OAc buffer was applied, for tetrazines **6** and **7** 0 – 50% acetonitrile in NH_4OAc were used. The TCO-cap was still detected after 10 minutes in presence of tetrazines **6** and **7** and identified as $[M+H]^+$ at *m/z* 955 (data not shown for clarity).

5.5 Cell lysate stability of the TCO-cap

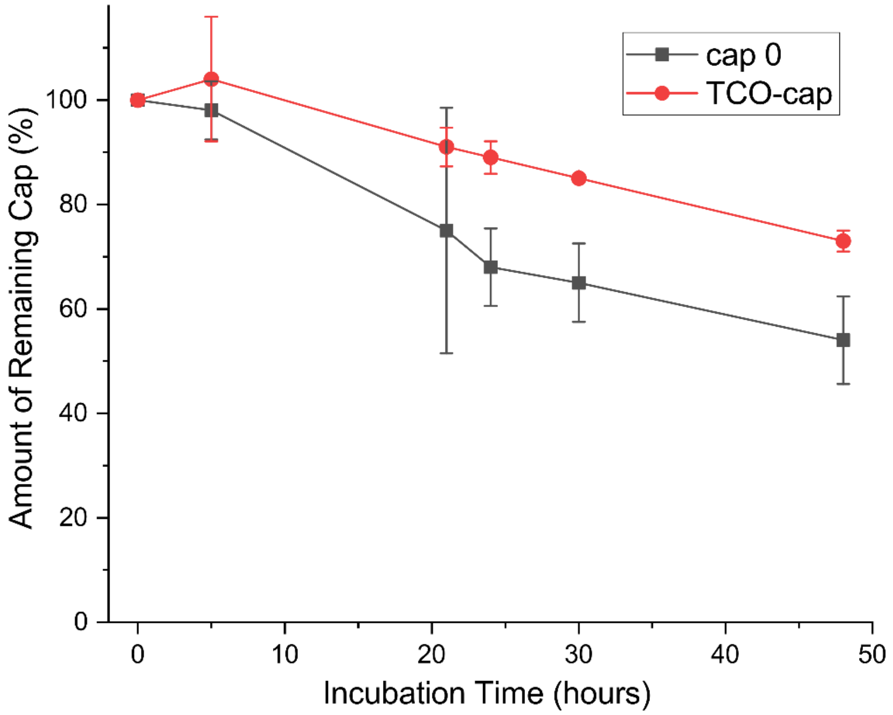

Figure S12: Evaluation of the stability of the TCO-cap (0.15 mM) and cap 0 (0.21 mM) in HeLa cell lysate for 48 hours at 37 °C; Analysis on HPLC (mean values and SE for n=3 independent experiments are shown).

Table S5: Mean values and SE in % of remaining cap 0 and TCO-cap after indicated incubation times in HeLa cell lysate at 37 °C from n=3 independent experiments.

	0 h	0.5 h	1 h	5 h	21 h	24 h	30 h	48 h
cap 0	100 ± 0	106 ± 12	97 ± 6	98 ± 6	75 ± 24	68 ± 7	65 ± 8	54 ± 8
TCO-cap	100 ± 0	109 ± 7	105 ± 4	104 ± 12	91 ± 4	89 ± 3	85 ± 0	73 ± 2

5.6 Evaluation of TCO isomerization in cell culture medium

5.6.1 Isomerization in unsupplemented MEM

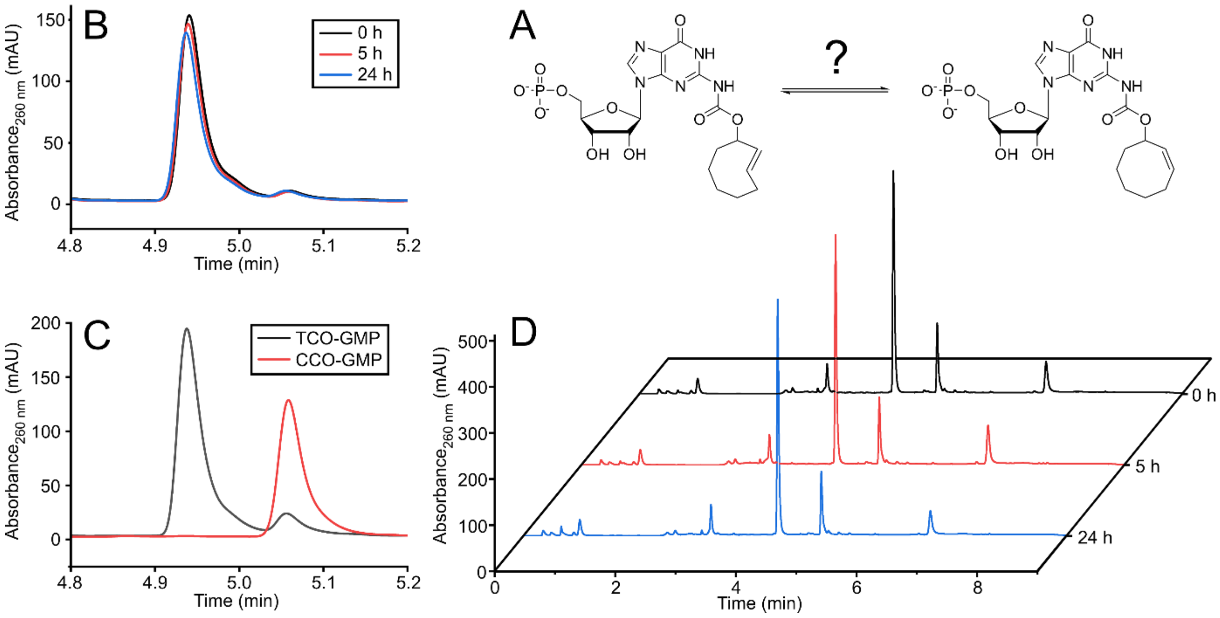

Figure S13: Analysis of the isomerization of the TCO moiety in cell culture medium (MEM) without supplementation. A shows the investigated isomerization reaction. Relevant reference compounds are shown in C. The samples containing 60 µM of compound **2** and 400 µM THPC as internal standard in the medium were incubated at 37 °C before measurement on HPLC after 0, 5 and 24 hours: D shows the whole chromatograms, B provides the relevant excerpt. Within the investigated time frame no isomerisation was observed as proven by a constant ratio of TCO-GMP and CCO-GMP (see Table S6). Shown is one of n = 3 independent measurements.

5.6.2 Isomerization in supplemented MEM

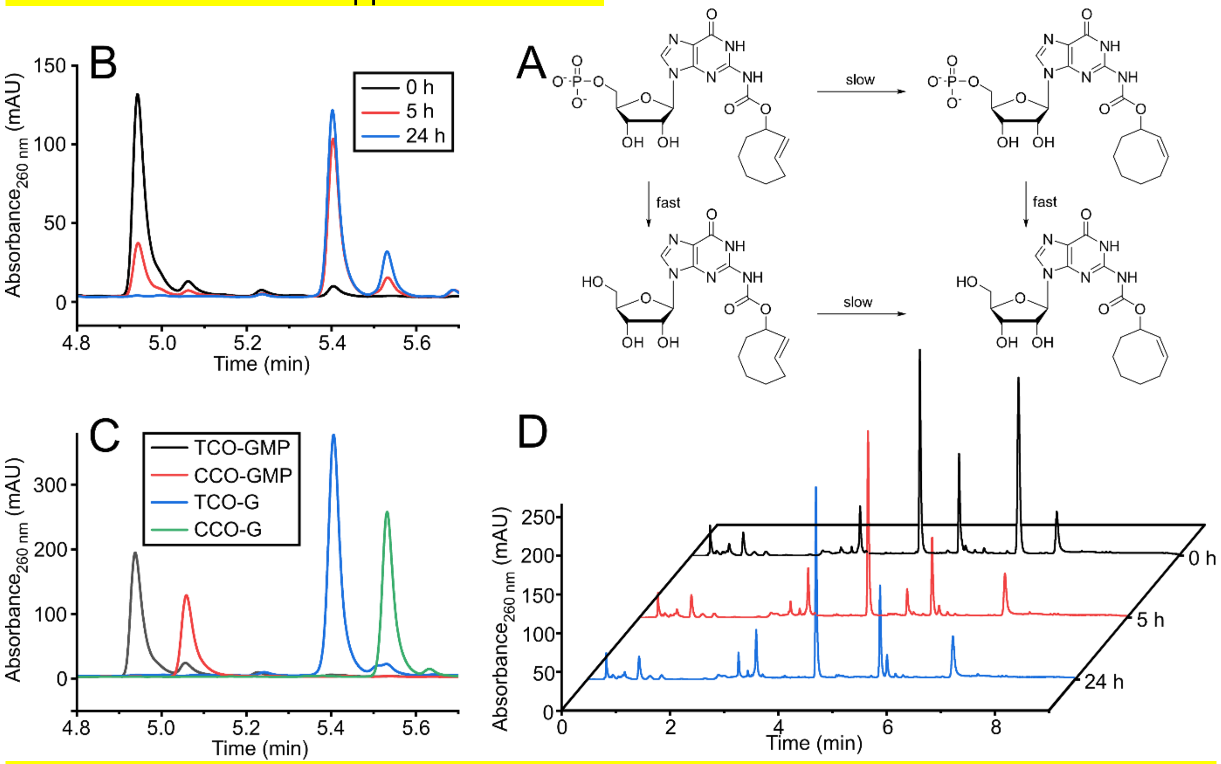

Figure S14: Analysis of the isomerization of the TCO moiety in cell culture medium (MEM) supplemented with 1% non-essential amino acids, 1% GlutaMAX and 10% fetal calf serum. A shows the observed isomerization reaction. Relevant reference compounds are shown in C. The samples containing 60 µM of compound **2** and 400 µM THPC as internal standard in the medium were incubated at 37 °C before measurement on HPLC after 0, 5 and 24 hours: D shows the whole chromatograms, B provides the relevant excerpt. Compound **2** was dephosphorylated as proven by the reference compound TCO-guanosine while slight isomerisation was observed (increase from 7% total CCO to 20% over 24 hours, Table S6). Shown is one of n = 3 independent measurements

Table S6: Results from the isomerisation study of TCO-GMP in cell culture medium from a triplicate measurement. Detected peak areas (mAU x min) corresponding to TCO-GMP, CCO-GMP, TCO-G and CCO-G after incubation for 0, 5 and 24 hours in supplemented or unsupplemented MEM. From the peak areas the relative amounts of the CCO-derivatives was calculated to see a potential increase over time due to isomerisation.

	**Unsupplemented MEM**					
	0 h		5 h		24 h	
	TCO-GMP	CCO-GMP	TCO-GMP	CCO-GMP	TCO-GMP	CCO-GMP
Rep 1	340	22 ≙ 6%	327	22 ≙ 6%	308	22 ≙ 7%
Rep 2	312	20 ≙ 6%	302	20 ≙ 6%	288	20 ≙ 6%
Rep 3	321	20 ≙ 6%	310	16 ≙ 5%	297	21 ≙ 7%
		Mean: 6%		Mean: 6%		Mean: 7%
	**Supplemented MEM**					
	0 h		5 h		24 h	
	TCO-GMP/G	CCO-GMP	TCO-GMP/G	CCO-GMP/G	TCO-GMP/G	CCO-G
Rep 1	316+14	27 ≙ 8%	89+224	13+31 ≙ 12%	4+275	66 ≙ 19%
Rep 2	290+14	20 ≙ 6%	80+214	12+30 ≙ 13%	252	63 ≙ 20%
Rep 3	275+13	24 ≙ 8%	72+201	11+29 ≙ 13%	233	61 ≙ 21%
		Mean: 7%		Mean: 13%		Mean: 20%

5.7 Evaluation of cell toxicity of the tetrazines using a MTT-assay

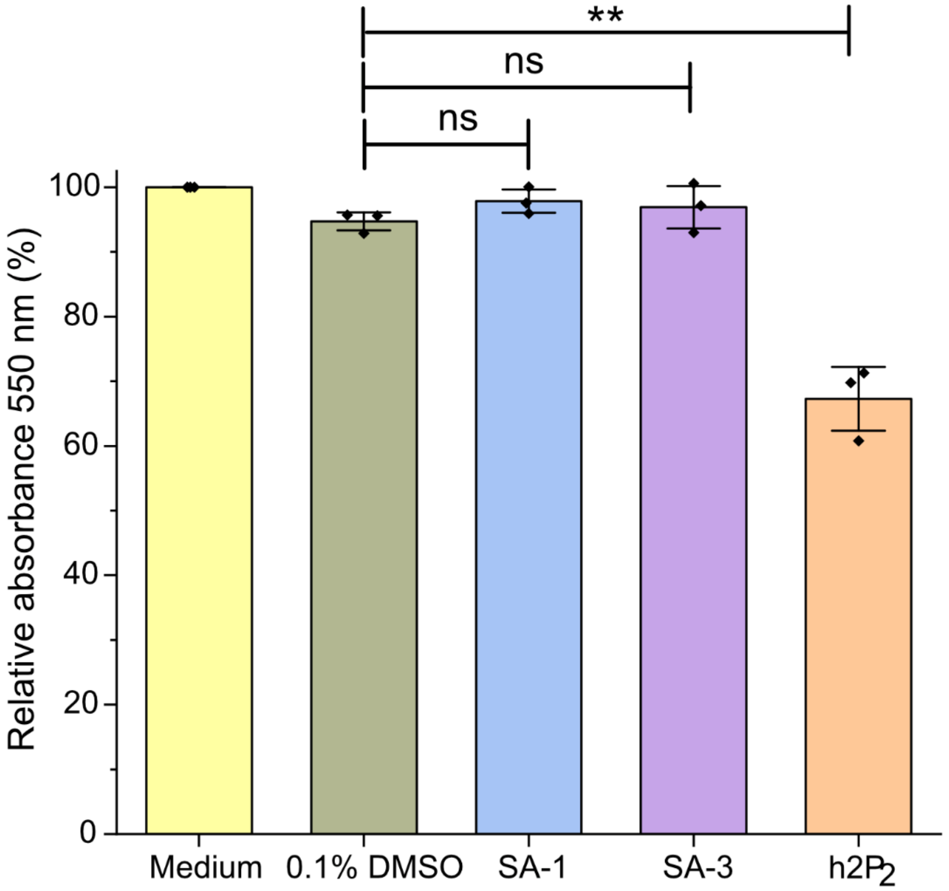

Figure S15: MTT assay to evaluate the toxicity of the tetrazines on HeLa cells (mean values and SE for n=3 independent experiments are shown. ** = $P<0.01$, ns= $P>0.05$, P-value for TCO-capped mRNA treated with DMSO versus h2P_2 is 1.3×10^{-3}, Student's T-test). After the MTT-assay, the absorbance was measured at 550 and the cell viability was set to 100% for HeLa cells treated with medium. Only h2P_2 treated cells show a significant decrease in cell viability compared to DMSO treated cells. MTT assay performed after transfection of HeLa cells with TCO-capped Gluc mRNA and subsequent treatment of the cells with 10 μM of the respective tetrazine.

5.8 In cell click-to-release of *Renilla* luciferase RNA

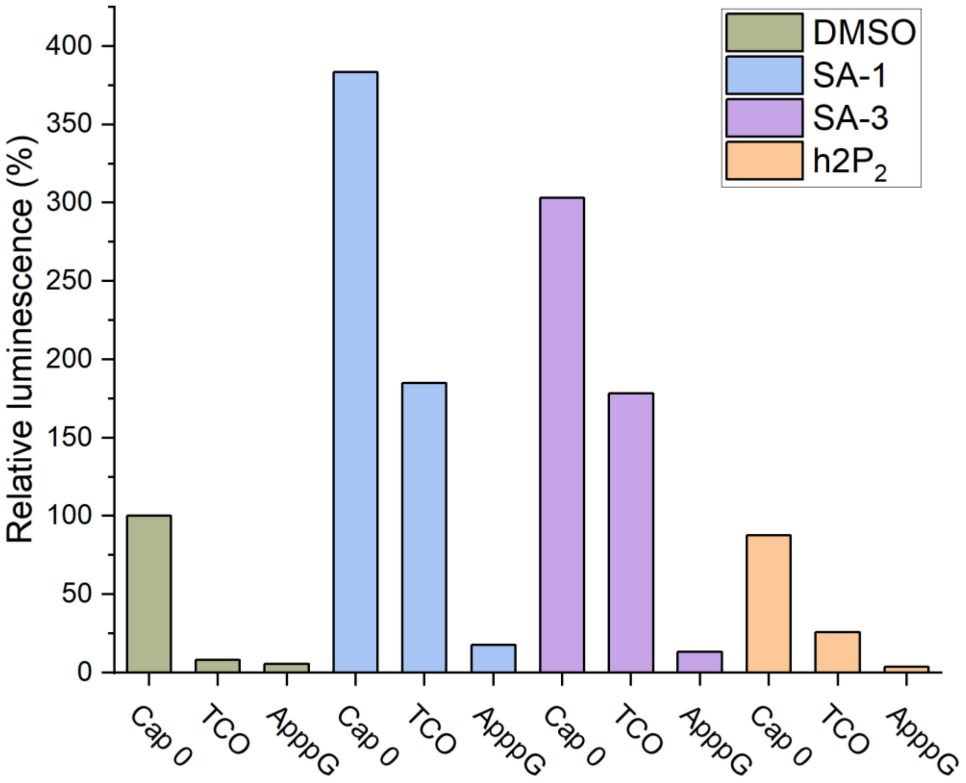

Figure S16: Luciferase activity from HeLa cells transfected with differently capped RLuc-mRNAs. All luciferase activities are normalized to HeLa cells transfected with cap 0-mRNA and treated with 0.1% DMSO (100 %). HeLa cells were transfected with differently capped RLuc-mRNA for 4 hours, and afterwards treated for 1 h with either 0.1% DMSO in HeLa medium, or 10 µM of the respective tetrazine in HeLa medium containing 0.1% DMSO. 24h after the start of the experiment the cells were lysed and the luciferase activity was determined. HeLa cells transfected with TCO-capped mRNA and treated with SA-1 and SA-3 yielded the highest luciferase activity with 185% and 178% respectively. The results made us decide to continue with SA-1 and SA-3 in further cell experiments.

5.9 In cell click-to-release of *Gaussia* luciferase mRNA using different experimental conditions

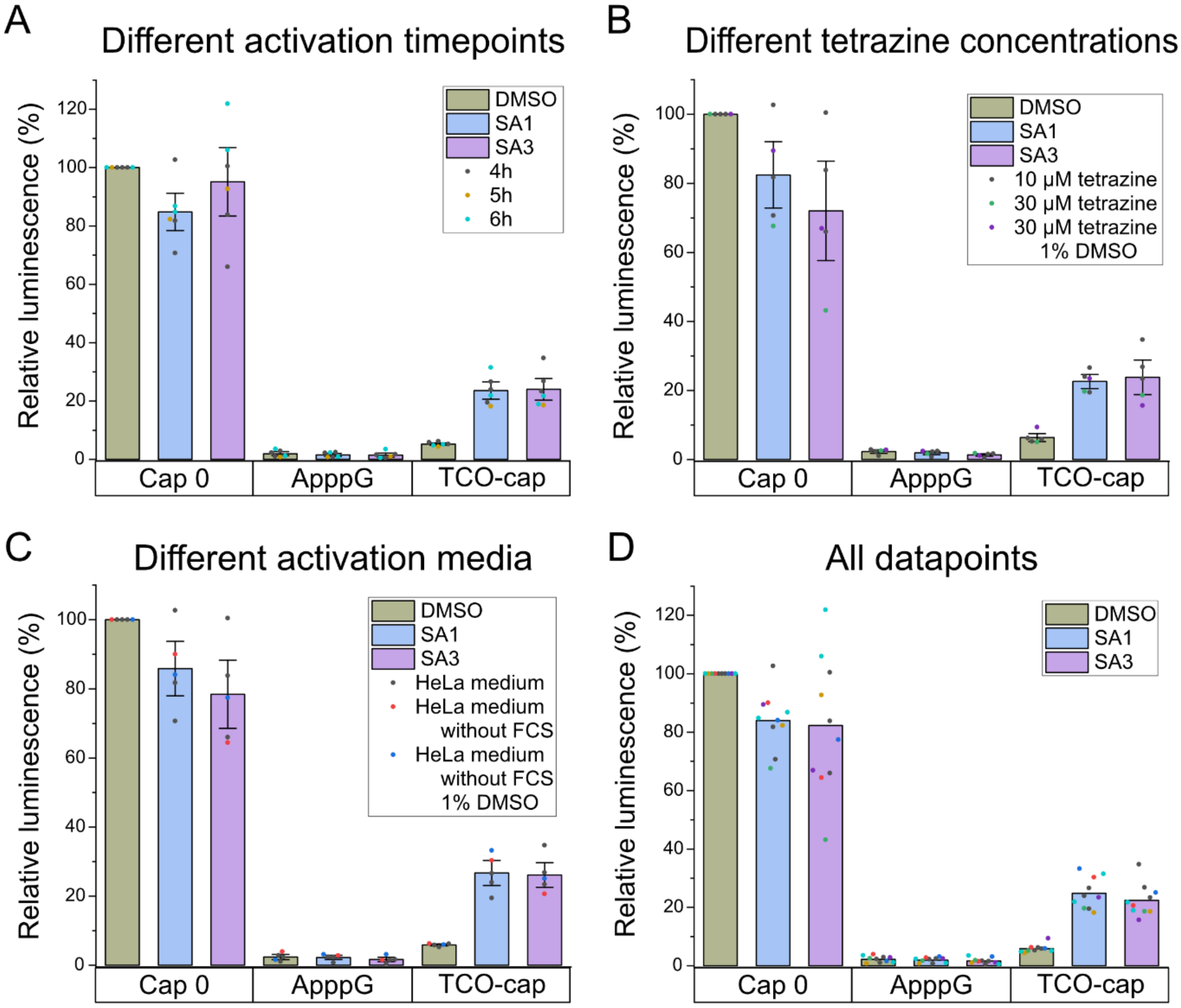

Figure S17: Luminescence measurements for cells transfected with differently capped GLuc-mRNA and addition of tetrazines SA-1/SA-3 or DMSO as control under different conditions. Every different experimental procedure is represented as a dot in a different color. A) Three different timepoints of activation with tetrazine after transfection were tested; 4h (n=3), 5h (n=1) and 6h (n=2). All three timepoints gave similar results. B) Two different tetrazine concentrations were tested under two different conditions; 10 µM tetrazine with 0.1% DMSO (n=3), 30 µM tetrazine with 0.1% DMSO (n=1) or 30 µM tetrazine with 1% DMSO (n=1). For all conditions a similar turn-on of translational output for HeLa cells transfected with TCO-capped mRNA was observed. C) Three different media were tested for the activation with tetrazine; HeLa medium (as defined in methods) (n=3), HeLa medium without FCS (n=1) and HeLa medium without FCS with 1% DMSO (n=1). All three conditions gave similar results. D) All datapoints from A,B and C, combined in one graph, shows the robustness of the tetrazine mediated activation among all different conditions.

5.10 HPLC Purification of different mRNAs

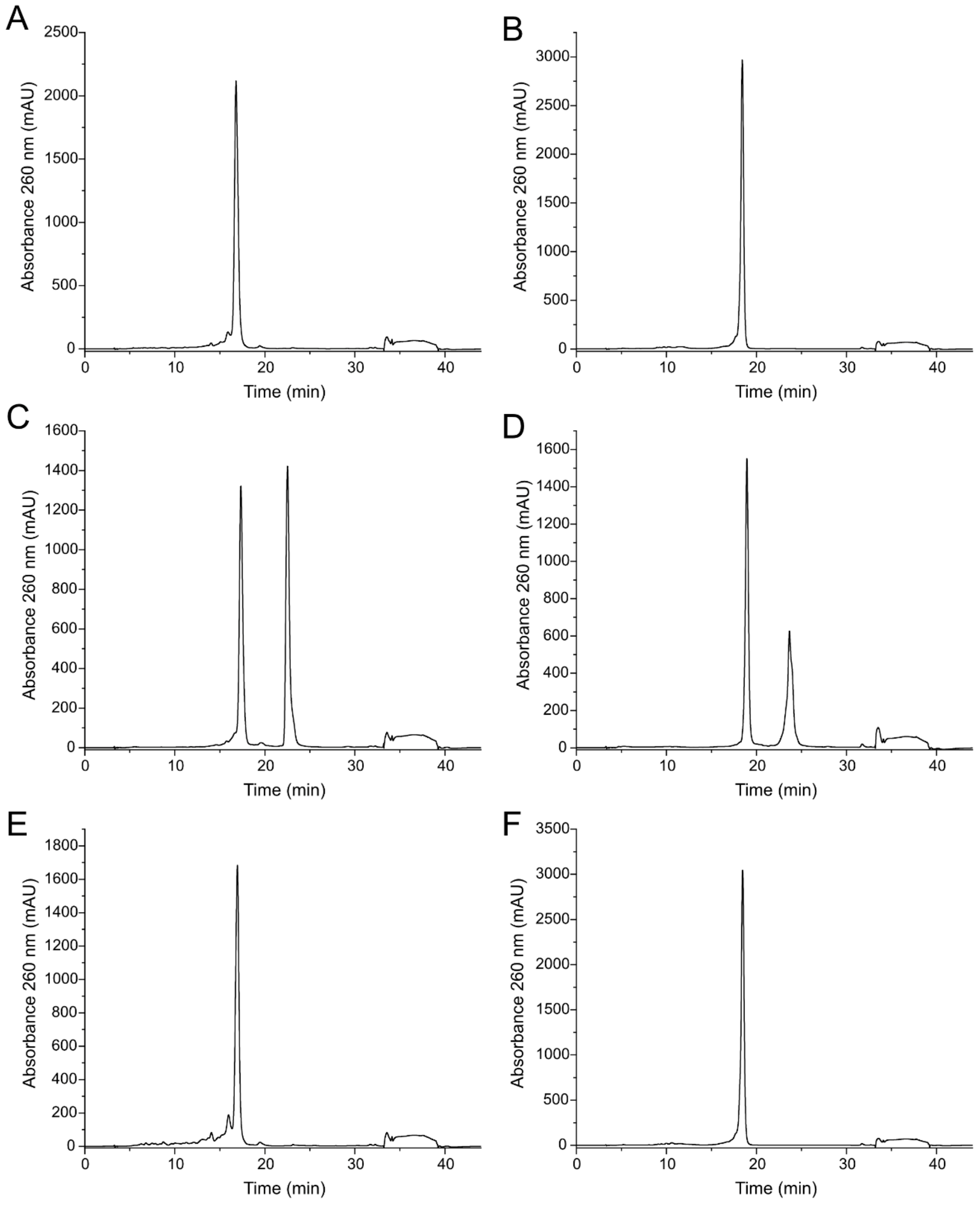

Figure S18 HPLC chromatograms of RNA purifications. A) Cap 0-GLuc-mRNA. B) Cap 0-GFP-mRNA (containing m^{1}Ψ and m^{5}C). C) TCO-capped GLuc-mRNA. D) TCO-capped GFP-mRNA (containing m^{1}Ψ and m^{5}C). E) ApppG-capped GLuc-mRNA. F) ApppG-capped GFP-mRNA (containing m^{1}Ψ and m^{5}C).

5.11 Uncropped gel image

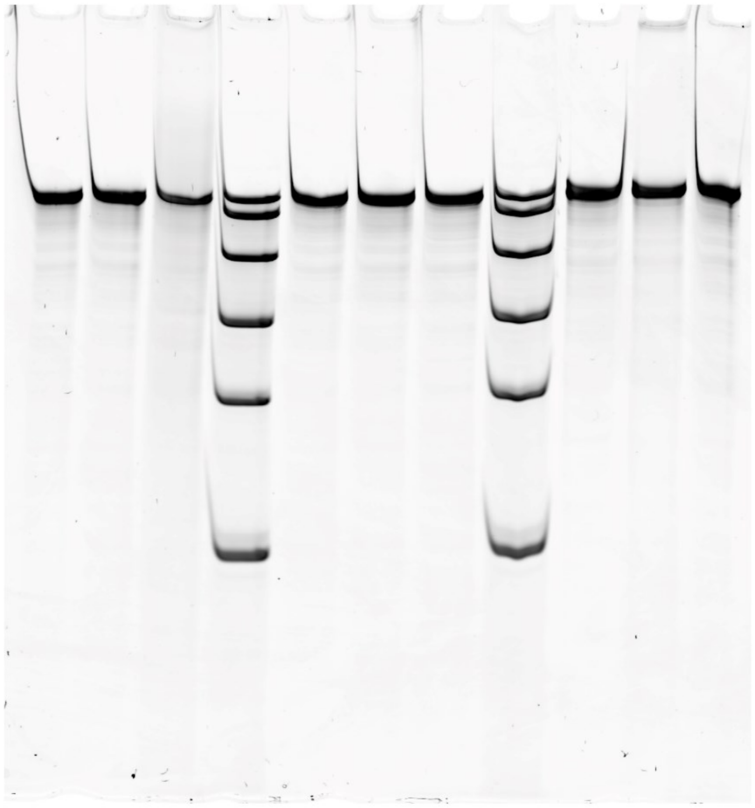

Figure S19 Denaturing 7.5% polyacrylamide-gel electrophoresis of cap 0-, TCO- and ApppG-capped GLuc-mRNA. RiboRuler, low range (Thermofisher) was used as marker. Abbreviations: TCO: TCO-cap, M1, M2: Marker

5.12 Microscopy images

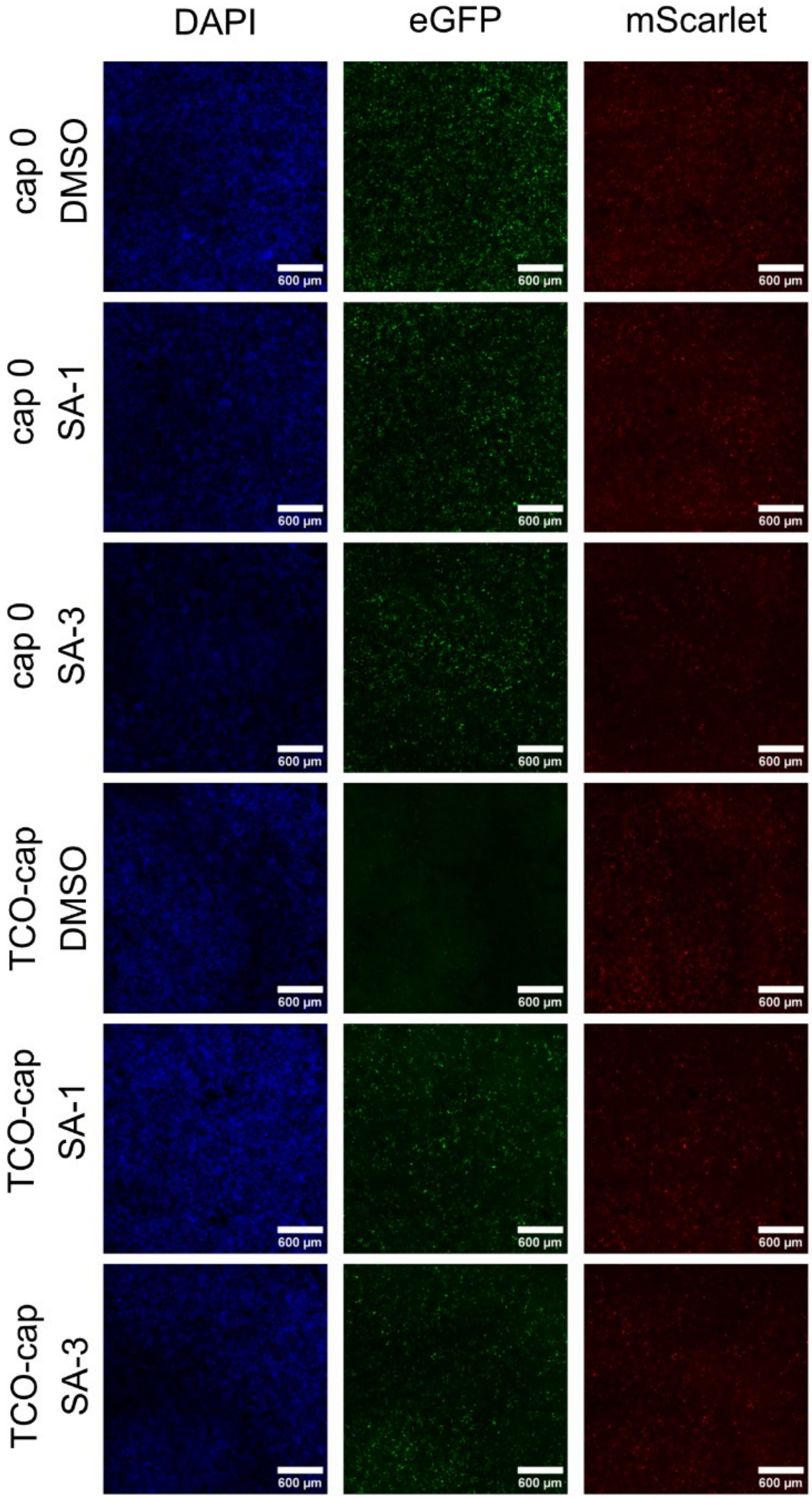

Figure S20 Microscopy images (10x) of cells that were co-transfected with differently capped mRNA (eGFP) and cap0-mScarlet-mRNA as transfection control and then treated with 10 µM tetrazines SA-1/SA-3 or DMSO as control for 1 h. Replicate 1.

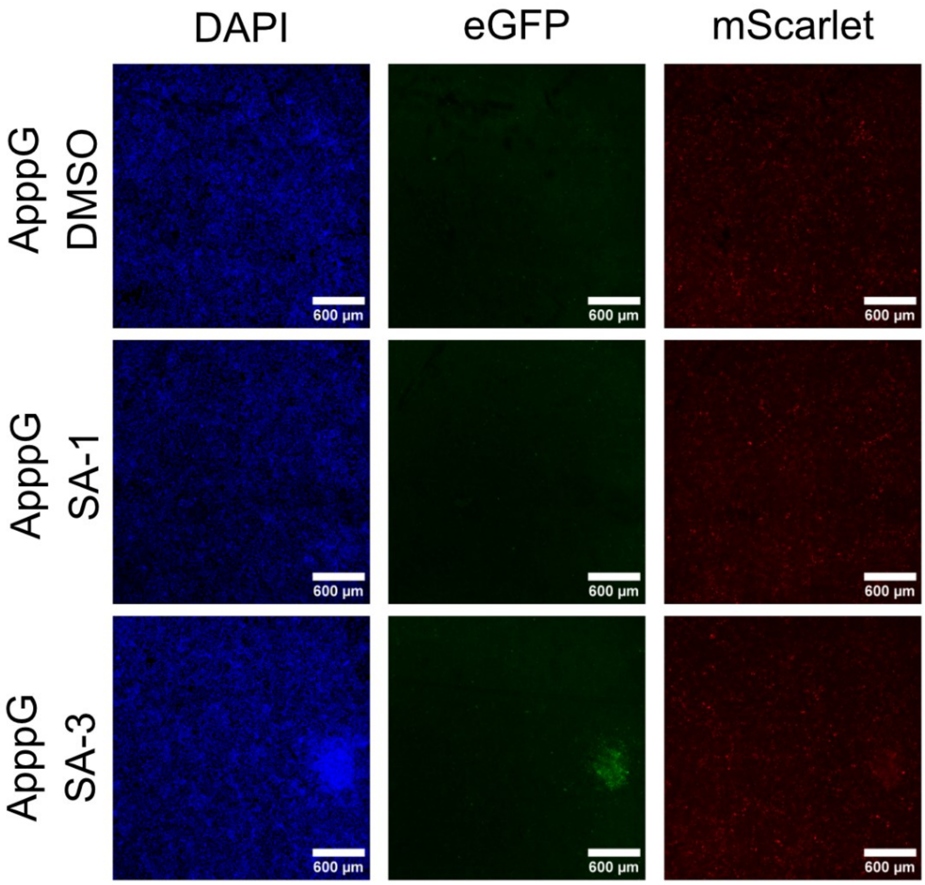

Figure S20 (continued)

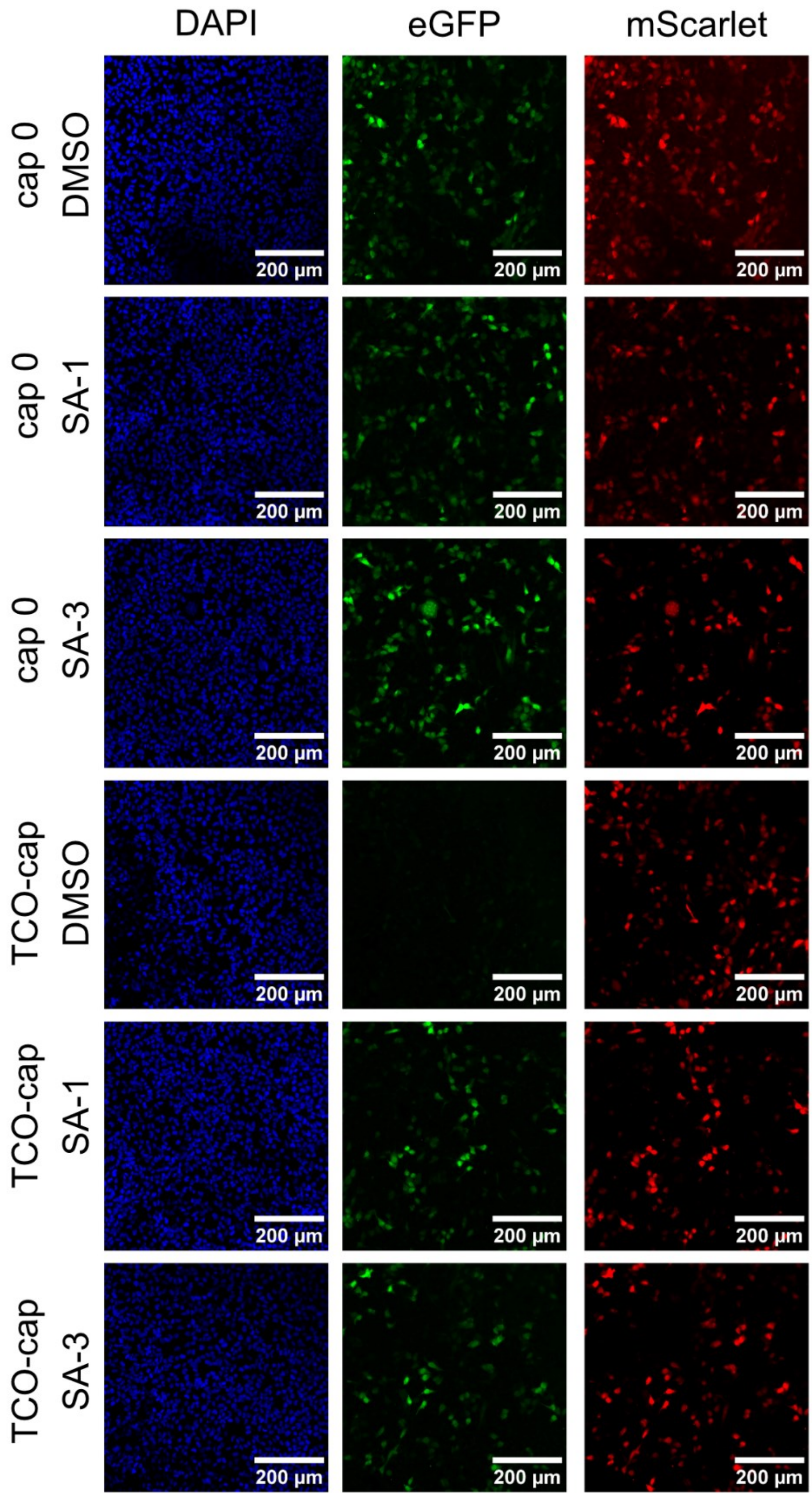

Figure S21 Microscopy images (40x) of cells that were co-transfected with differently capped mRNA (eGFP) and cap0-mScarlet-mRNA as transfection control and then treated with 10 µM tetrazines SA-1/SA-3 or DMSO as control for 1 h. Replicate 1.

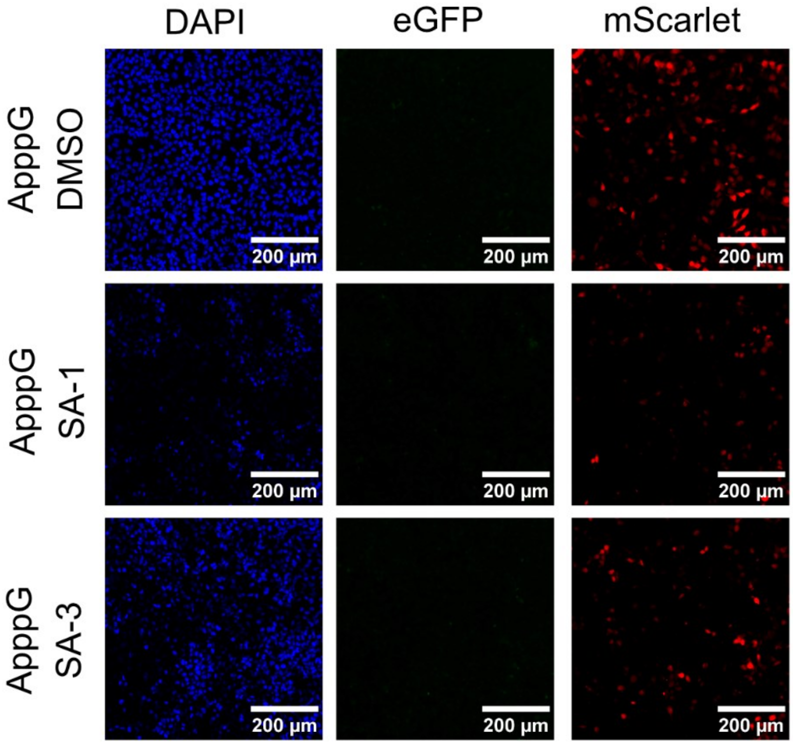

Figure S21 (continued)

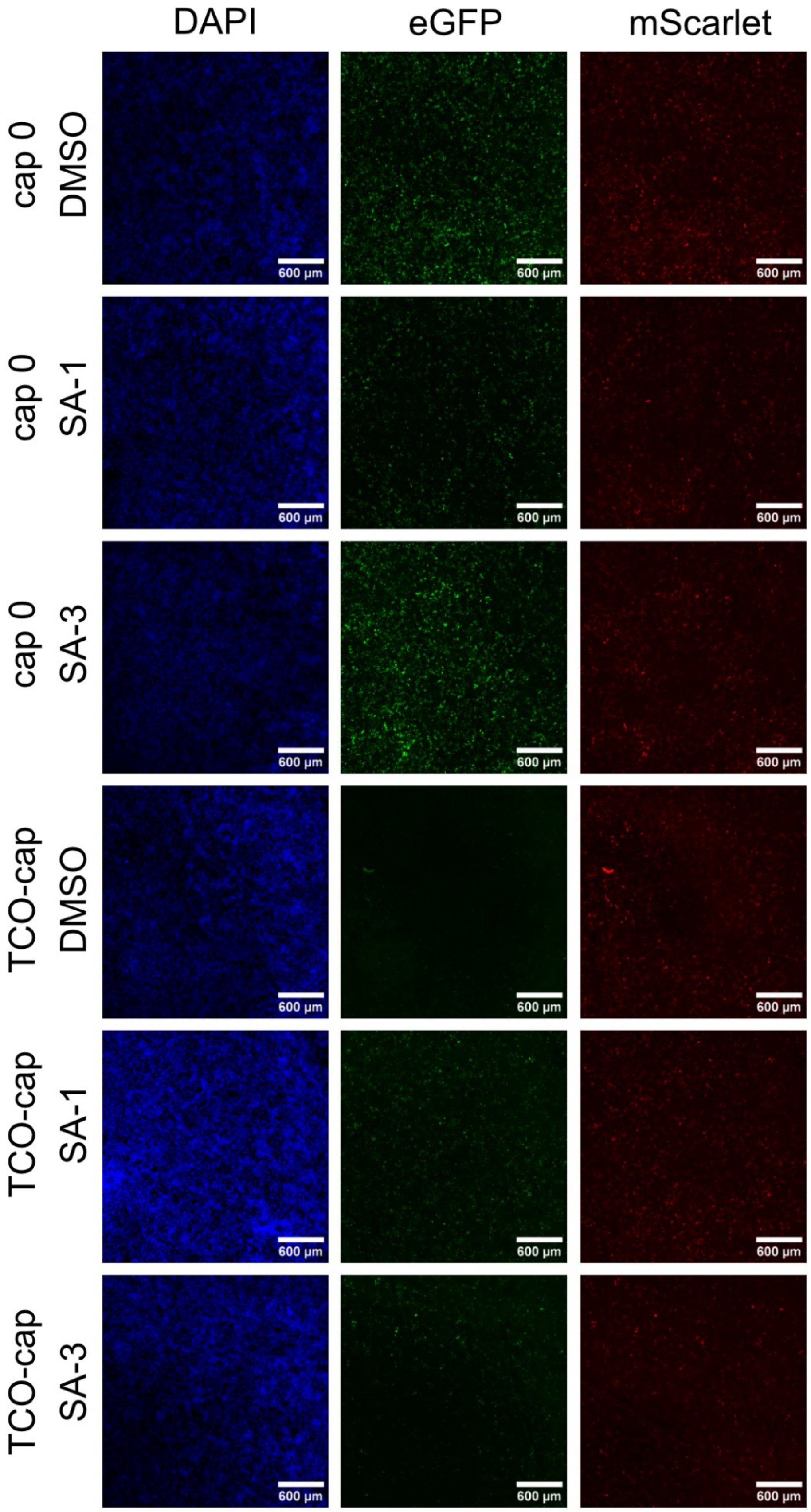

Figure S22 Microscopy images (10x) of cells that were co-transfected with differently capped mRNA (eGFP) and cap0-mScarlet-mRNA as transfection control and then treated with 10 µM tetrazines SA-1/SA-3 or DMSO as control for 1 h. Replicate 2.

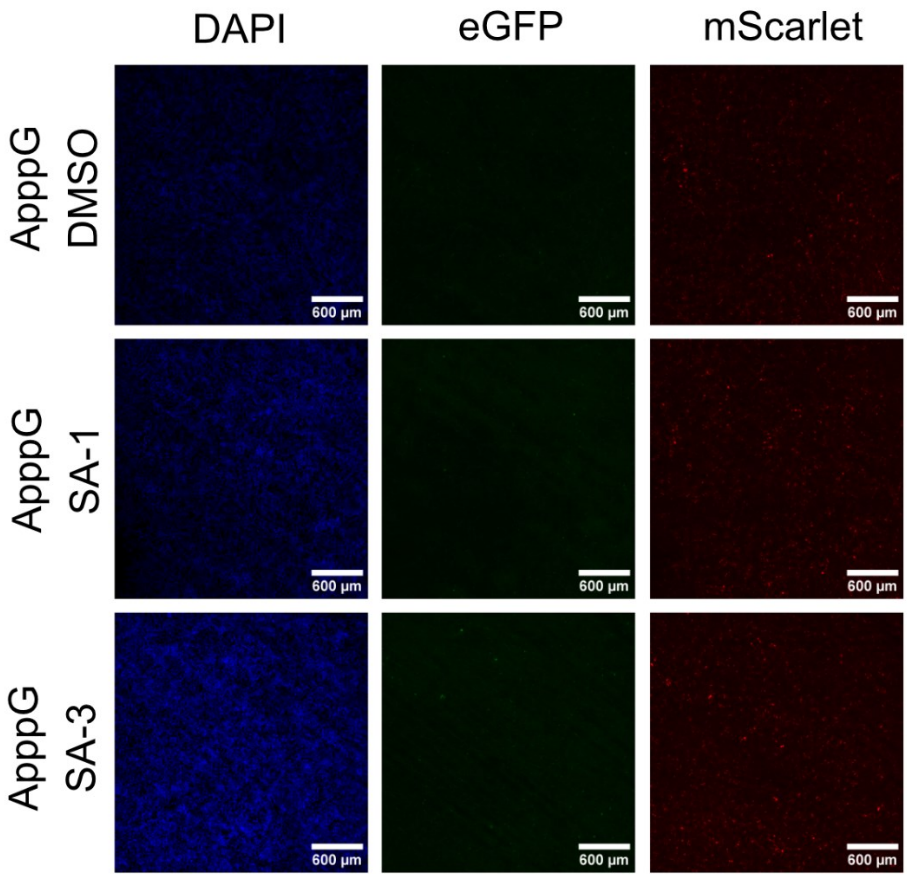

Figure S22 (continued)

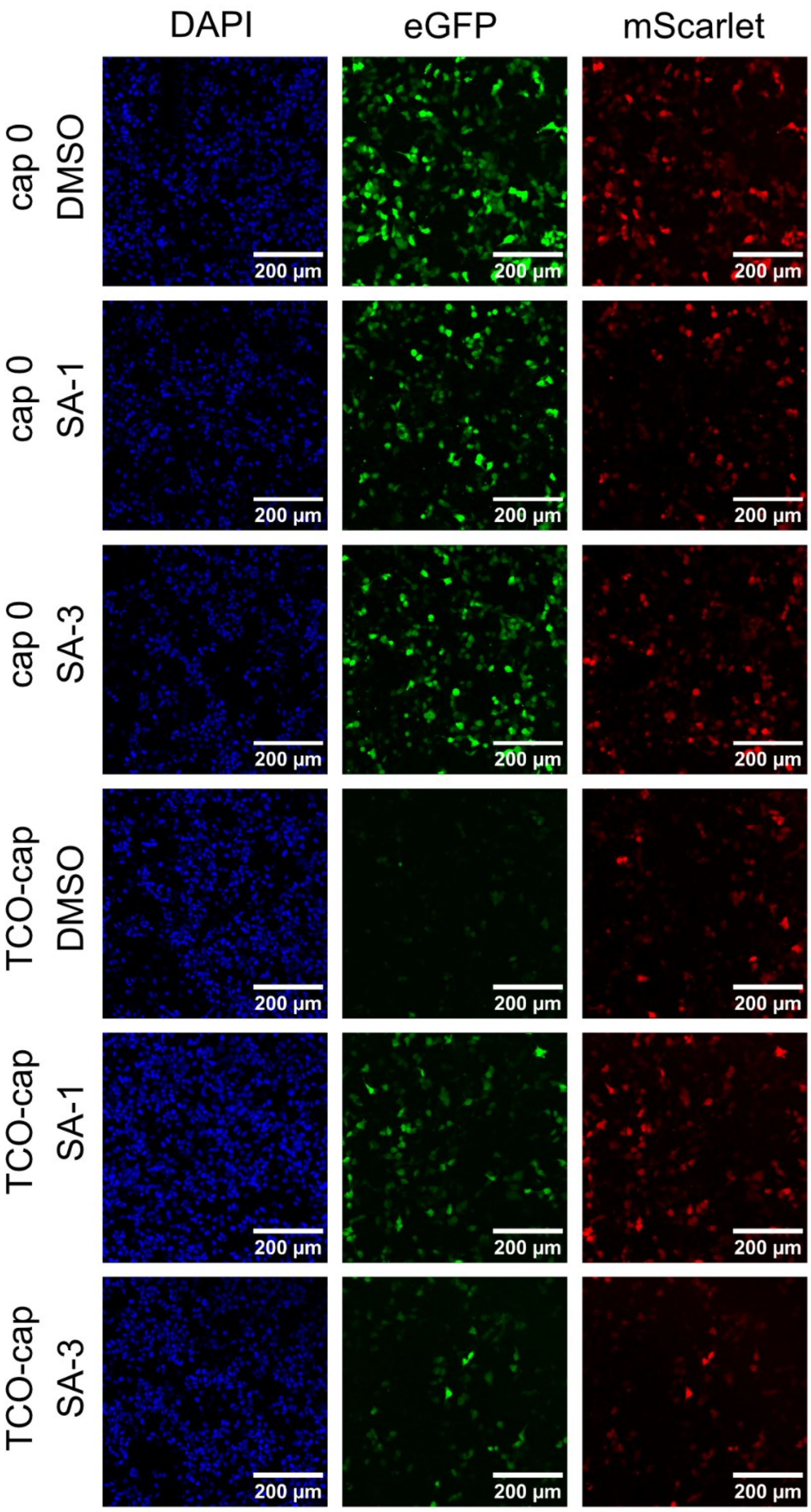

Figure S23 Microscopy images (40x) of cells that were co-transfected with differently capped mRNA (eGFP) and cap0-mScarlet-mRNA as transfection control and then treated with 10 µM tetrazines SA-1/SA-3 or DMSO as control for 1 h. Replicate 2.

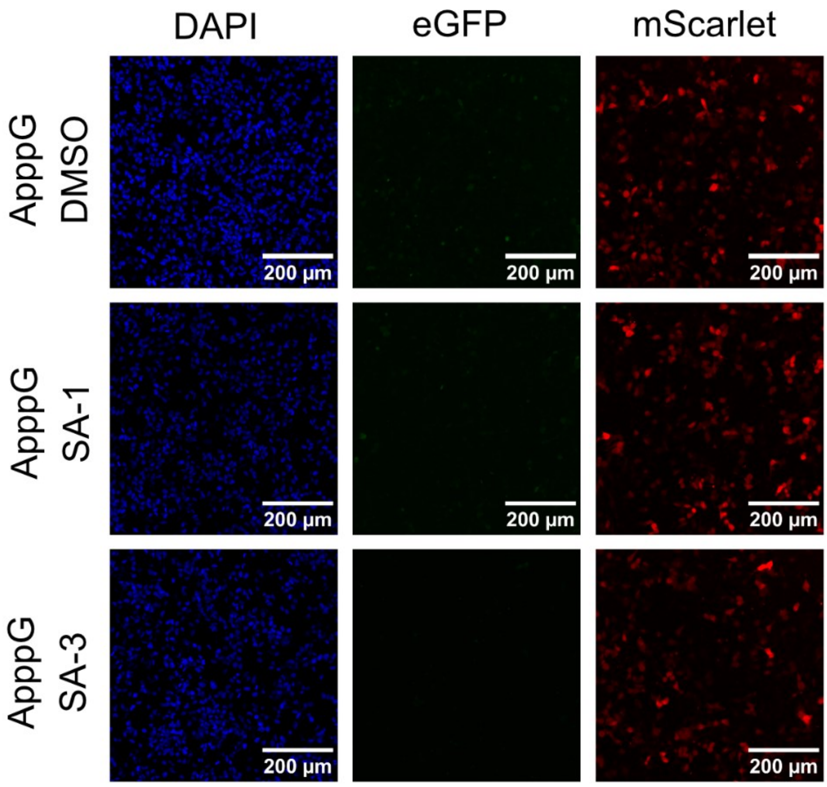

Figure S23 (continued)

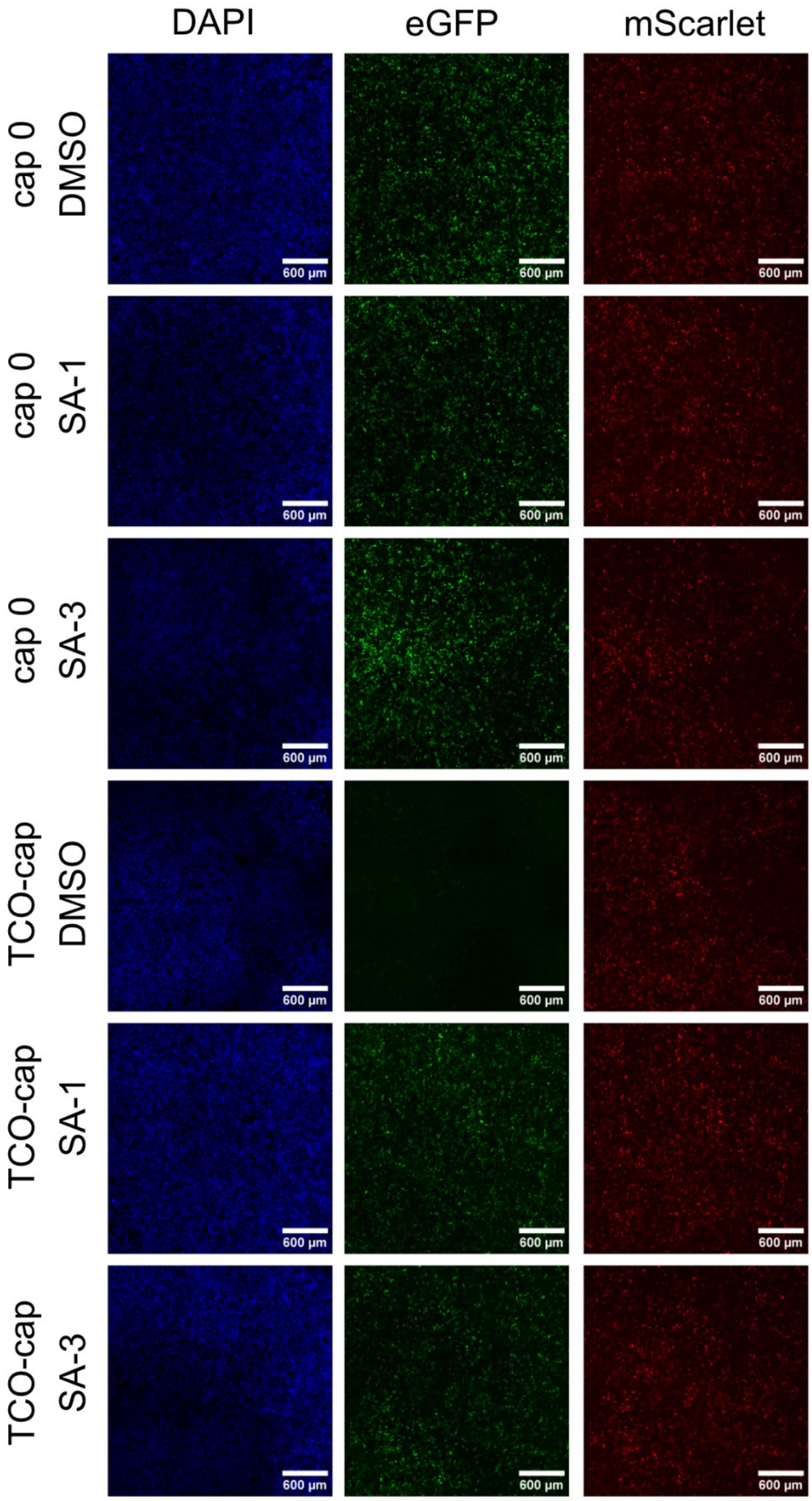

Figure S24 Microscopy images (10x) of cells that were co-transfected with differently capped mRNA (eGFP) and cap0-mScarlet-mRNA as transfection control and then treated with 10 µM tetrazines SA-1/SA-3 or DMSO as control for 1 h. Replicate 3.

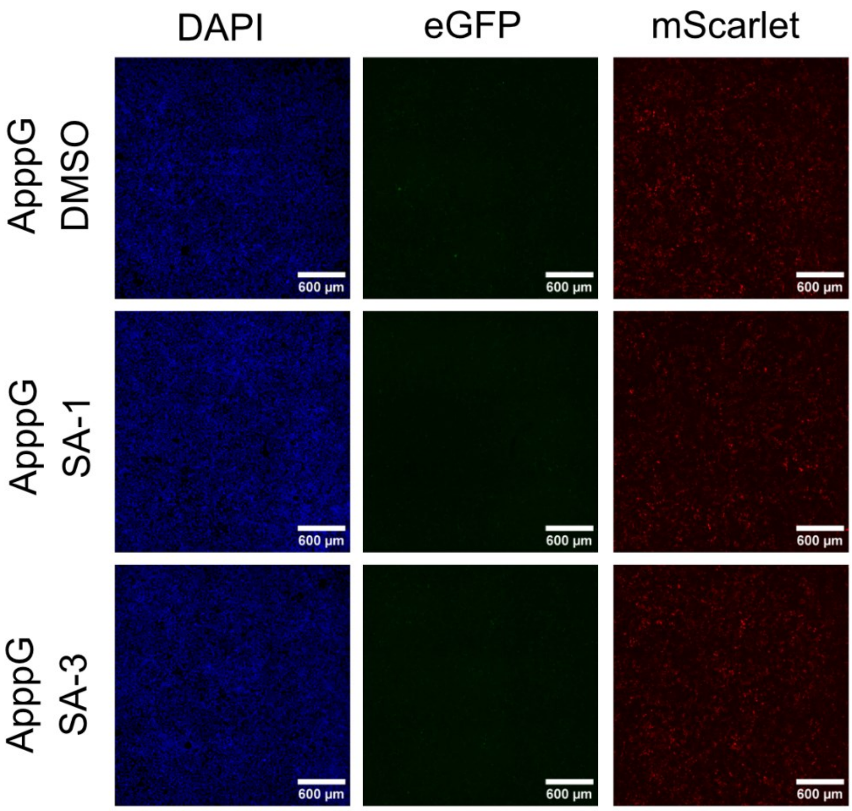

Figure S24 (continued)

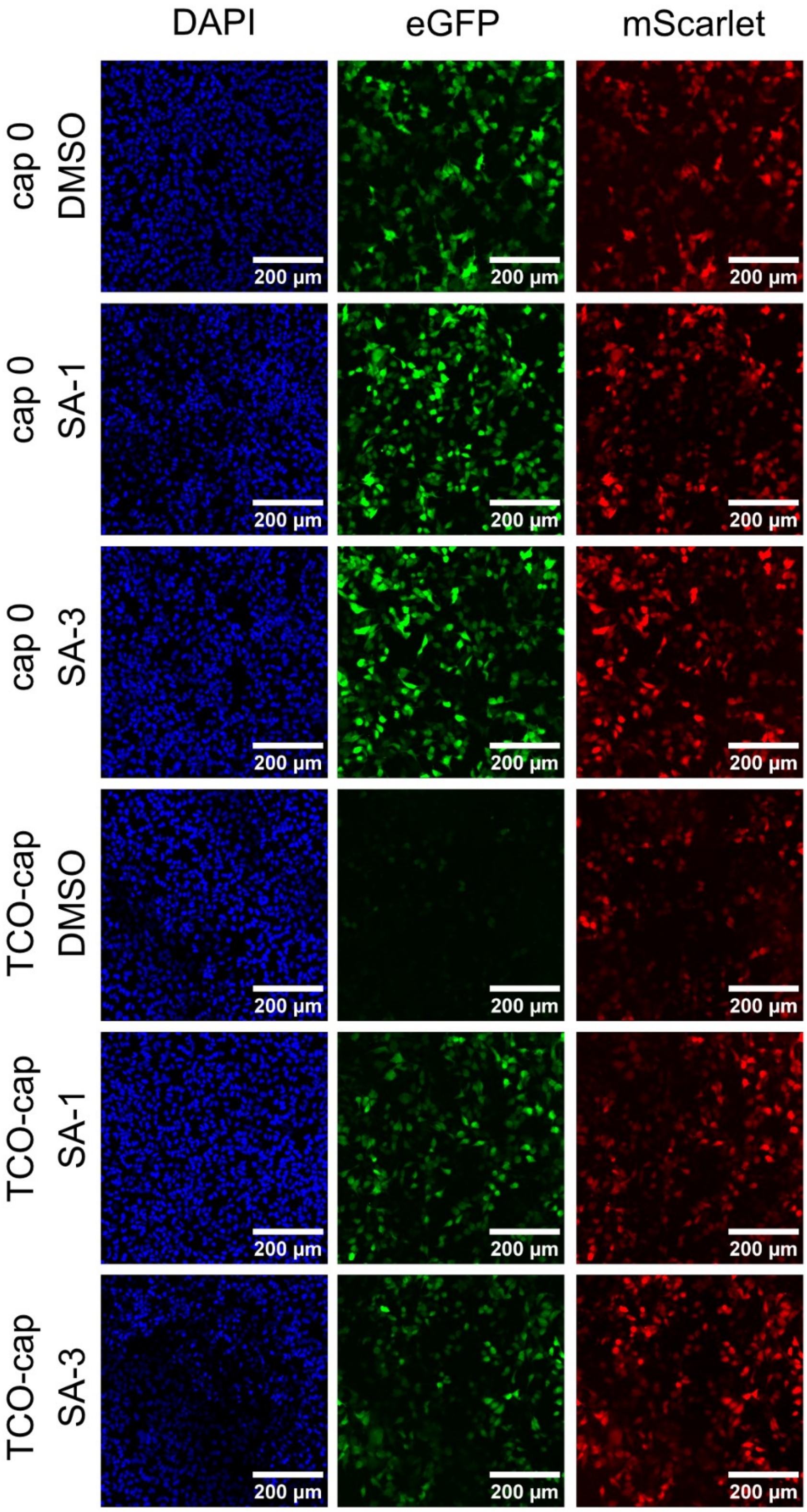

Figure S25 Microscopy images (40x) of cells that were co-transfected with differently capped mRNA (eGFP) and cap0-mScarlet-mRNA as transfection control and then treated with 10 µM tetrazines SA-1/SA-3 or DMSO as control for 1 h. Replicate 3.

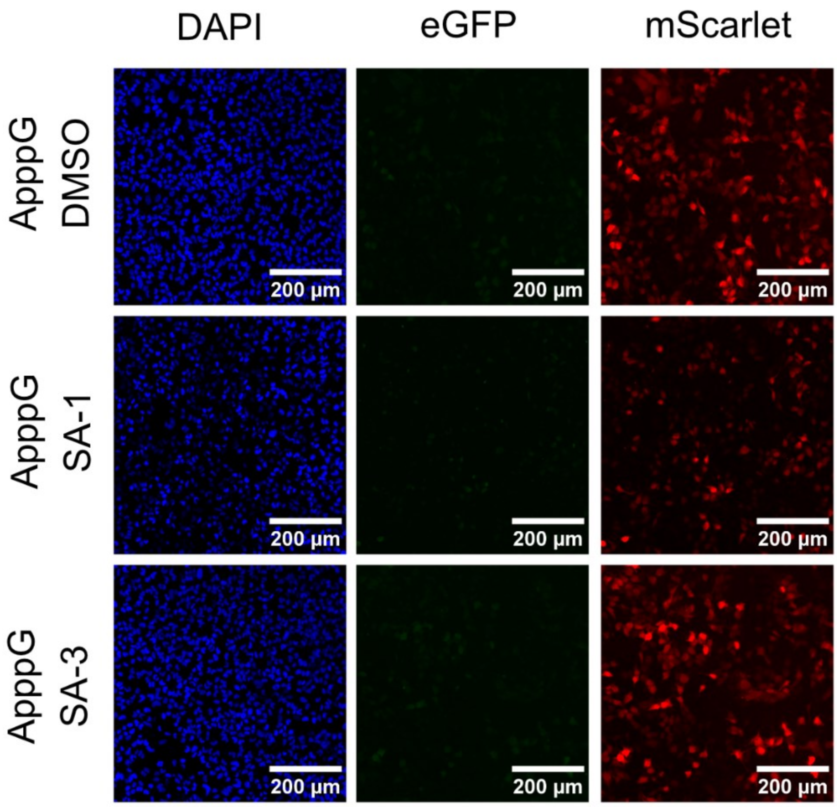

Figure S25 (continued)

5.13 HPLC analysis

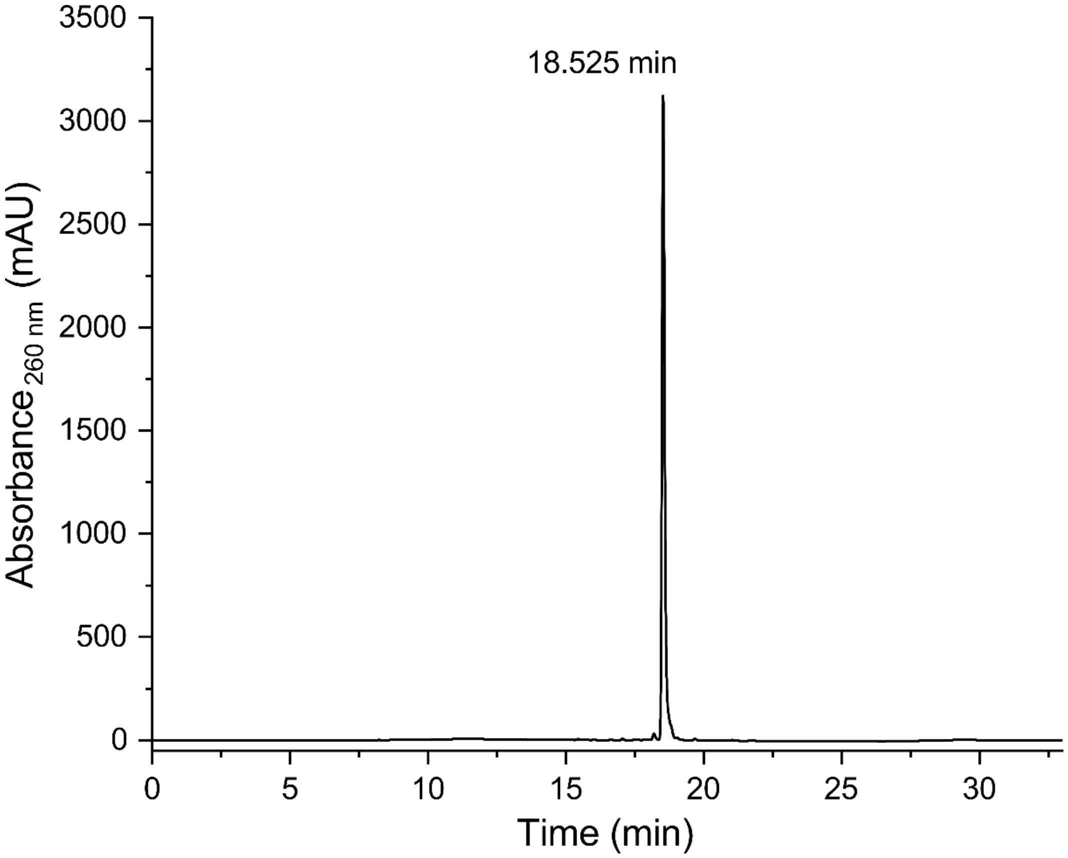

Figure S26: Characterization of TCO-GMP **2** by means of HPLC.

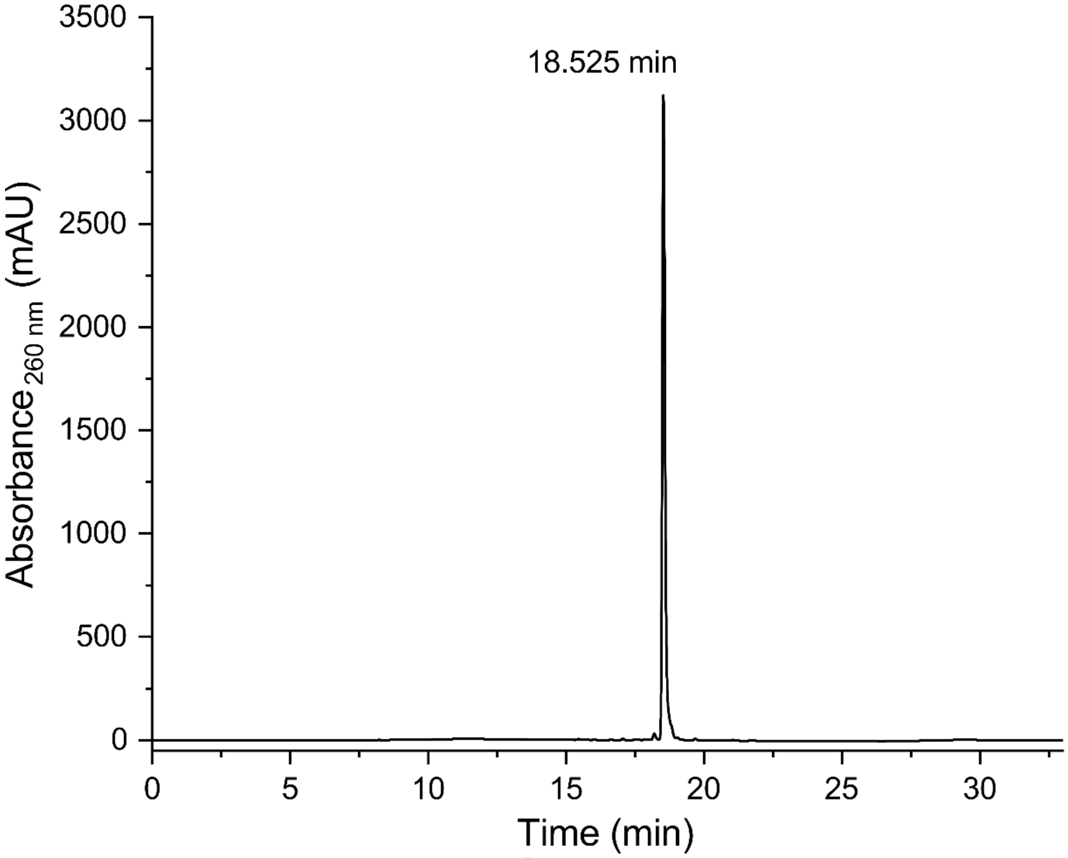

Figure S27: Characterization of TCO-m^{7}GMP **3** by means of HPLC.

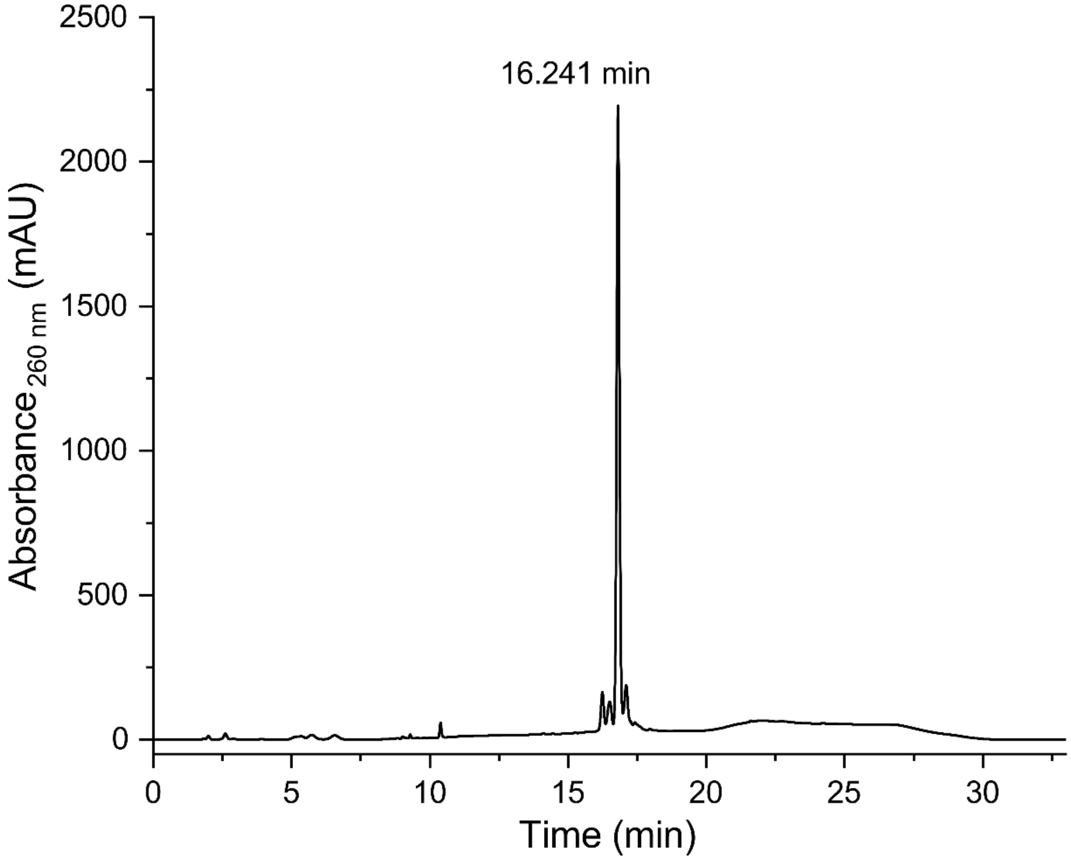

Figure S28: Characterization of the TCO-cap **4** by means of HPLC.

5.14 HRMS spectra

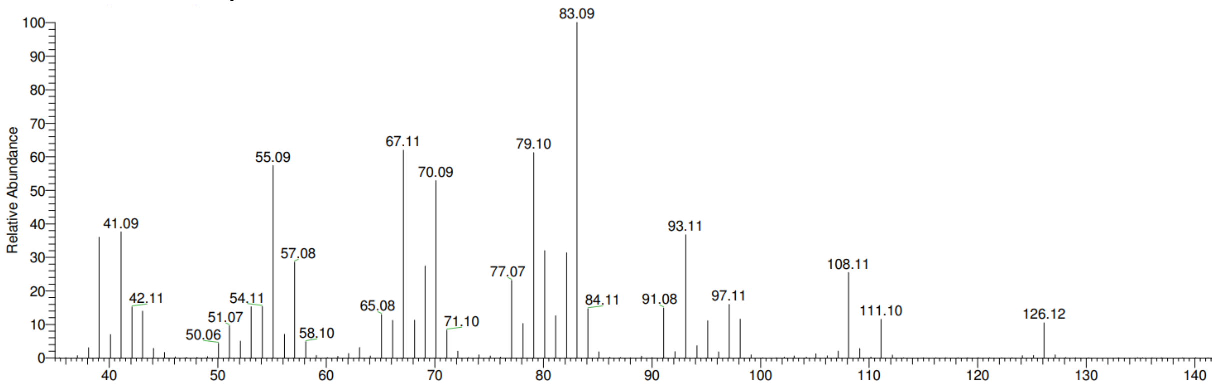

Figure S29: Mass spectrum of *cis*-cyclooctenol measured on an ISQ 7000 GC/MS with EI ionisation; $C_8H_{14}O$ observed as $[M]^+$ at *m/z* = 126.12.

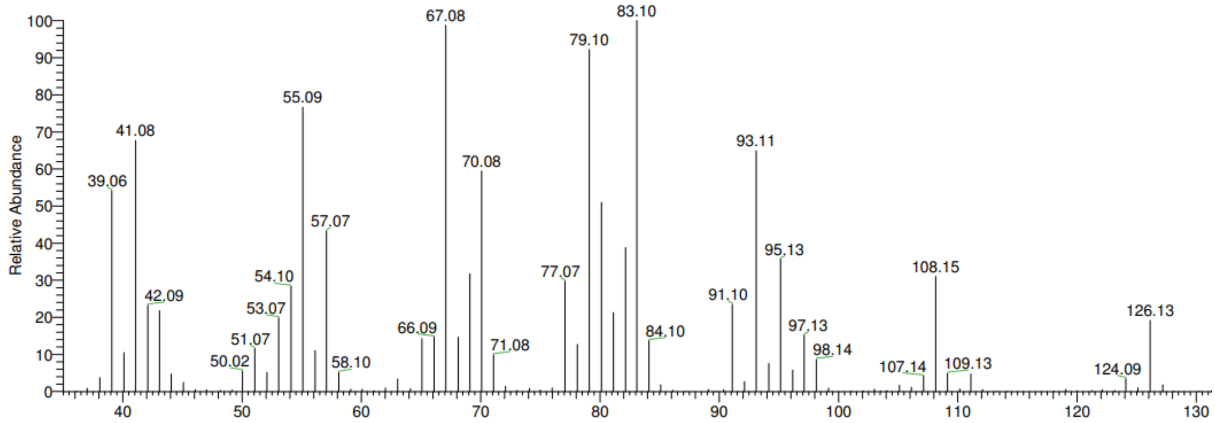

Figure S30: Mass spectrum of axial *trans*-cyclooctenol measured on an ISQ 7000 GC/MS with EI ionisation; $C_8H_{14}O$ observed as $[M]^+$ at *m/z* = 126.13.

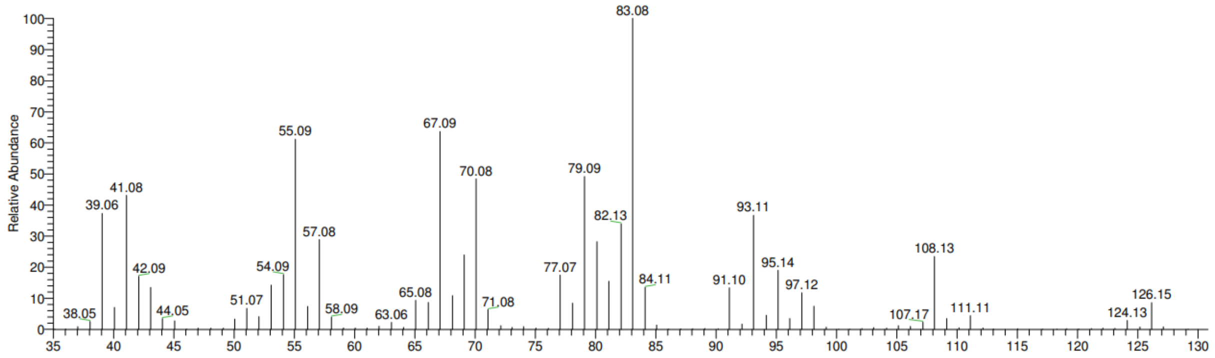

Figure S31: Mass spectrum of equatorial *trans*-cyclooctenol measured on an ISQ 7000 GC/MS with EI ionisation; $C_8H_{14}O$ observed as $[M]^+$ at *m/z* = 126.15.

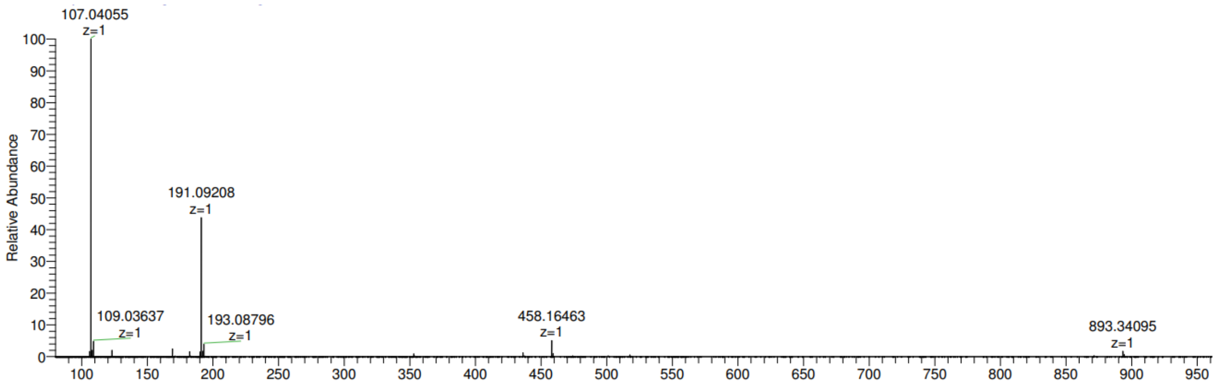

Figure S32: HRMS spectrum of TCO-guanosine **1** measured on a LTQ Orbitrap XL with ESI+ ionisation; $C_{19}H_{25}N_5O_7$ observed as $[M+Na]^+$ at *m/z* = 458.16463.

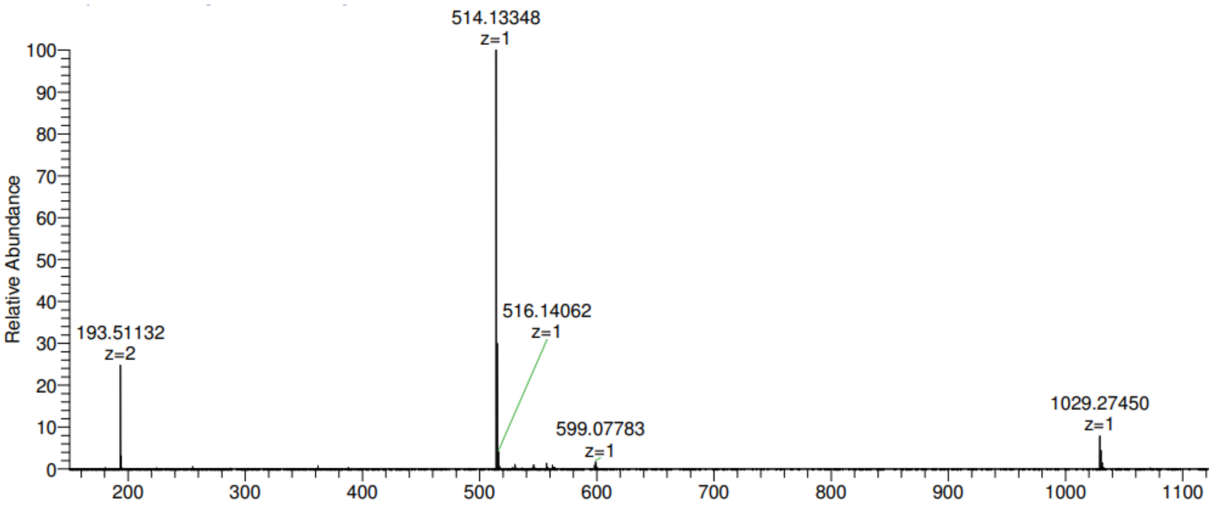

Figure S33: HRMS spectrum of TCO-GMP **2** measured on a LTQ Orbitrap XL with ESI- ionisation; $C_{19}H_{24}N_5O_{10}P^{2-}$ observed as $[M+H]^-$ at *m/z* = 514.13348.

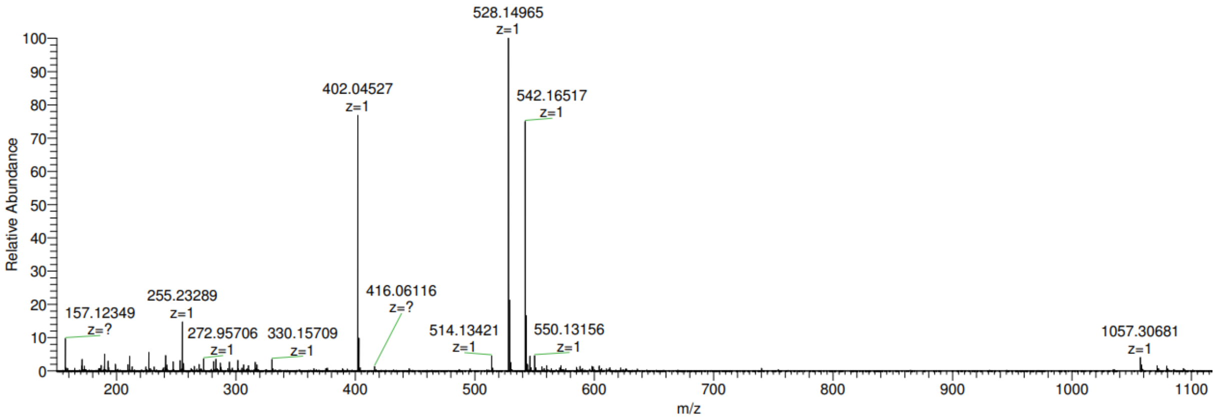

Figure S34: HRMS spectrum of TCO-m⁷GMP **3** measured on a LTQ Orbitrap XL with ESI- ionisation; $C_{20}H_{27}N_5O_{10}P^-$ observed as $[M]^-$ at *m/z* = 528.14965.

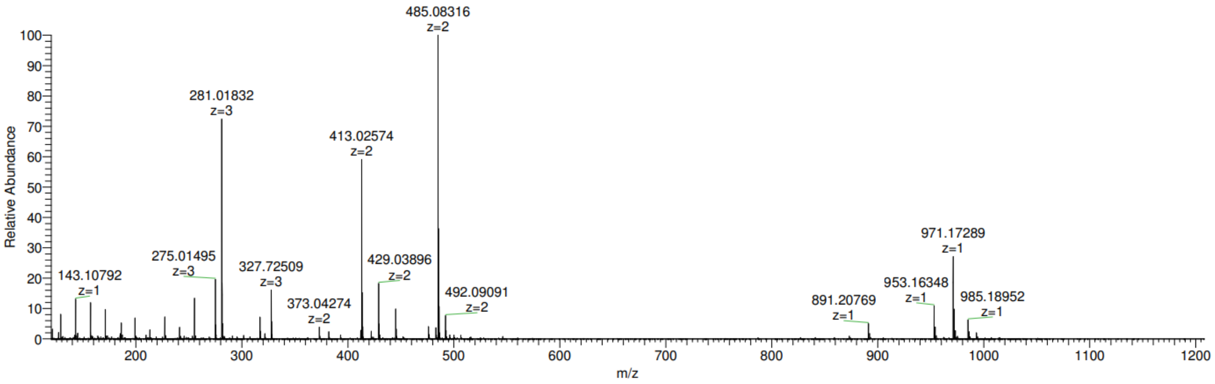

Figure S35: HRMS spectrum of the TCO-cap **4** measured on a LTQ Orbitrap XL with ESI-ionisation; $C_{30}H_{39}N_{10}O_{20}P_3^{2-}$ observed as [M+H]$^-$ at *m/z* = 953.16348.

5.15 NMR spectra

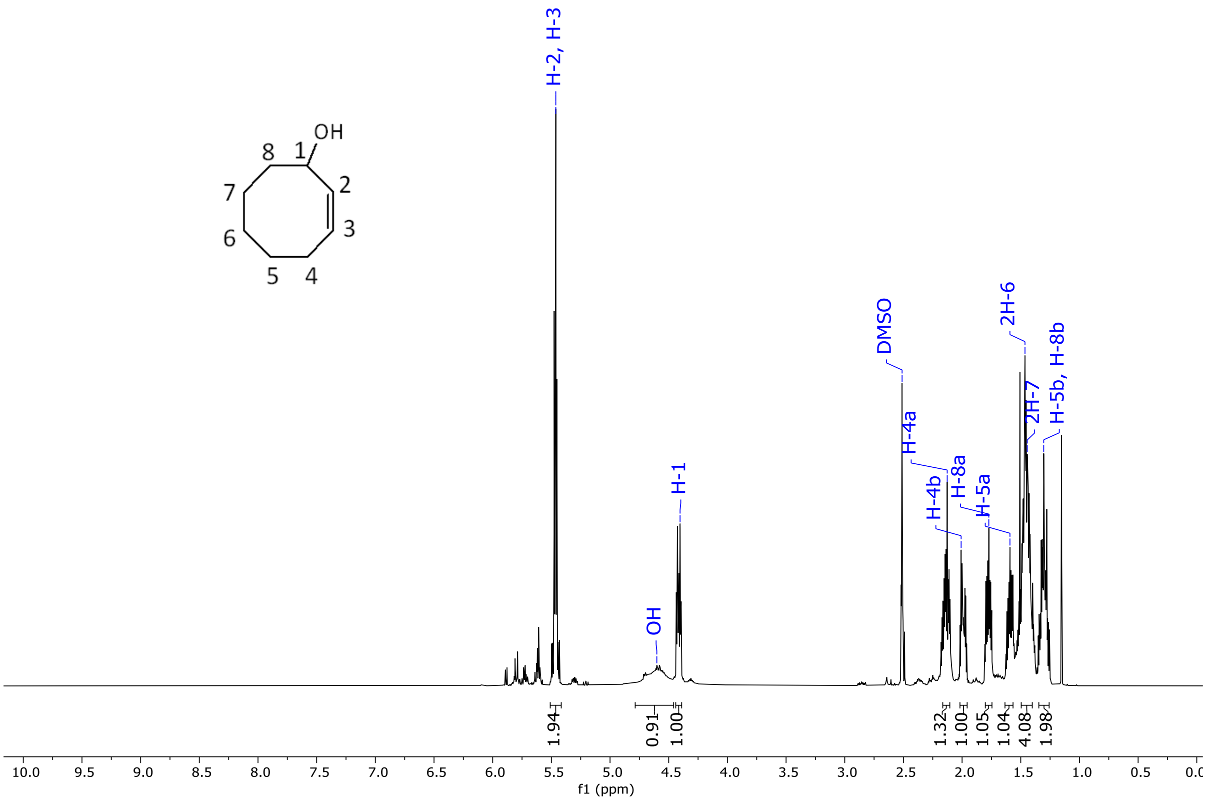

Figure S36: ^{1}H NMR of *cis*-cyclooctenol in DMSO-*d6*.

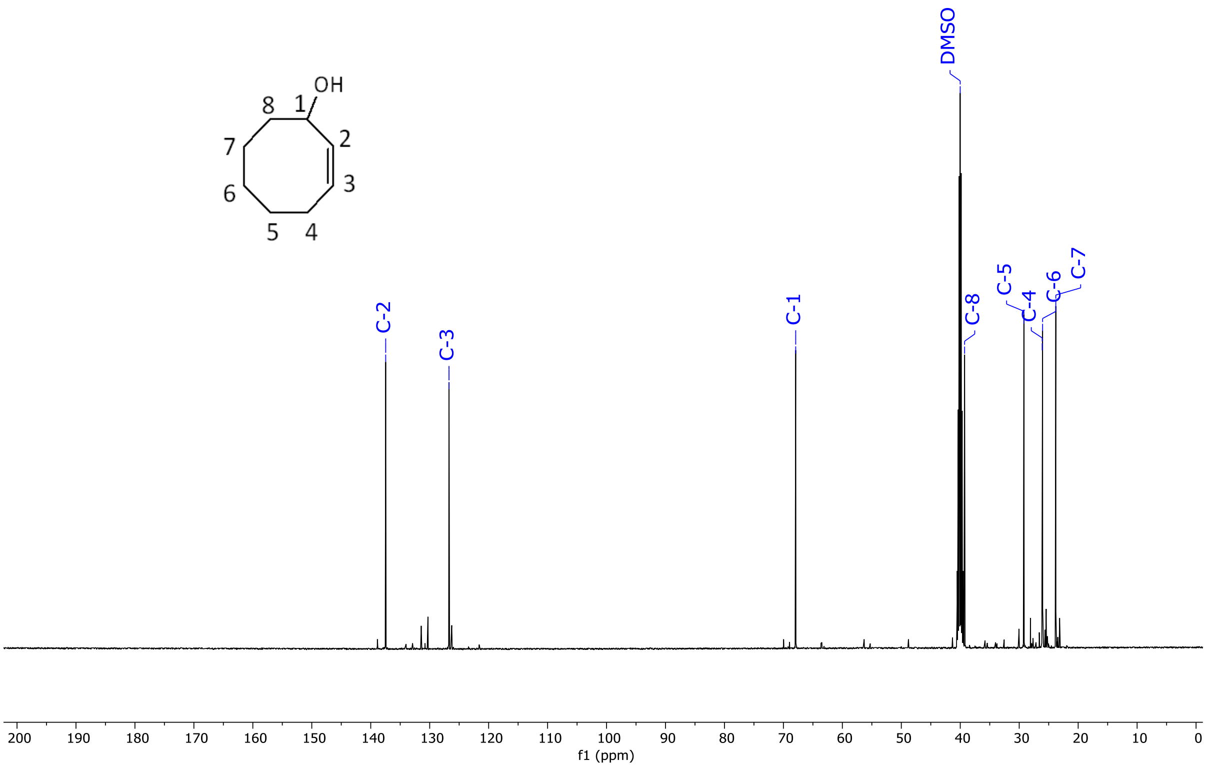

Figure S37: ^{13}C NMR of *cis*-cyclooctenol in DMSO-*d6*.

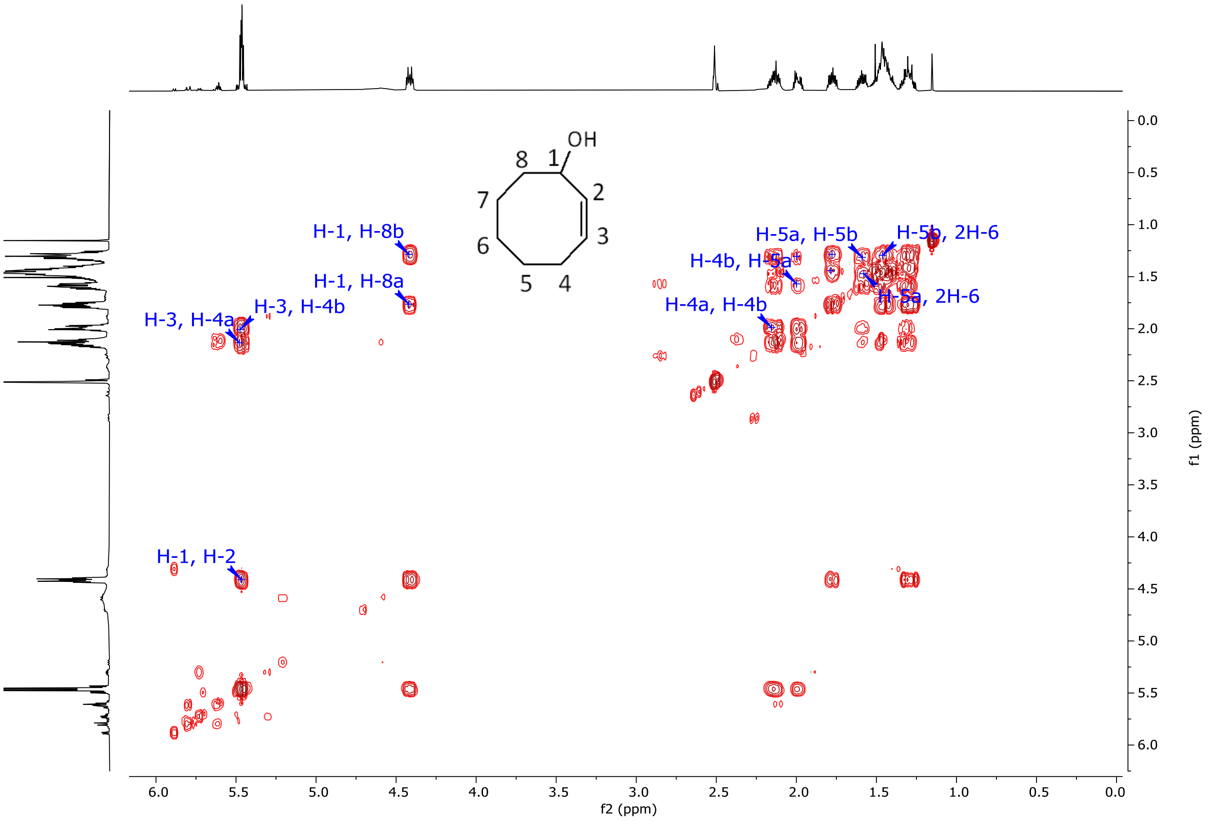

Figure S38: ^{1}H COSY spectrum of *cis*-cyclooctenol in DMSO-*d6*.

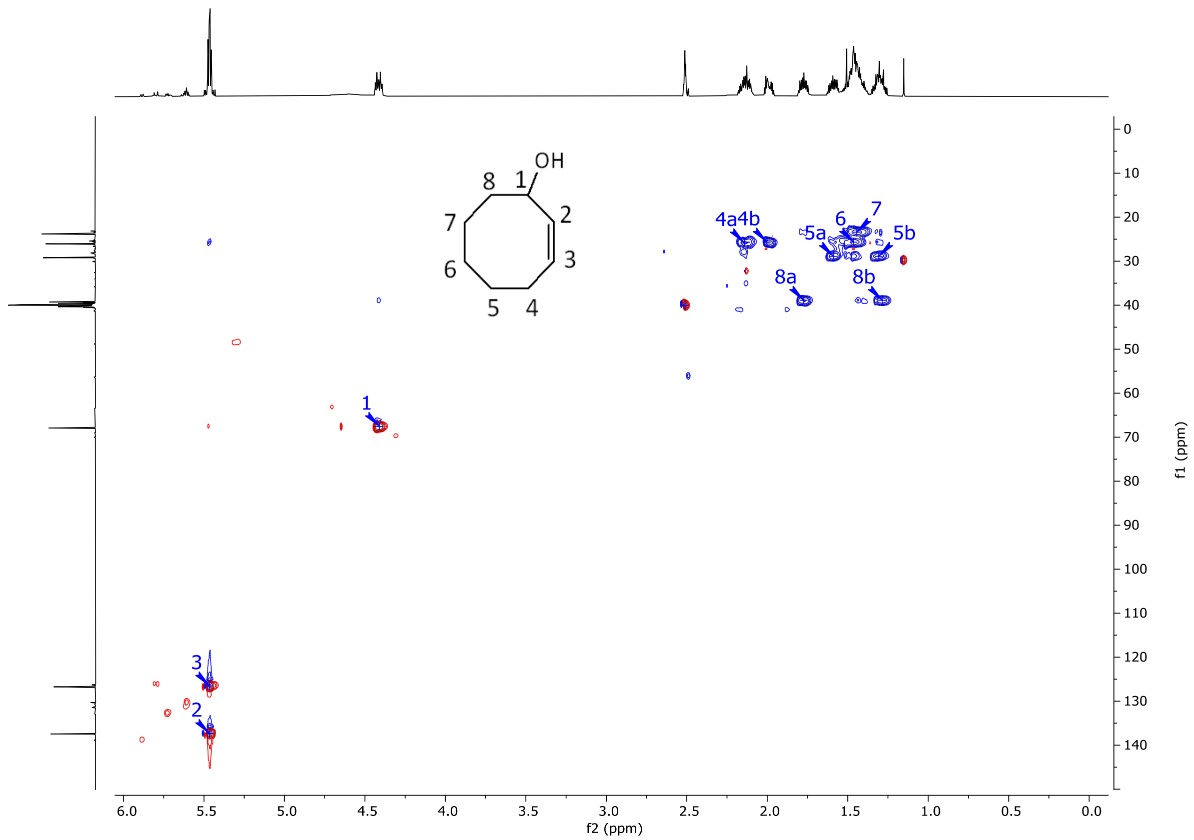

Figure S39: ^{1}H-^{13}C HSQC spectrum of *cis*-cyclooctenol in DMSO-*d6*.

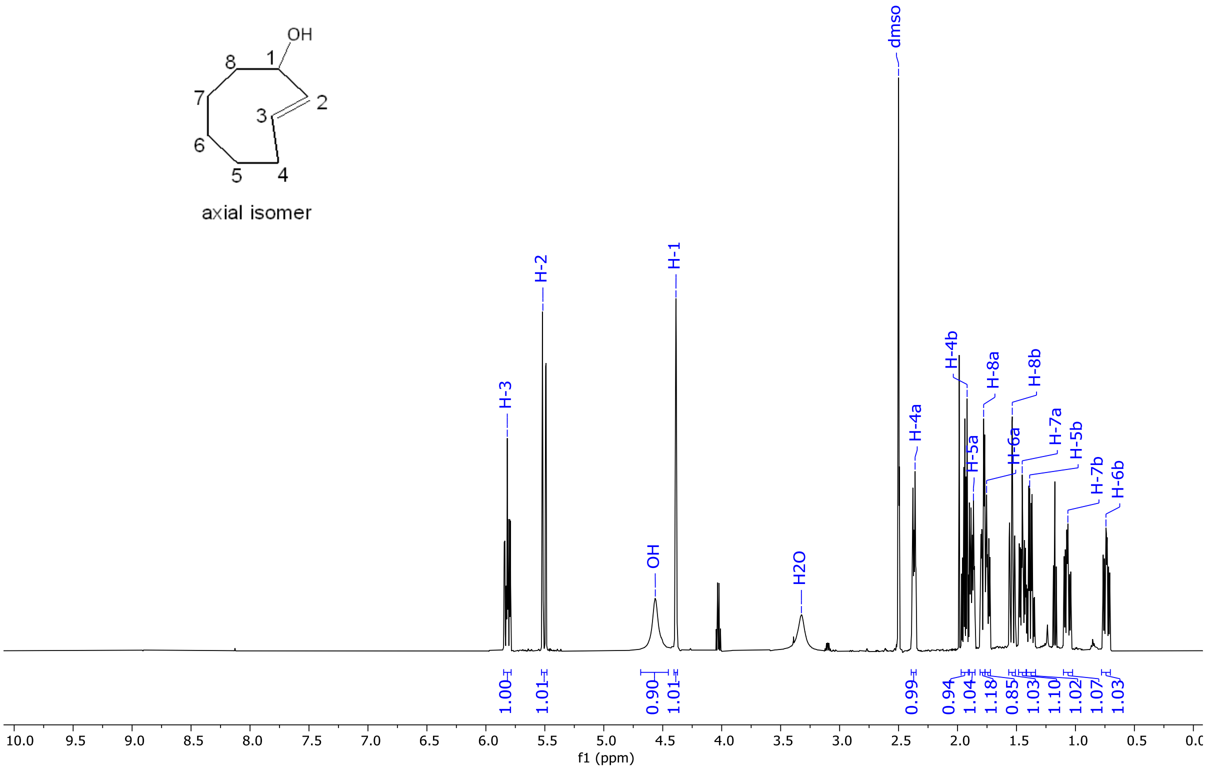

Figure S40: ^{1}H NMR of axial *trans*-cyclooctenol in DMSO-*d6*.

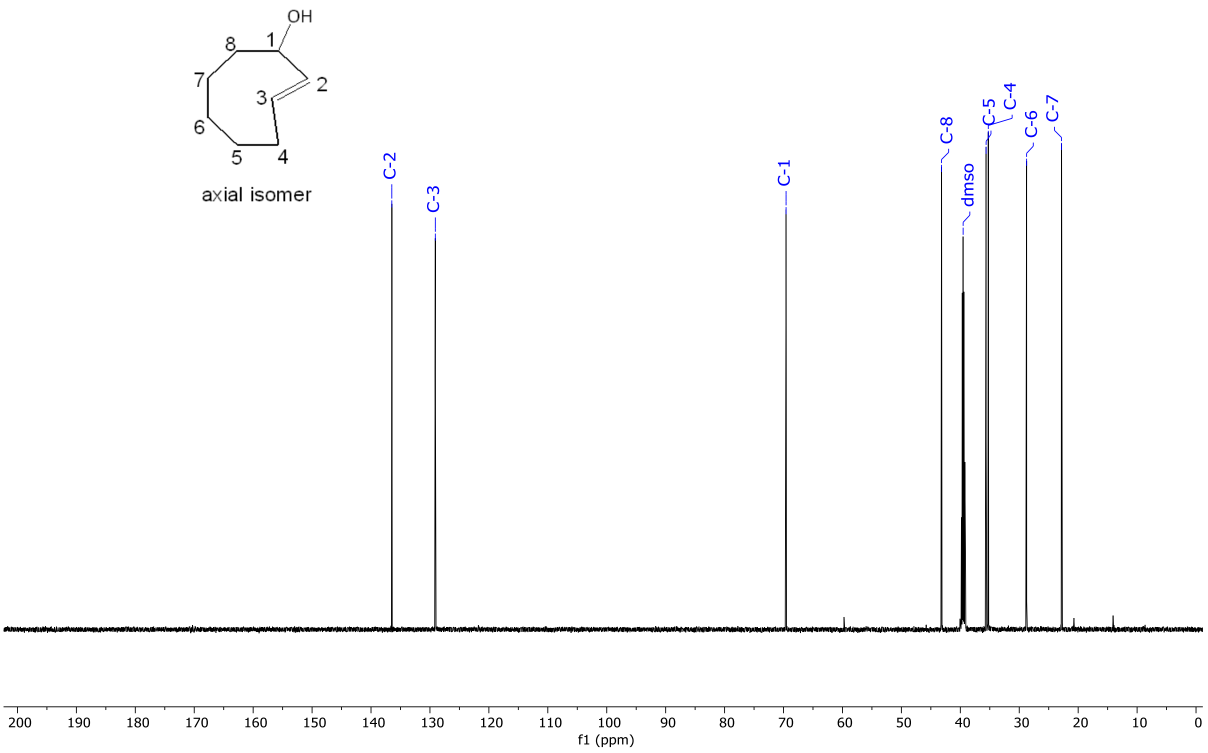

Figure S41: ^{13}C NMR of axial *trans*-cyclooctenol in DMSO-*d6*.

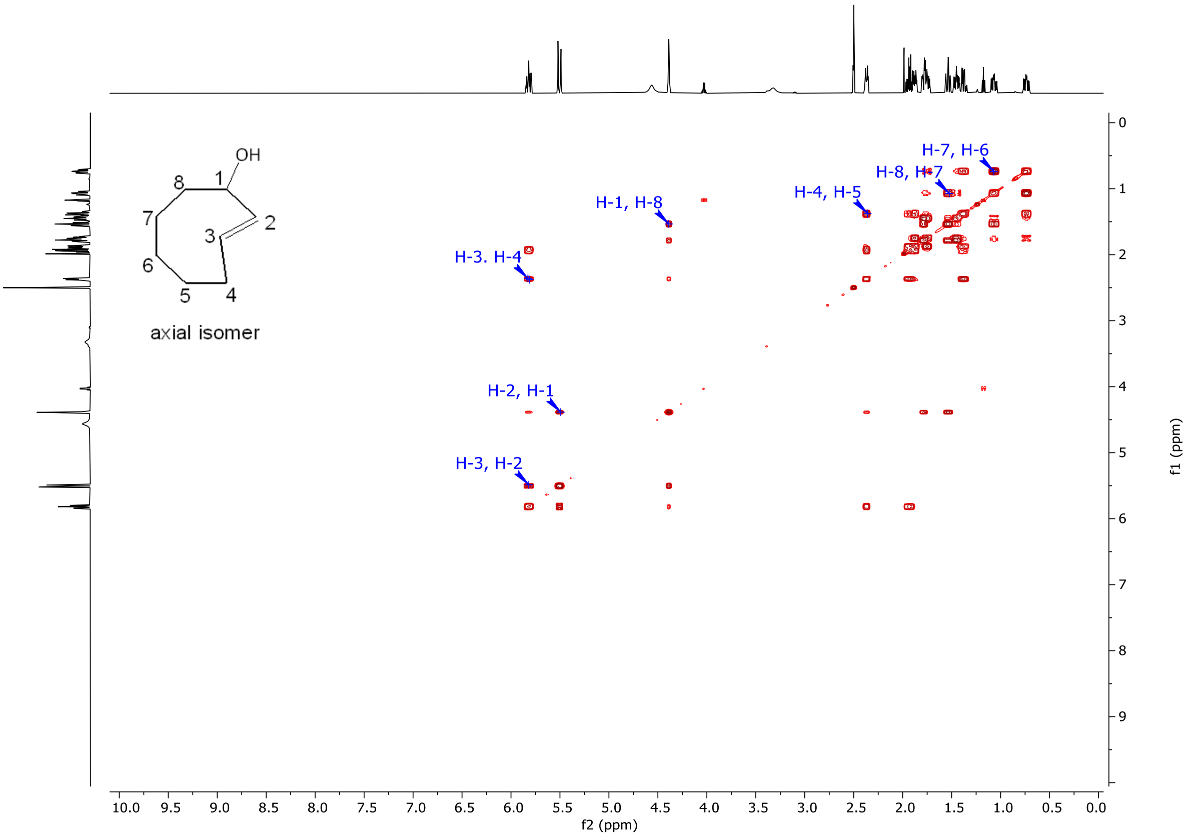

Figure S42: ^{1}H COSY spectrum of axial *trans*-cyclooctenol in DMSO-*d6*.

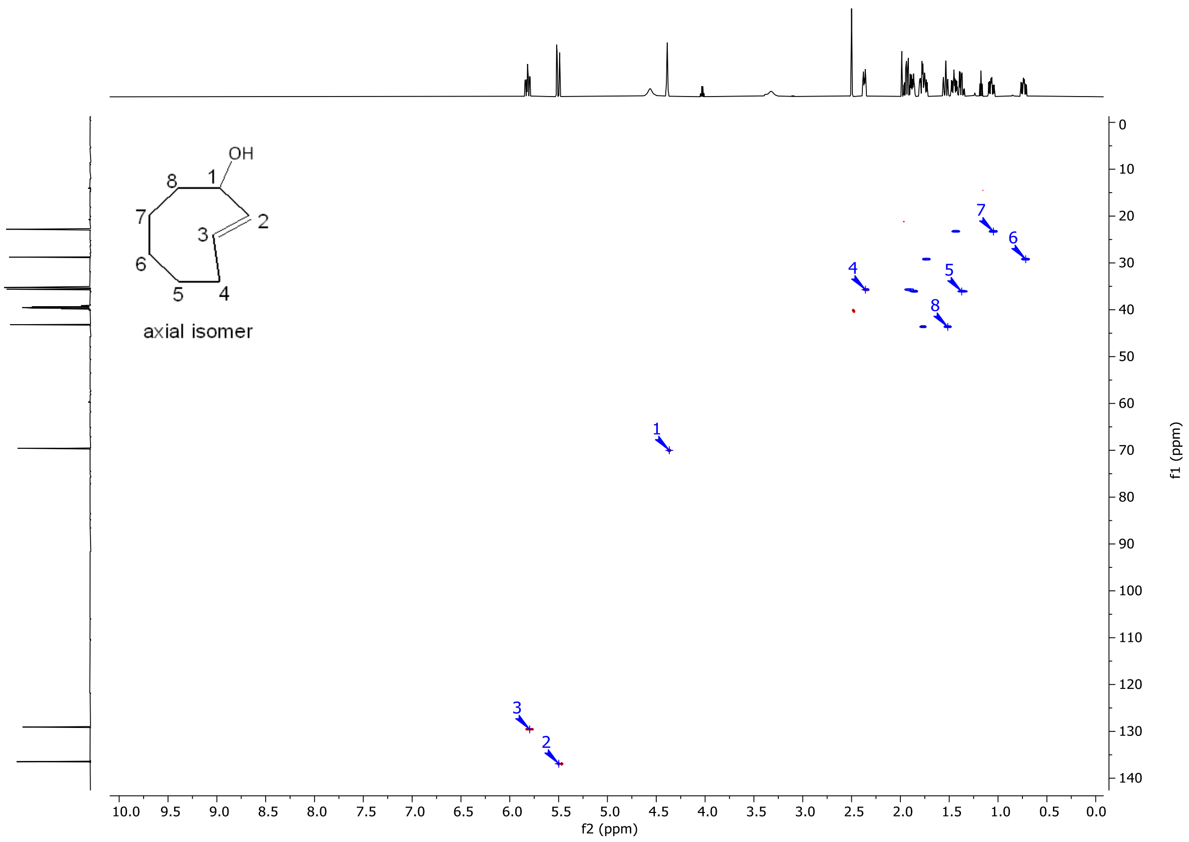

Figure S43: ^{1}H-^{13}C HSQC spectrum of axial *trans*-cyclooctenol in DMSO-*d6*.

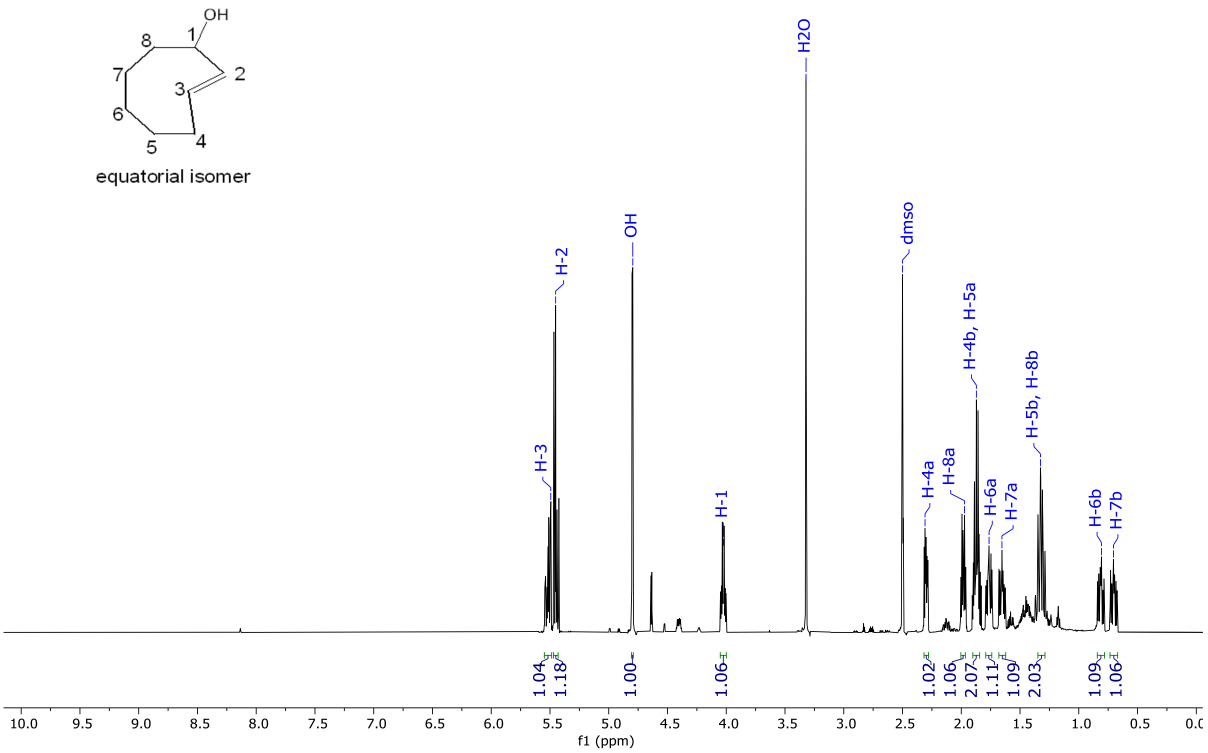

Figure S44: ^{1}H NMR of equatorial *trans*-cyclooctenol in DMSO-*d6*.

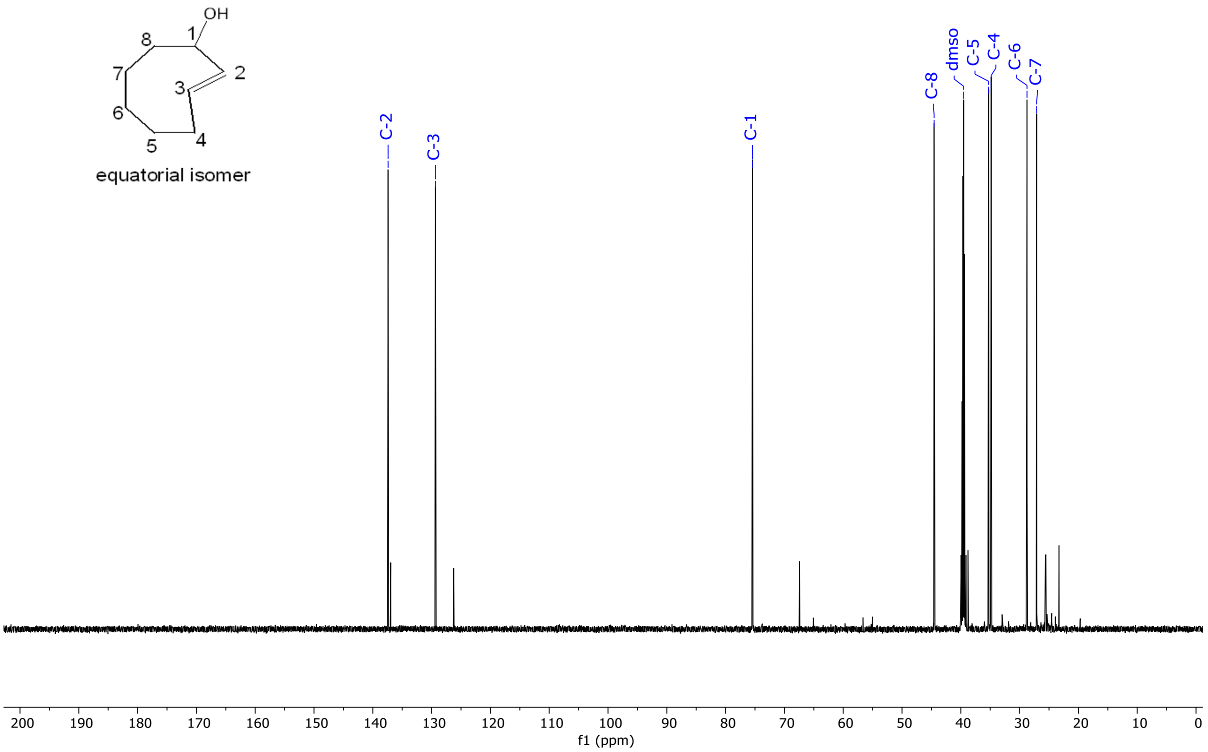

Figure S45: ^{13}C NMR of equatorial *trans*-cyclooctenol in DMSO-*d6*.

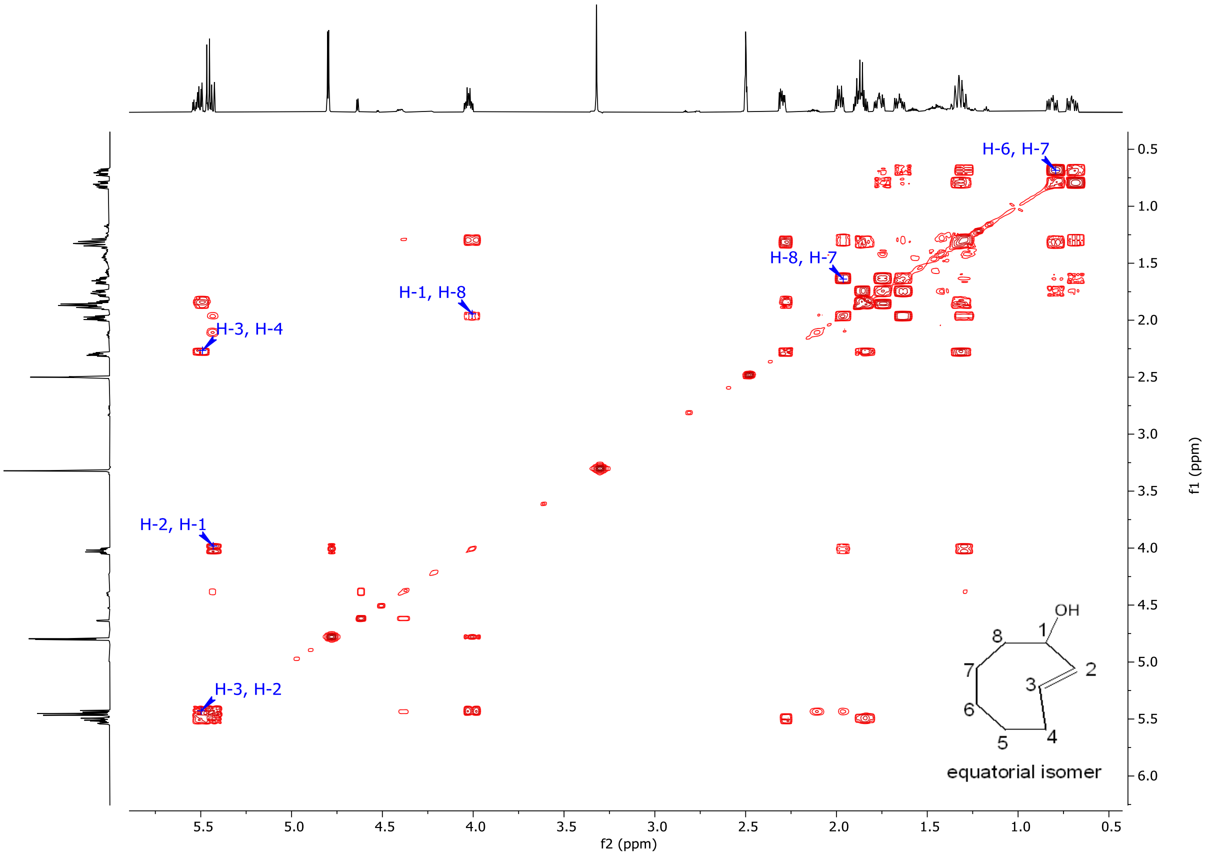

Figure S46: ^{1}H COSY spectrum of equatorial *trans*-cyclooctenol in DMSO-*d6*.

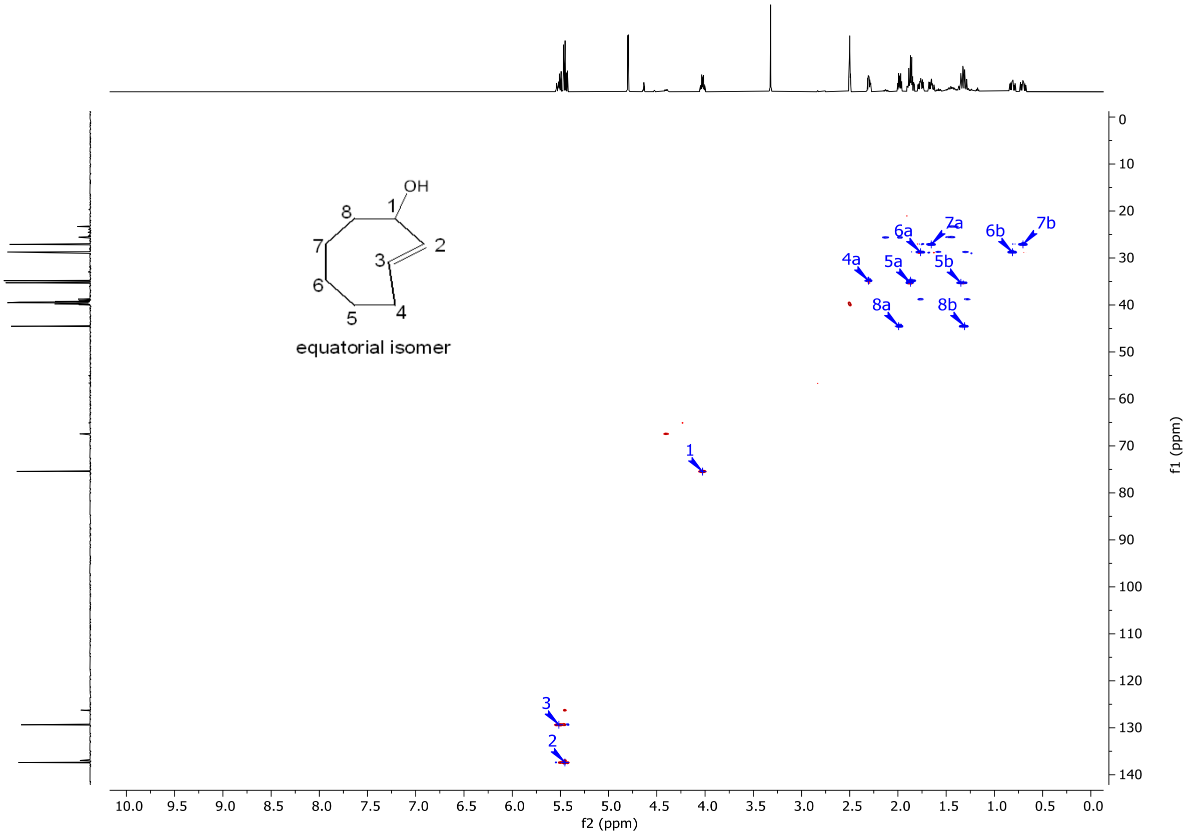

Figure S47: ^{1}H-^{13}C HSQC spectrum of equatorial *trans*-cyclooctenol in DMSO-*d6*.

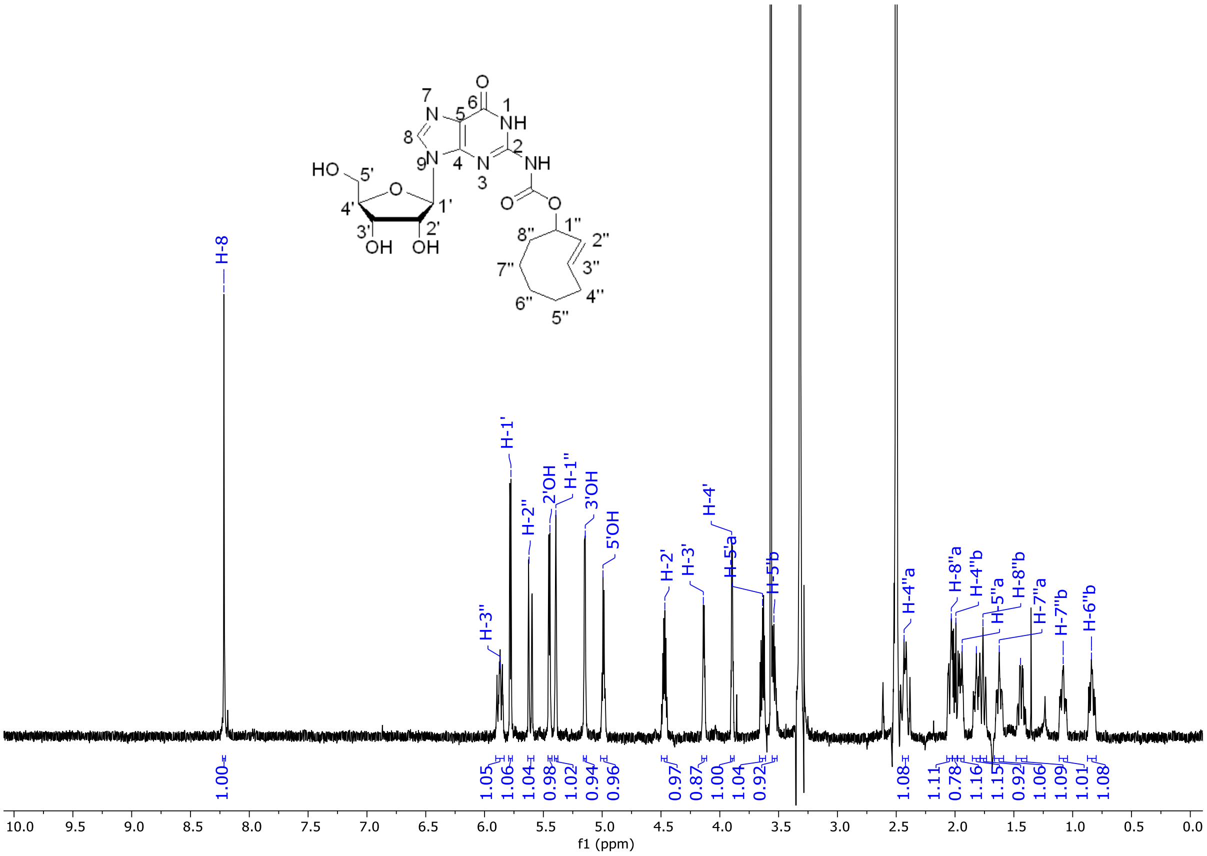

Figure S48: ^{1}H NMR of compound **1** in DMSO-*d6*.

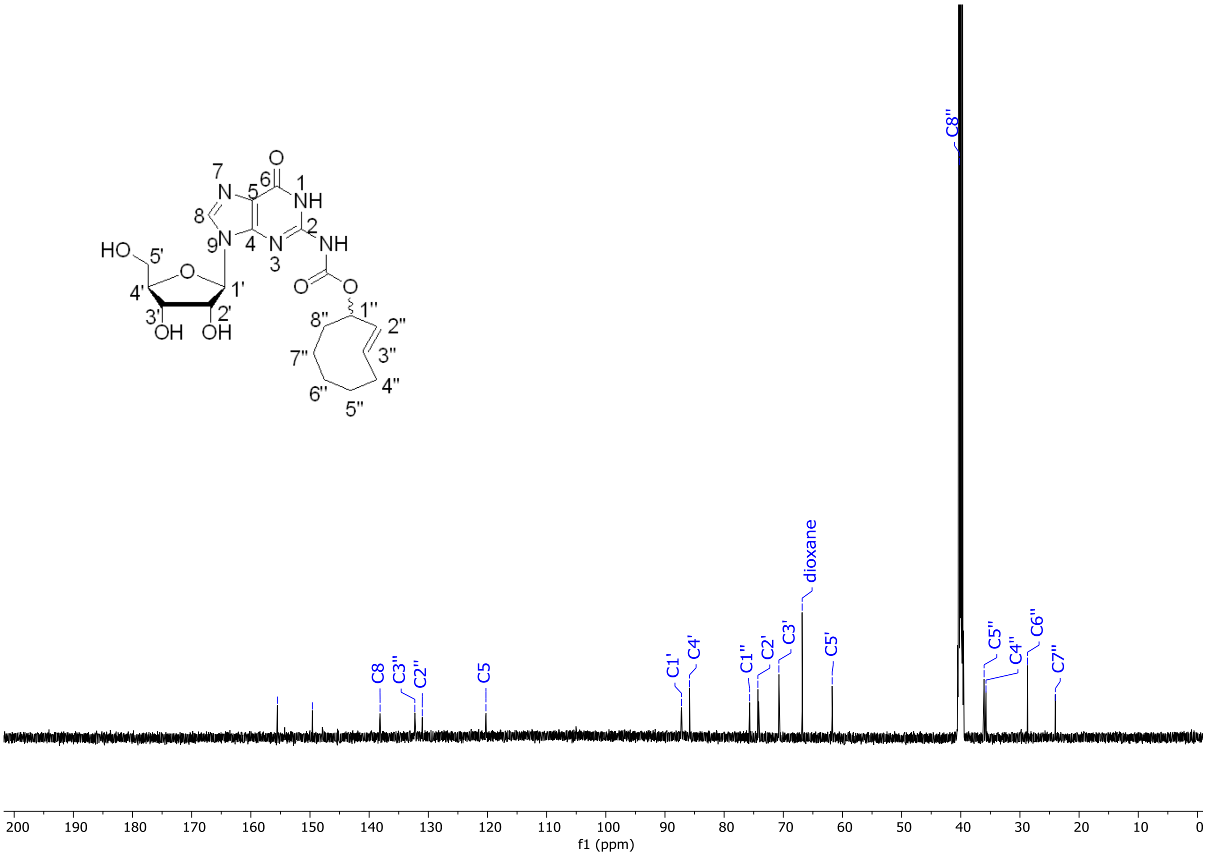

Figure S49: ^{13}C NMR of compound **1** in DMSO-*d6*.

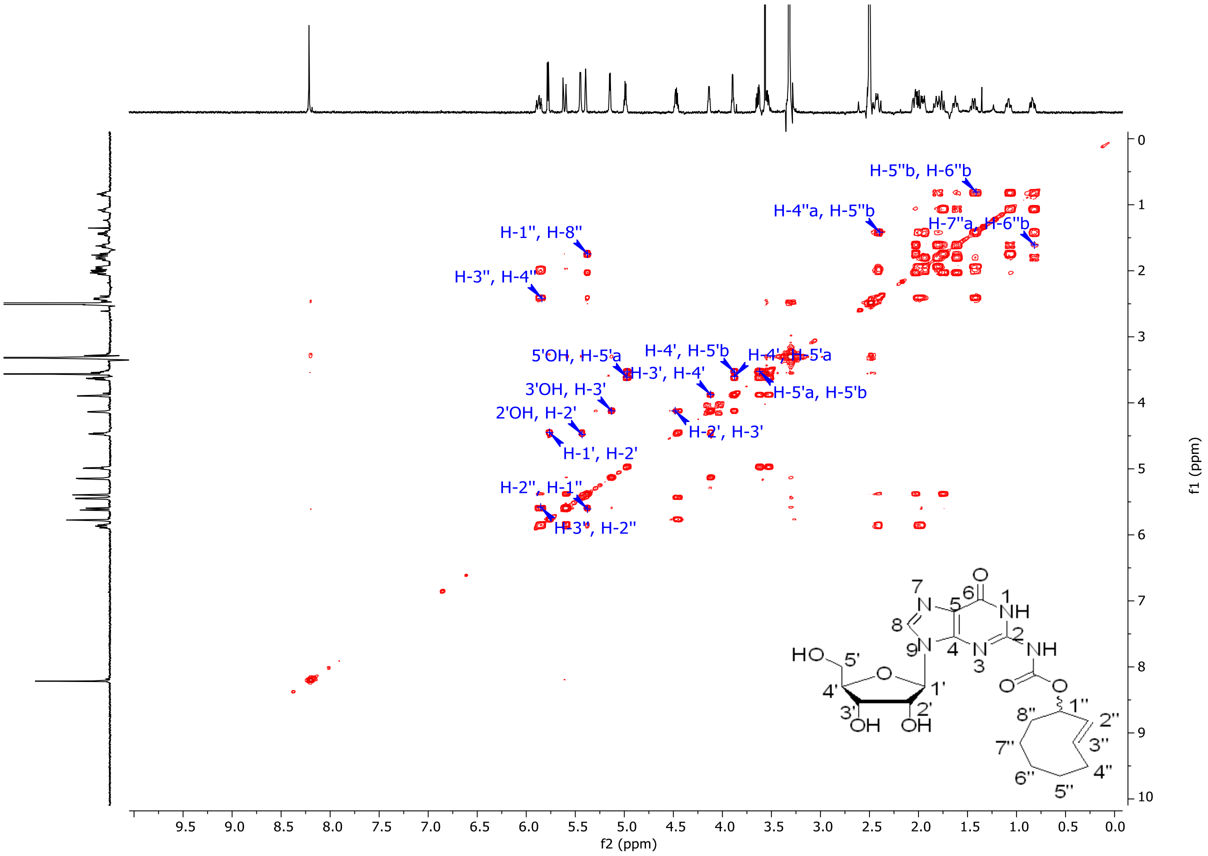

Figure S50: ^{1}H COSY spectrum of compound **1** in DMSO-*d6*.

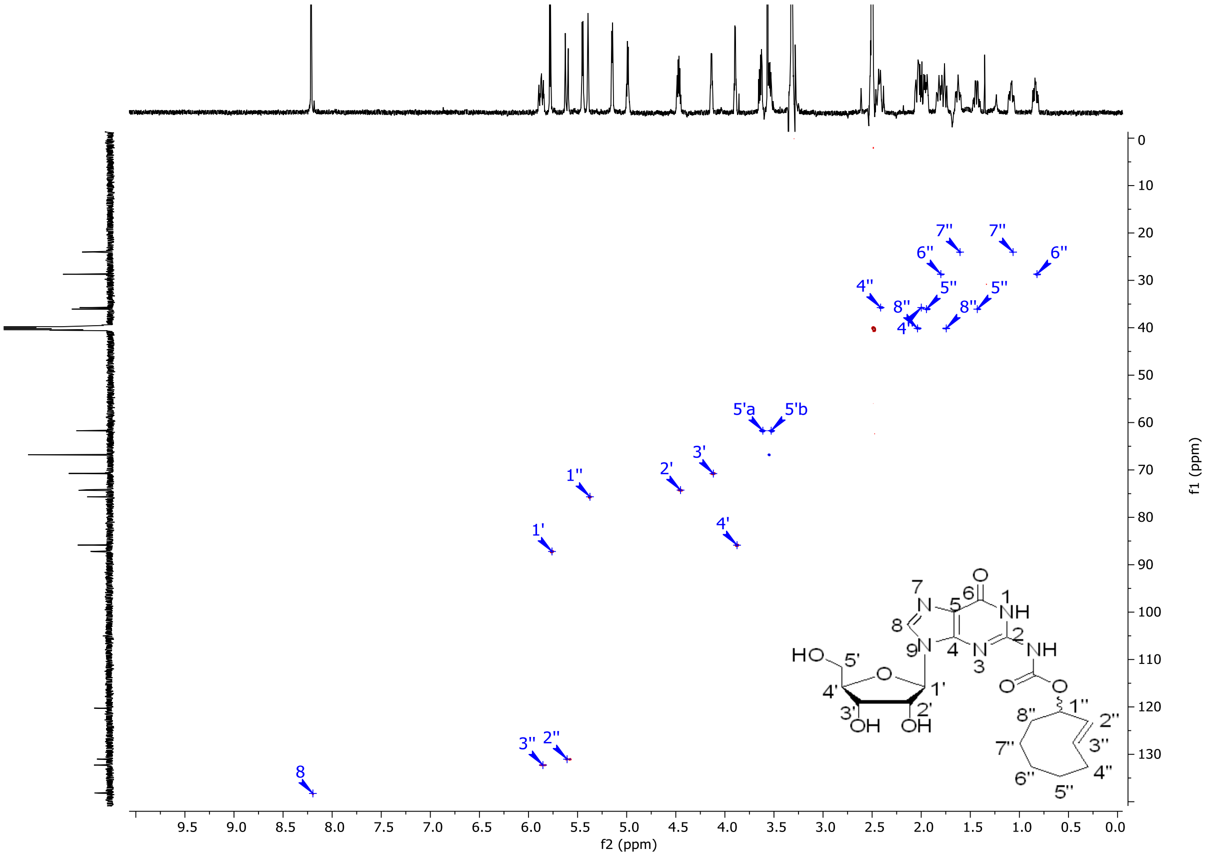

Figure S51: ^{1}H-^{13}C HSQC spectrum of compound **1** in DMSO-*d6*.

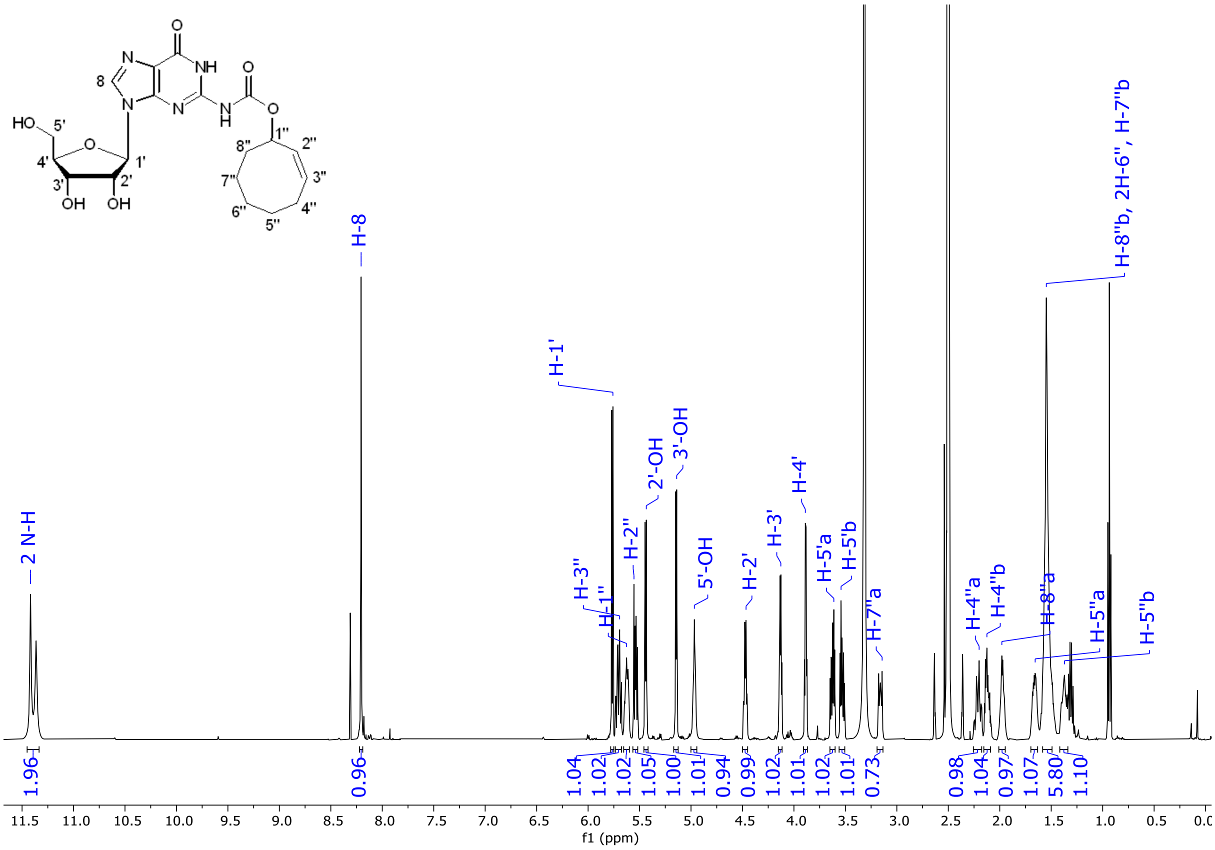

Figure S52: ^{1}H NMR spectrum of *cis*-cyclooctene-guanosine in DMSO-*d6*.

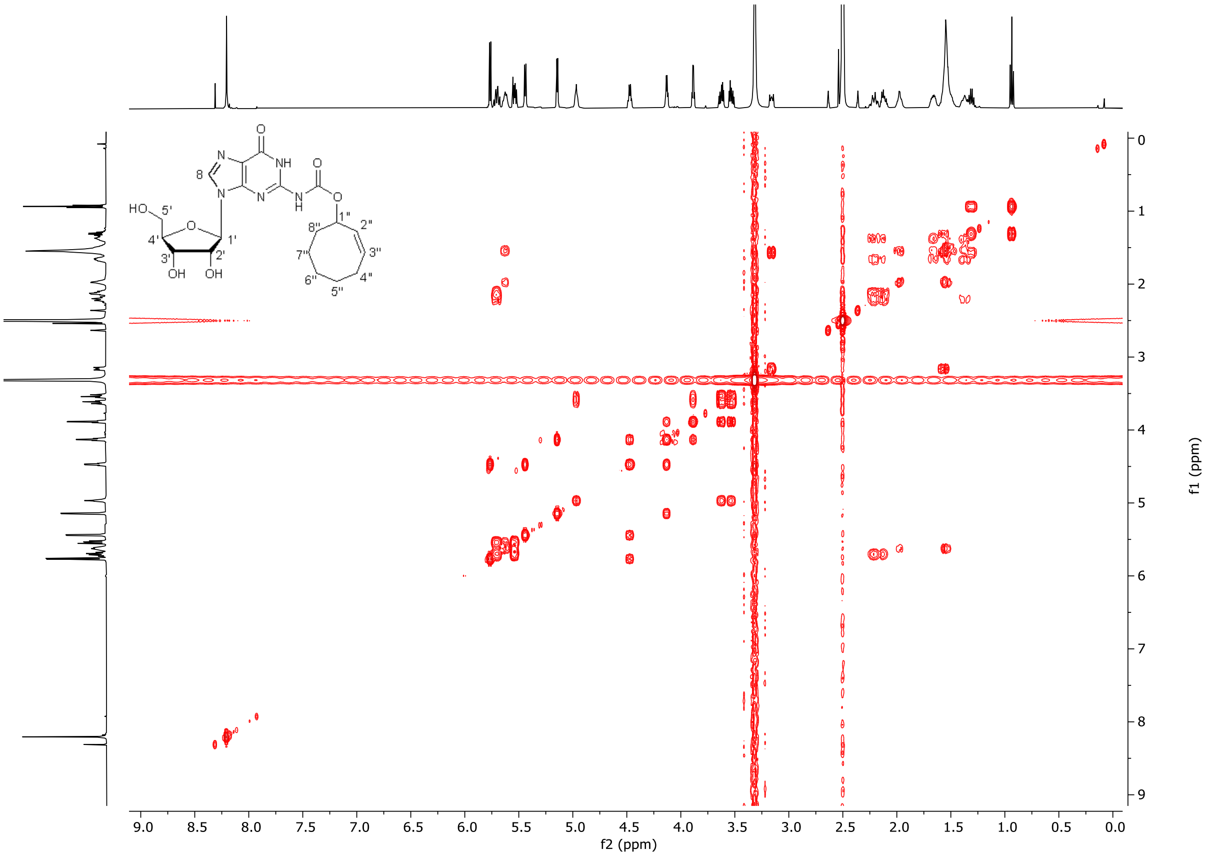

Figure S53: ^{1}H COSY spectrum of *cis*-cyclooctene-guanosine in DMSO-*d6*.

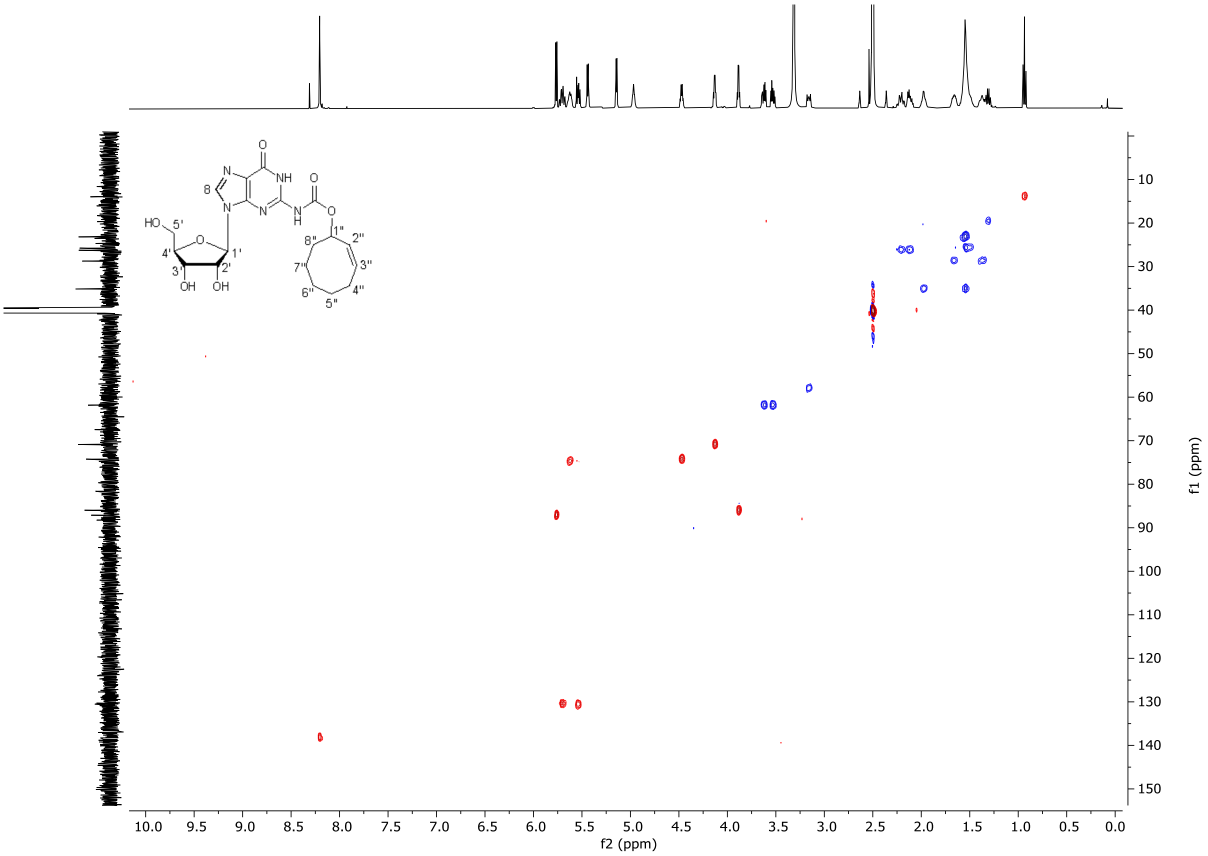

Figure S54: ^{1}H-^{13}C HSQC spectrum of *cis*-cyclooctene-guanosine in DMSO-*d6*.

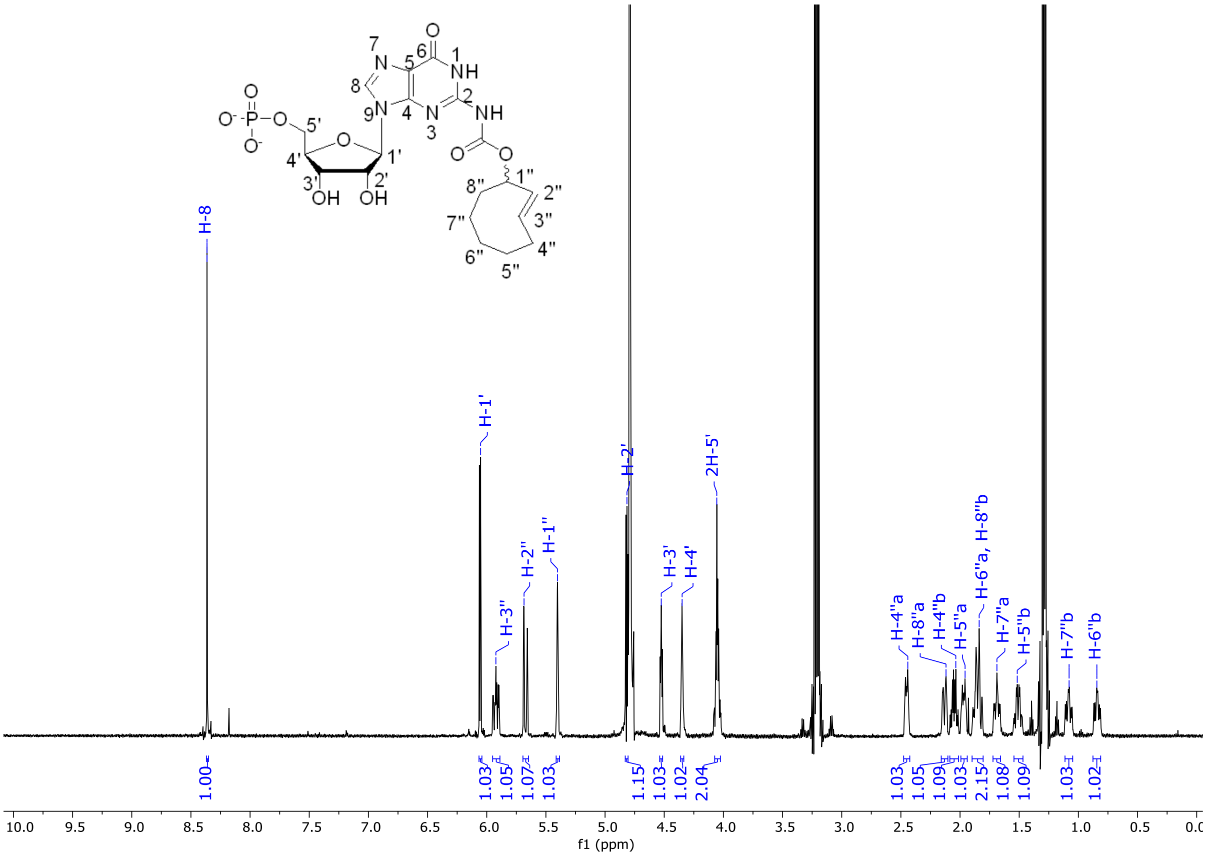

Figure S55: ^{1}H NMR of compound **2** in D_2O.

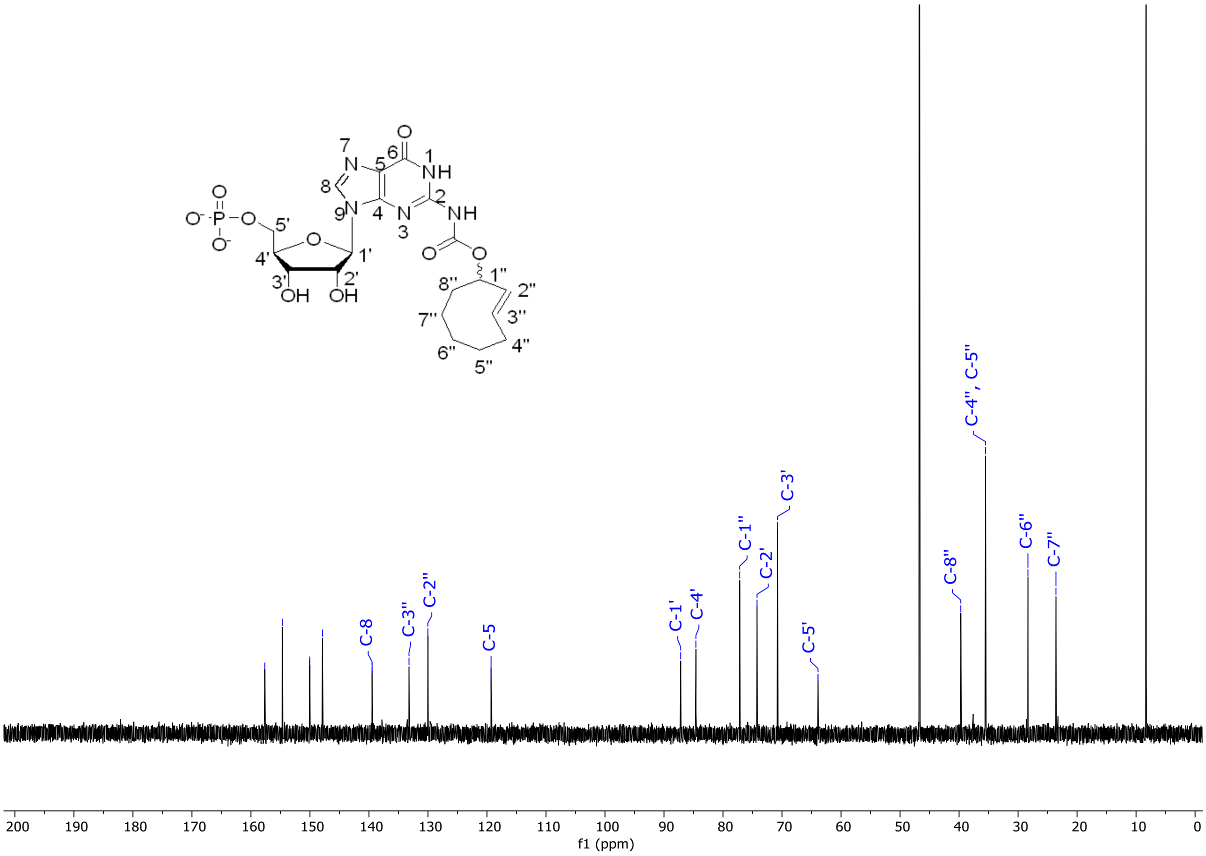

Figure S56: ^{13}C NMR of compound **2** in D_2O.

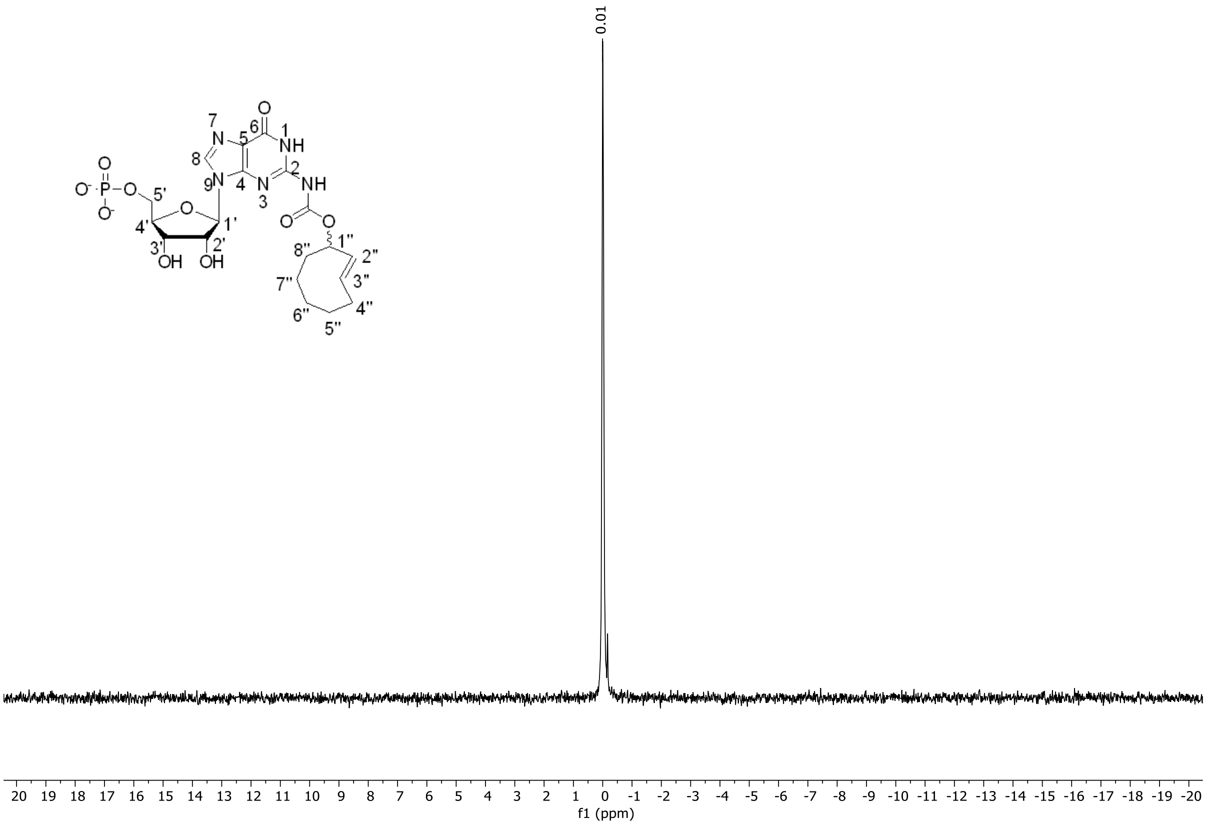

Figure S57: Decoupled ^{31}P NMR of compound **2** in D_2O.

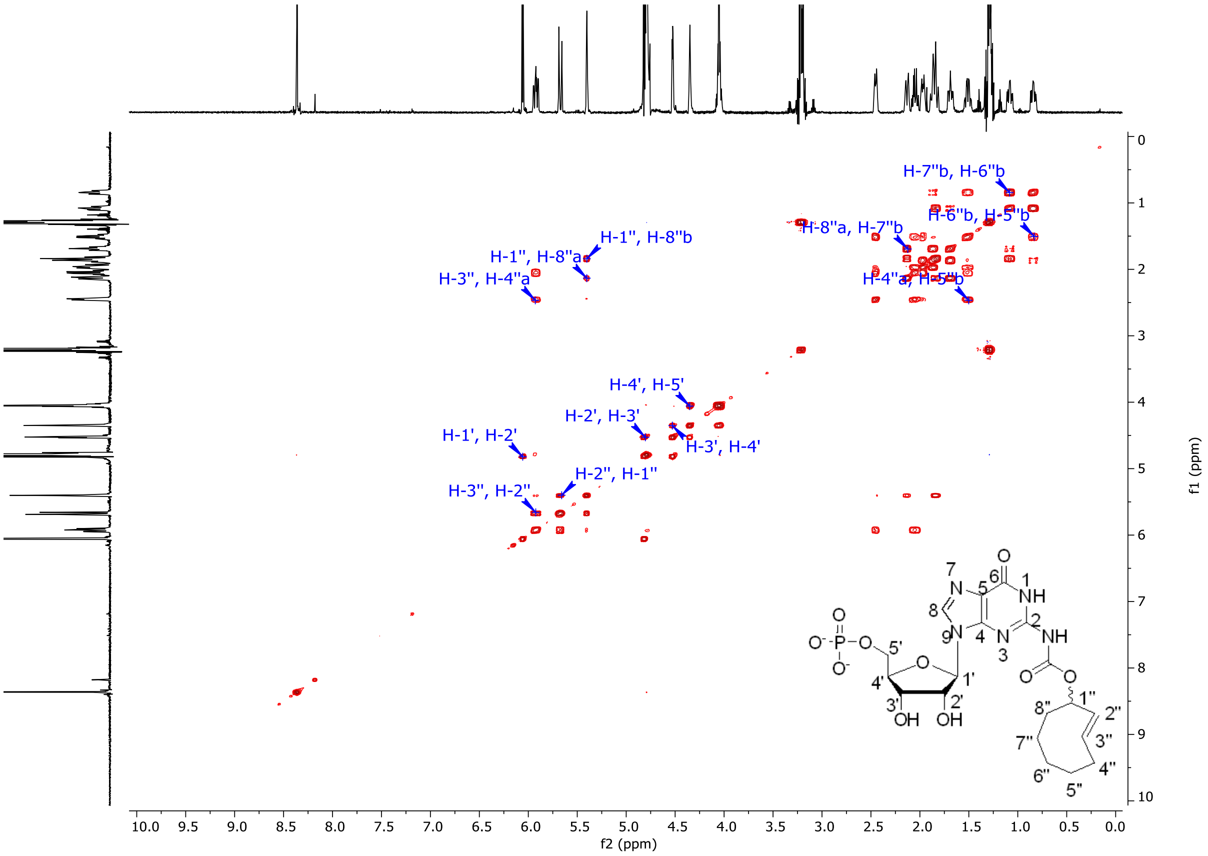

Figure S58: ^1H COSY spectrum of compound **2** in D_2O.

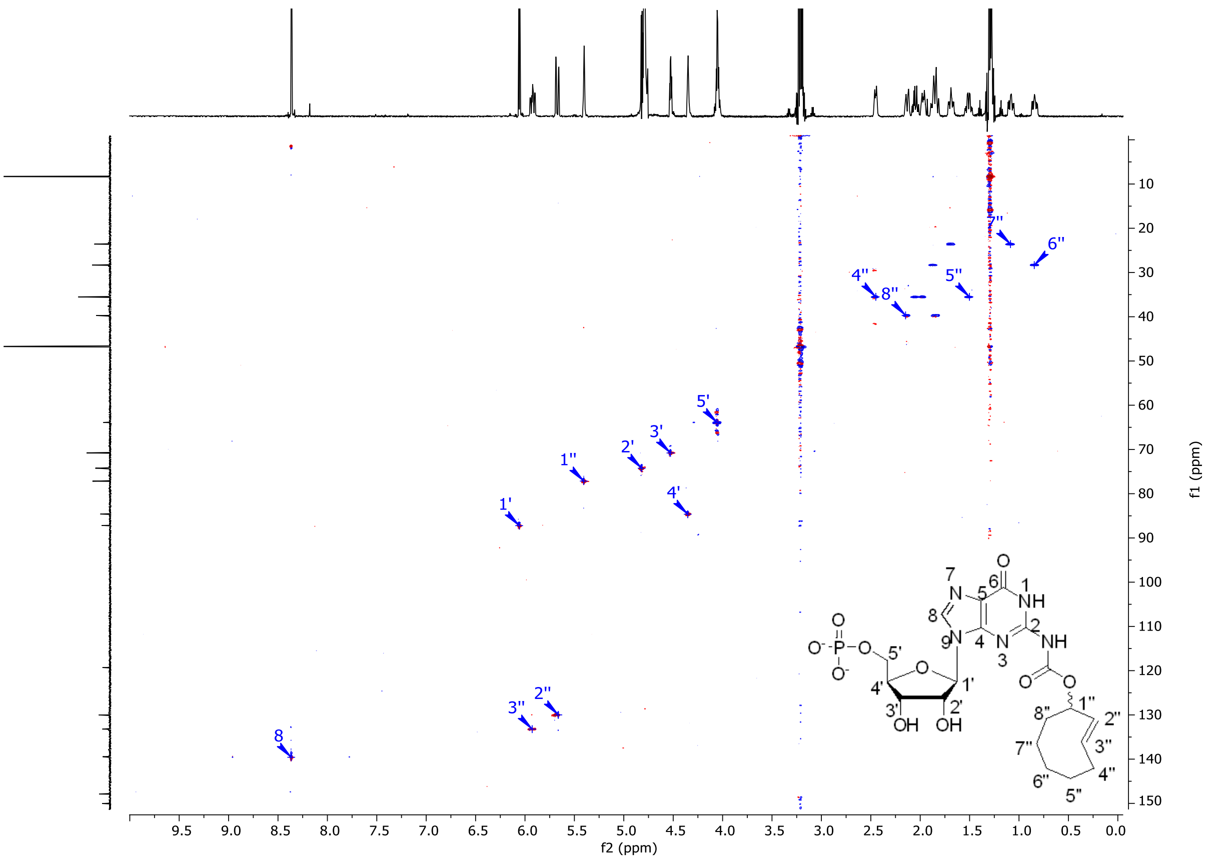

Figure S59: ^{1}H-^{13}C HSQC spectrum of compound **2** in D_2O.

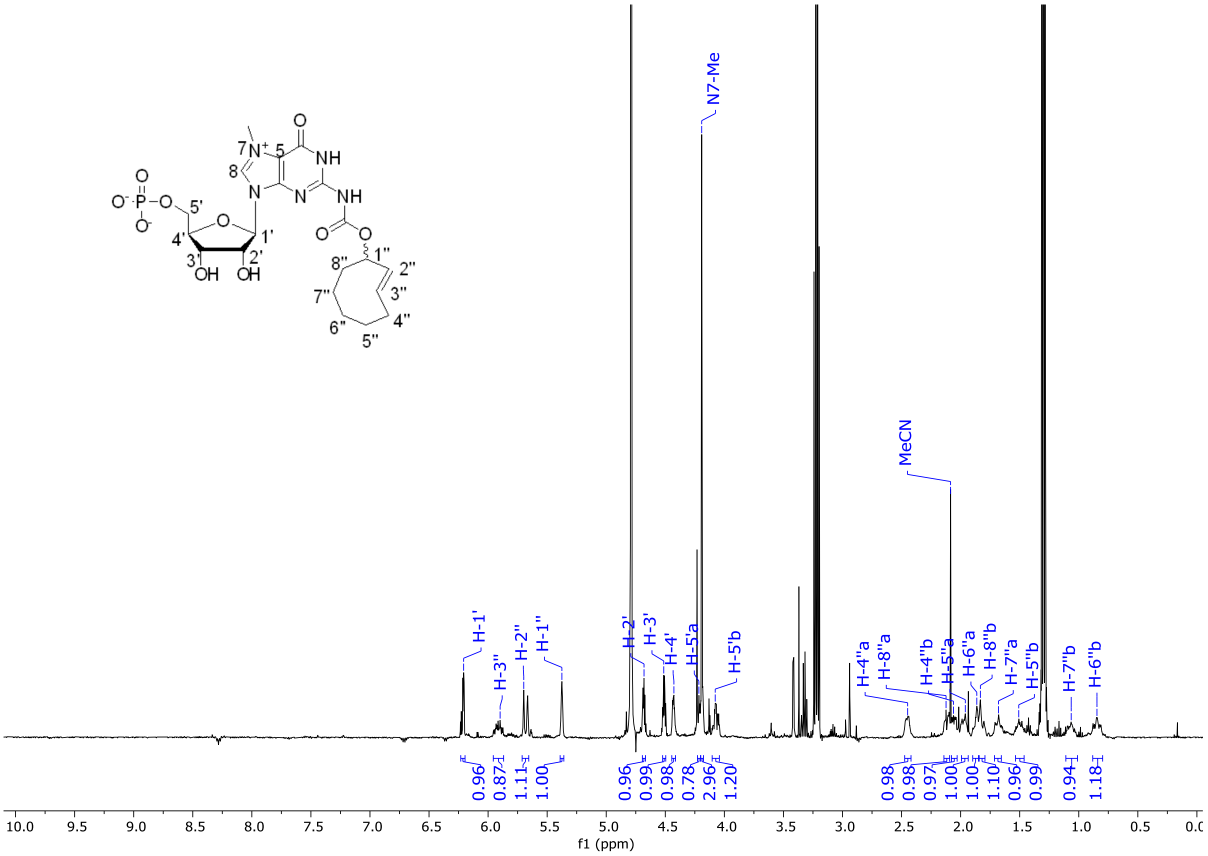

Figure S60: ^{1}H NMR of compound **3** in D_2O.

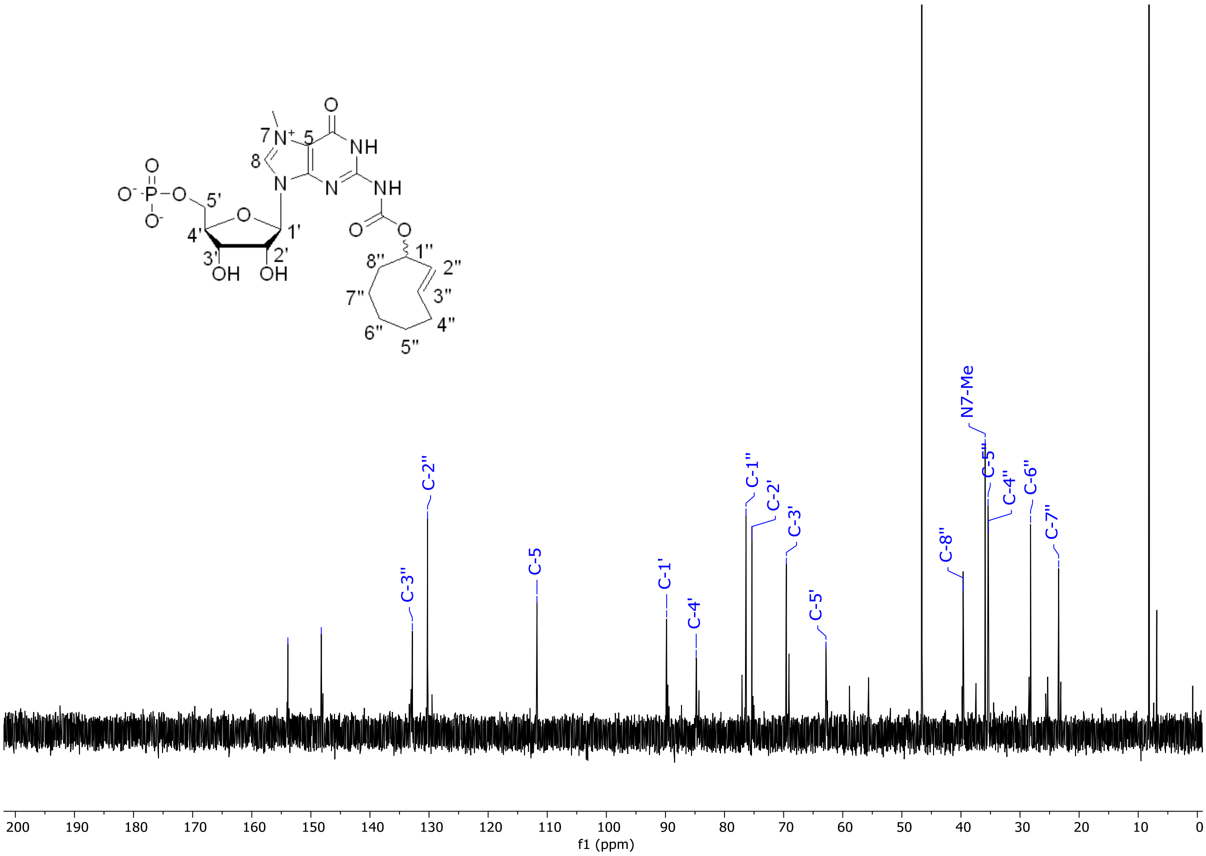

Figure S61: ^{13}C NMR of compound **3** in D_2O.

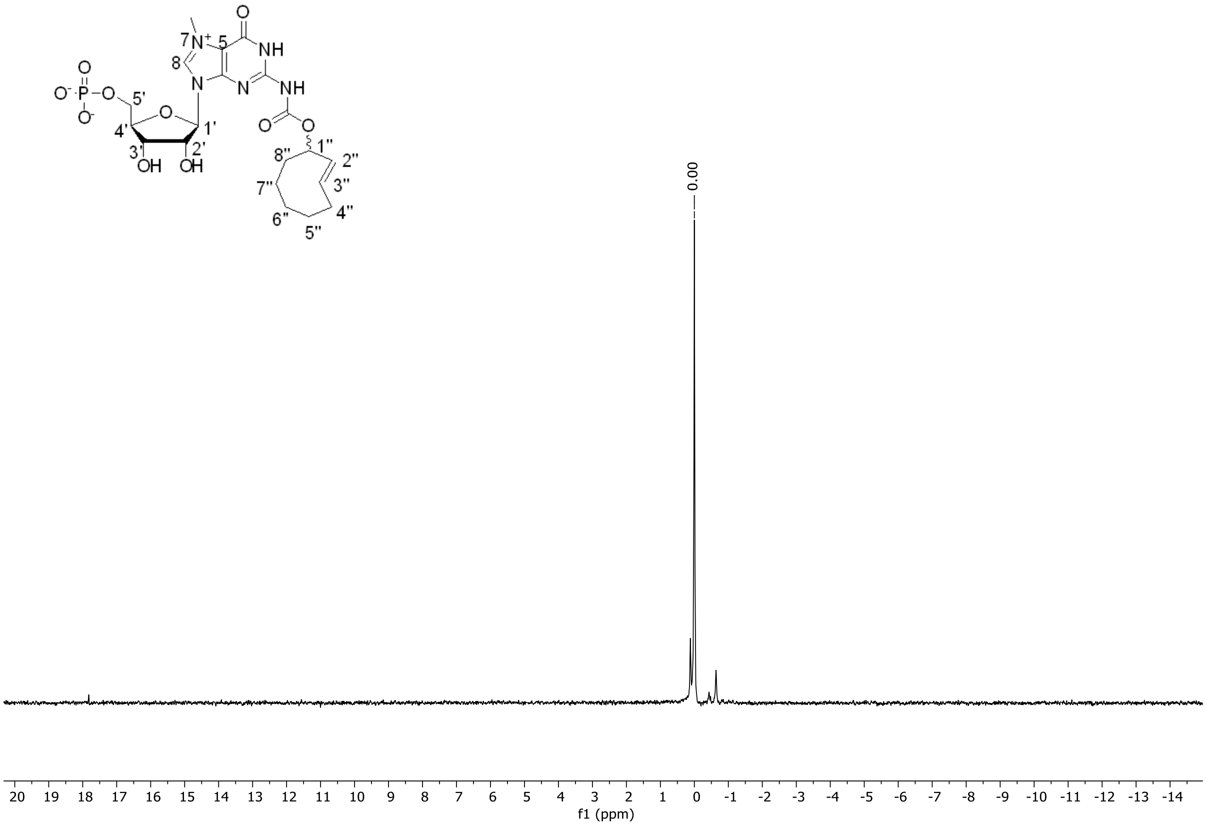

Figure S62: Decoupled ^{31}P NMR of compound **3** in D_2O.

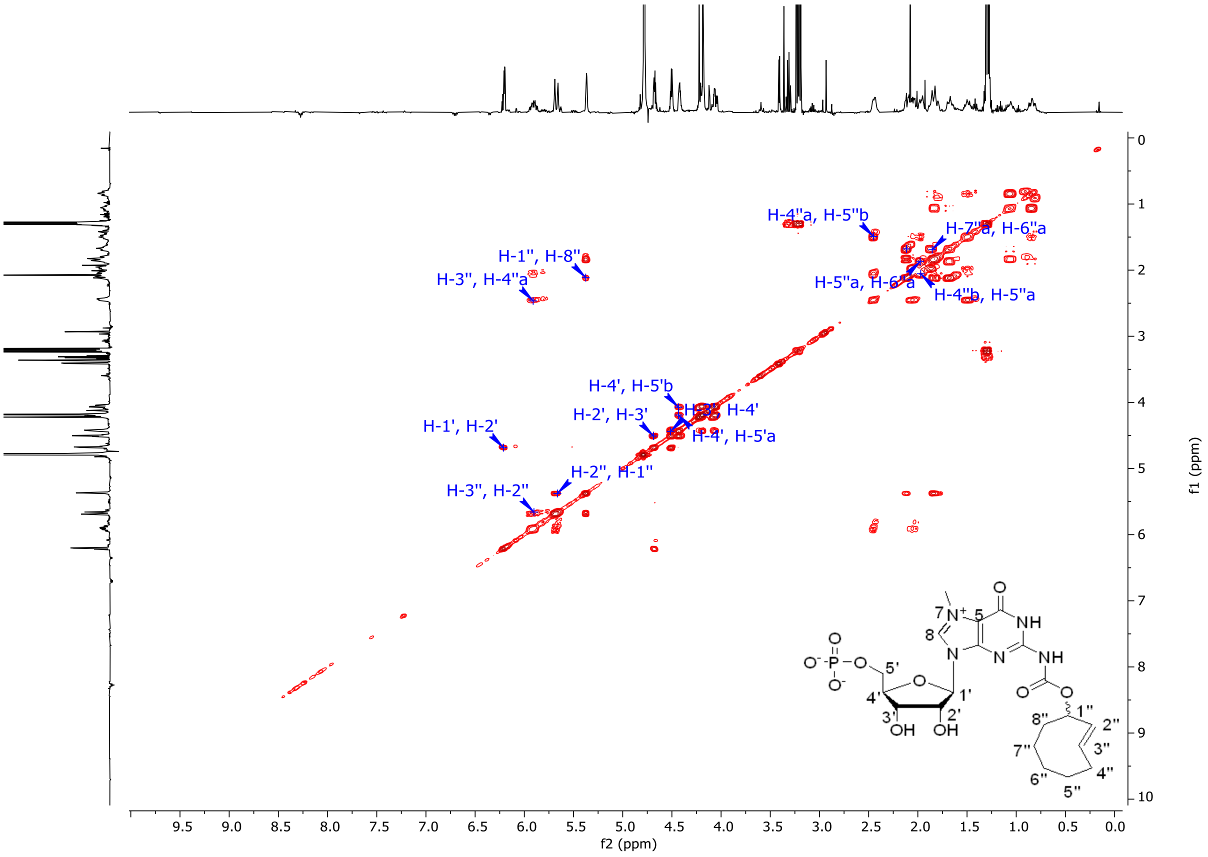

Figure S63: ^{1}H COSY spectrum of compound **3** in D_2O.

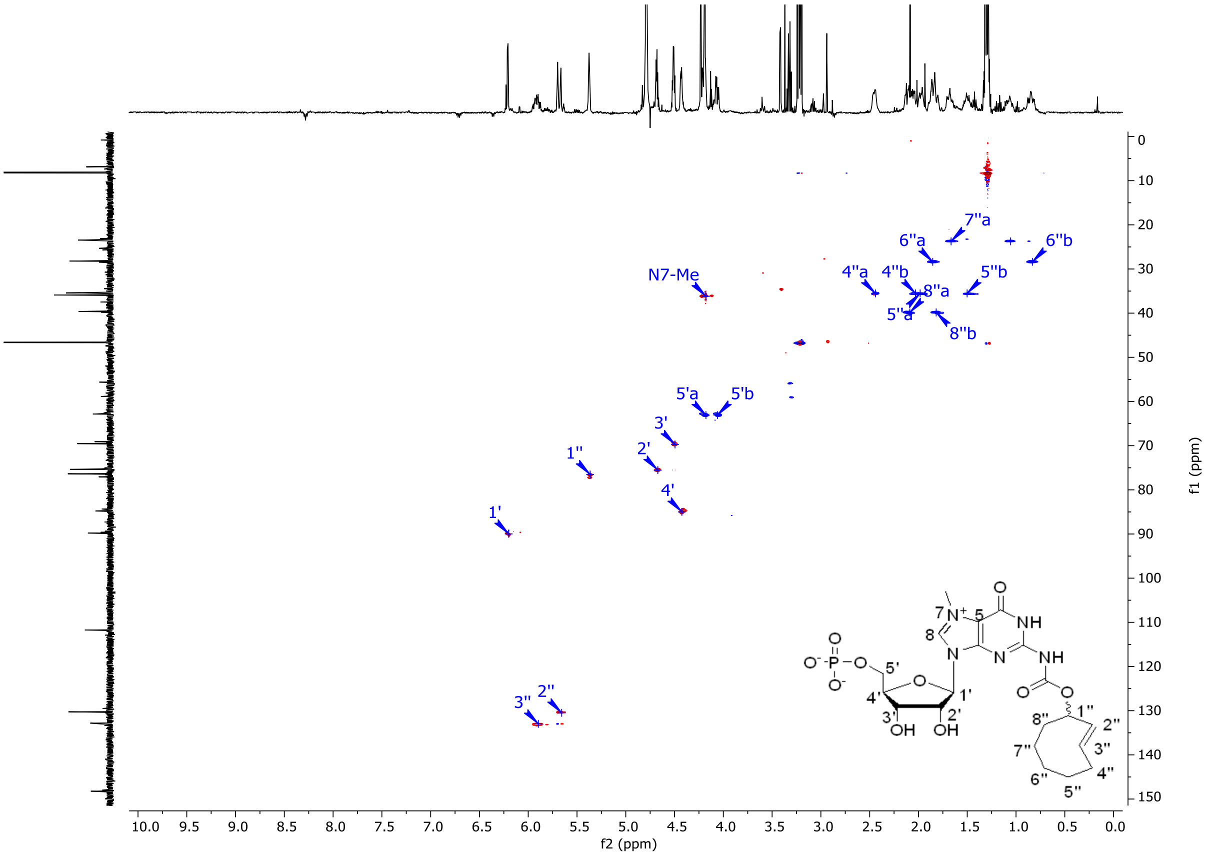

Figure S64: ^{1}H-^{13}C HSQC spectrum of compound **3** in D_2O.

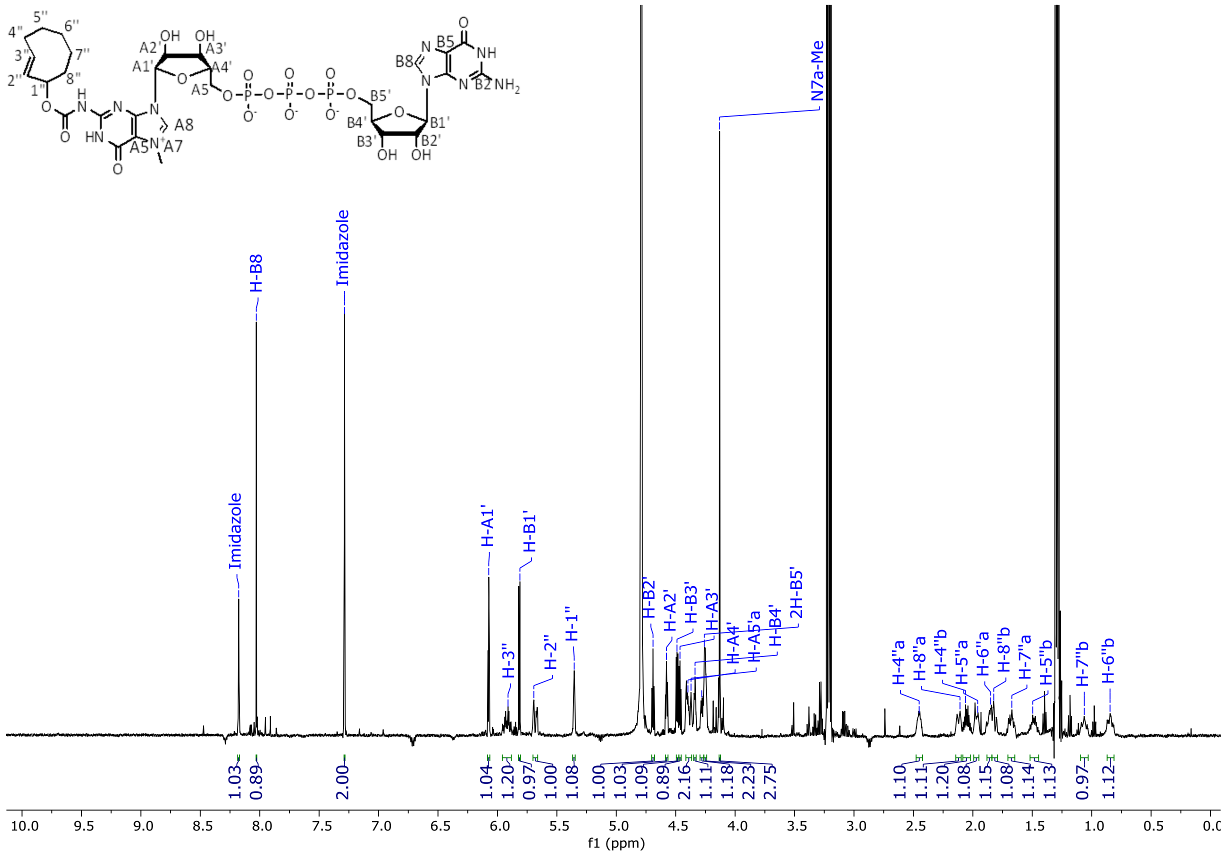

Figure S65: ^{1}H NMR of compound **4** in D_2O.

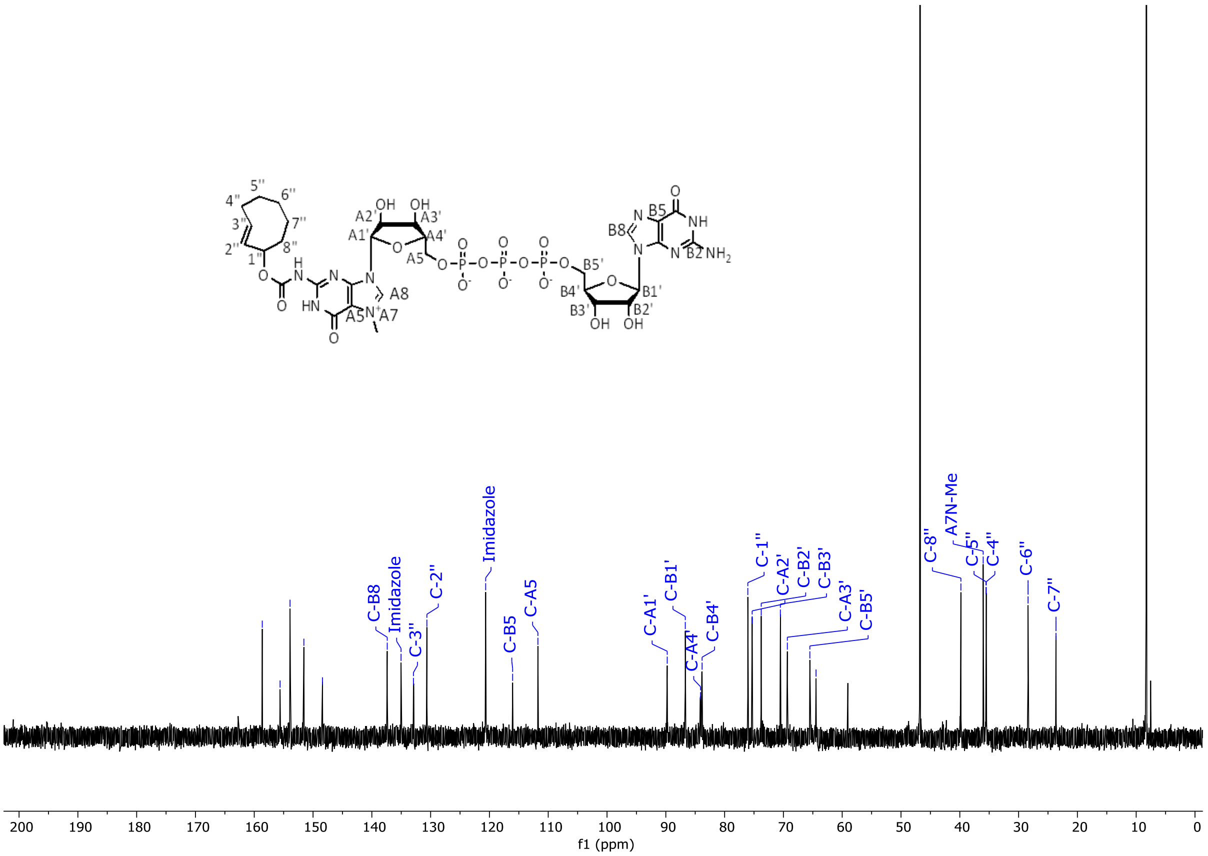

Figure S66: ^{13}C NMR of compound **4** in D_2O.

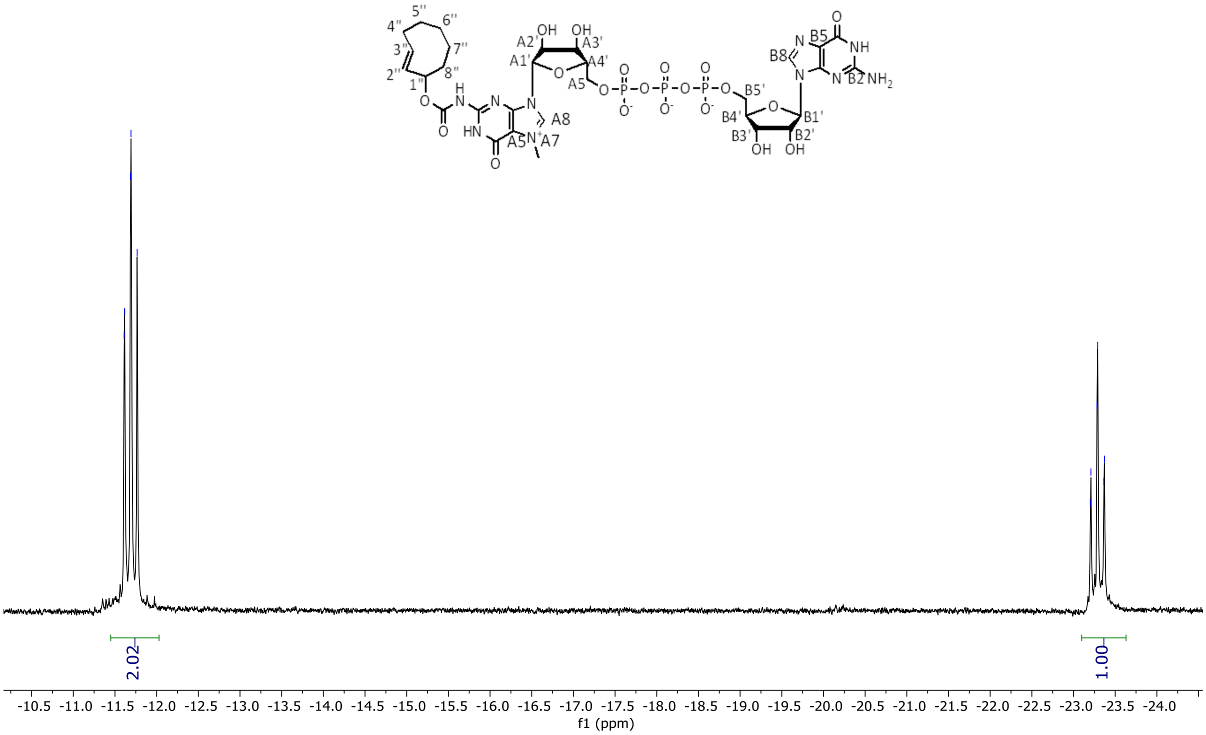

Figure S67: Decoupled ^{31}P NMR of compound **4** in D_2O.

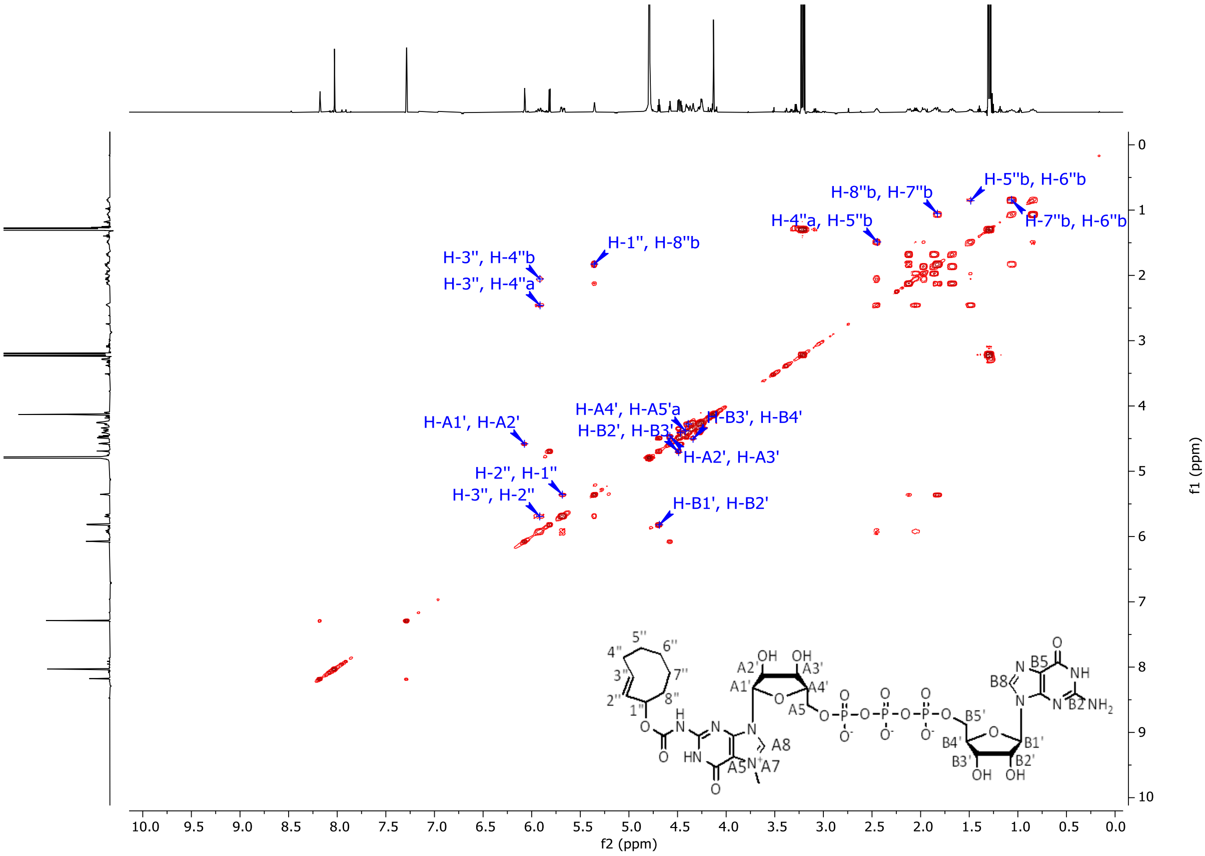

Figure S68: ^{1}H COSY spectrum of compound **4** in D_2O.

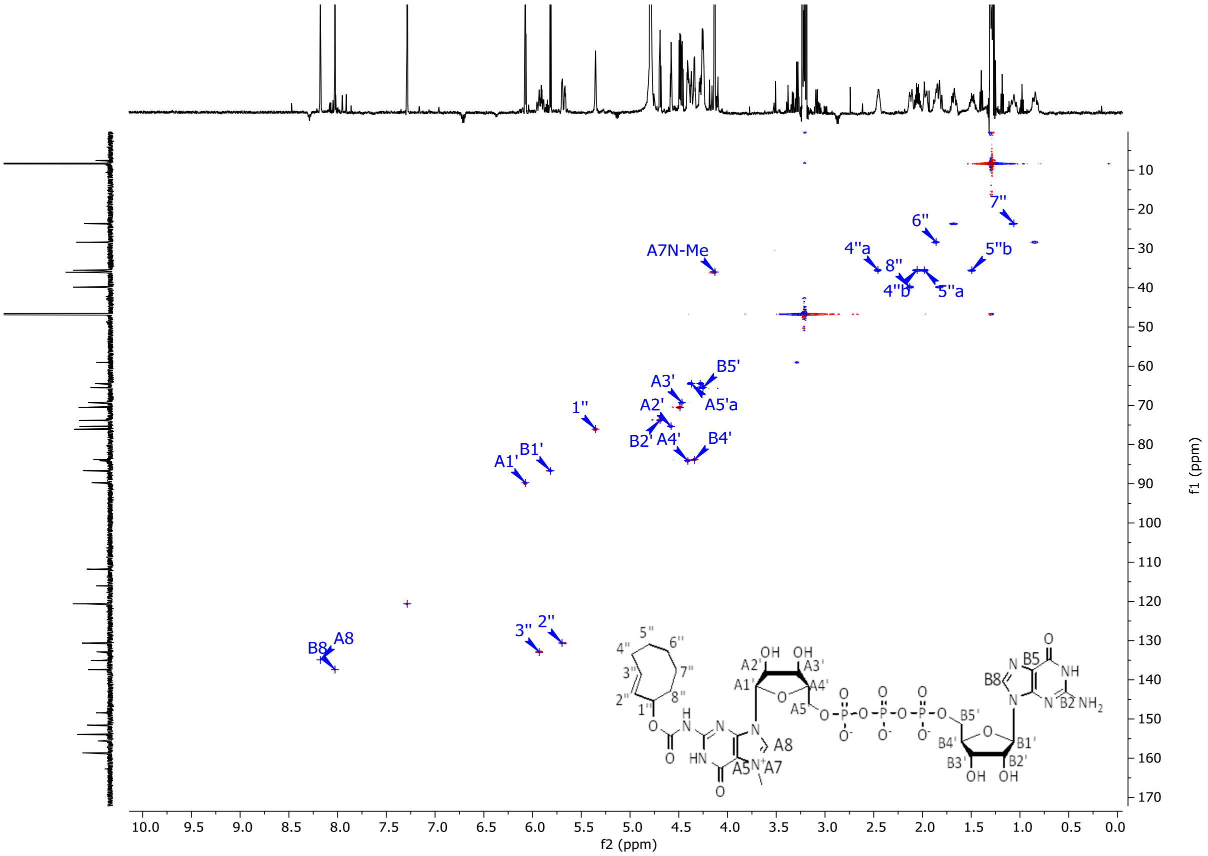

Figure S69: ^{1}H-^{13}C HSQC spectrum of compound **4** in D_2O.

6. References

[1] J. Jemielity, T. Fowler, J. Zuberek, J. Stepinski, M. Lewdorowicz, A. Niedzwiecka, R. Stolarski, E. Darzynkiewicz, R. E. Rhoads, *RNA (New York, N.Y.)* **2003**, *9*, 1108.

[2] M. A. R. de Geus, G. J. M. Groenewold, E. Maurits, C. Araman, S. I. van Kasteren, *Chemical science* **2020**, *11*, 10175.

[3] A. Darko, S. Wallace, O. Dmitrenko, M. M. Machovina, R. A. Mehl, J. W. Chin, J. M. Fox, *Chemical science* **2014**, *5*, 3770.

[4] a) *The PyMOL Molecular Graphics System, Version 3.0 Schrödinger, LLC.*; b) K. TOMOO, X. SHEN, K. OKABE, Y. NOZOE, S. FUKUHARA, S. MORINO, T. ISHIDA, T. TANIGUCHI, H. HASEGAWA, A. TERASHIMA et al., *Biochemical Journal* **2002**, *362*, 539.
